# Supplementary material for: Enablers and barriers to treatment adherence in heterozygous familial hypercholesterolaemia: a qualitative evidence synthesis
Source: BMJ Open. 2019 Jul 31;9(7):e030290. doi: 10.1136/bmjopen-2019-030290 (PMC6677970; doi:10.1136/bmjopen-2019-030290)
Supplement: Supplementary data [file bmjopen-2019-030290supp001.pdf]

Supplementary File 1: Deviations from protocol

Stated in protocol	What we did	Rationale for deviation
'Only studies in which the full text is available in English will be eligible for inclusion'	We did not place any limits upon language of included papers	We aimed to overcome the recognised restrictions of individual qualitative study findings, by gathering and examining a wide range of patient perceptions and experiences. After an initial scope of the available evidence base in this population group, it was apparent that the number of potential papers to be retrieved would be manageable by the research team. Therefore, the decision was made to remove this exclusion criteria, in order to identify all relevant evidence in line with the comprehensive searching approach to be taken in this review. This is in line with available guidance which advises that language filter decisions should be made in reference to the aims of the review.[28]
'The participants include individuals aged ≥ 10 years'	We did not place any limits upon age of included participants.	The database searching retrieved a paper reporting findings from a sample which included children aged 8 years. As it was not possible to extract the data from only participants aged 10 years and older, using the original inclusion criteria the paper would have to be excluded from the synthesis. This paper was one of only 3 papers retrieved that reported findings from samples including children, therefore the findings were perceived to be very valuable to the synthesis. Children are often diagnosed with FH before the age of 10, and U.K. and international guidance advise treatment with lifestyle advice, with lipid lowering therapy to be implemented when they reach a suitable age.[11] Furthermore, it is stated in the NICE guidelines that lipid lowering drug treatment should be commenced by the age of 10 and statin therapy can be considered at 8-10 years of age.
'Both stages of data extraction will be carried out independently by two reviewers (AS, FJK)…'	First stage of data extraction (study details) was carried out by two reviewers (JC, FJK) and second stage (study findings) by two reviewers (AS, FJK).	This was to split work between review members.
'The two reviewers (FJK, AS) will then work in collaboration to develop initial descriptive themes and categories based upon the raw data…'	Three reviewers (FJK, AS, EW) worked in collaboration for the second two stages of thematic synthesis.	EW joined review team after publication of protocol. We felt having a further expert opinion from a health psychologist would improve the synthesis output.
'The findings are intended to be used in the development of future intervention or guidelines….'	The findings are presented with a focus on informing clinical practice	The findings were interpreted to be of particular importance to clinical practice. While the findings are still useful to intervention and guideline development, this paper will focus upon their application in a clinical setting,

Kinnear FJ, *et al. BMJ Open* 2019; 9:e030290. doi: 10.1136/bmjopen-2019-030290
