# Supplementary material for: Enablers and barriers to treatment adherence in heterozygous familial hypercholesterolaemia: a qualitative evidence synthesis
Source: BMJ Open. 2019 Jul 31;9(7):e030290. doi: 10.1136/bmjopen-2019-030290 (PMC6677970; doi:10.1136/bmjopen-2019-030290)
Supplement: Supplementary data [file bmjopen-2019-030290supp002.pdf]

**Supplementary File 2- ENTREQ reporting guidelines checklist**

ENTREQ: Enhancing transparency in reporting the synthesis of qualitative research [38]

| No | Item | Guide and description | Reported on page # |
|---|---|---|---|
| **1** | Aim | State the research question the synthesis addresses. | Page 4 & 5 |
| **2** | Synthesis methodology | Identify the synthesis methodology or theoretical framework which underpins the synthesis, and describe the rationale for choice of methodology (e.g. meta-ethnography, thematic synthesis, critical interpretive synthesis, grounded theory synthesis, realist synthesis, meta-aggregation, meta-study, framework synthesis). | Protocol and page 7 |
| **3** | Approach to searching | Indicate whether the search was pre-planned (comprehensive search strategies to seek all available studies) or iterative (to seek all available concepts until they theoretical saturation is achieved). | Protocol, page 6 and supplementary file 3 |
| **4** | Inclusion criteria | Specify the inclusion/exclusion criteria (e.g. in terms of population, language, year limits, type of publication, study type). | Protocol and page 6 |
| **5** | Data sources | Describe the information sources used (e.g. electronic databases (MEDLINE, EMBASE, CINAHL, psycINFO, Econlit), grey literature databases (digital thesis, policy reports), relevant organisational websites, experts, information specialists, generic web searches (Google Scholar) hand searching, reference lists) and when the searches conducted; provide the rationale for using the data sources. | Protocol, page 6 and supplementary file 3 |
| **6** | Electronic Search strategy | Describe the literature search (e.g. provide electronic search strategies with population terms, clinical or health topic terms, experiential or social phenomena related terms, filters for qualitative research, and search limits). | Protocol, page 6 and supplementary file 3 |
| **7** | Study screening methods | Describe the process of study screening and sifting (e.g. title, abstract and full text review, | Protocol and page 7 |

| No | Item | Guide and description | Reported on page # |
|---|---|---|---|
| | | number of independent reviewers who screened studies). | |
| **8** | Study characteristics | Present the characteristics of the included studies (e.g. year of publication, country, population, number of participants, data collection, methodology, analysis, research questions). | Page 8, table 1 and supplementary file 4 |
| **9** | Study selection results | Identify the number of studies screened and provide reasons for study exclusion (e,g, for comprehensive searching, provide numbers of studies screened and reasons for exclusion indicated in a figure/flowchart; for iterative searching describe reasons for study exclusion and inclusion based on modifications t the research question and/or contribution to theory development). | Figure 1 and page 8 |
| **10** | Rationale for appraisal | Describe the rationale and approach used to appraise the included studies or selected findings (e.g. assessment of conduct (validity and robustness), assessment of reporting (transparency), assessment of content and utility of the findings). | Protocol, pages 6-7 and supplementary file 4 |
| **11** | Appraisal items | State the tools, frameworks and criteria used to appraise the studies or selected findings (e.g. Existing tools: CASP, QARI, COREQ, Mays and Pope[25]; reviewer developed tools; describe the domains assessed: research team, study design, data analysis and interpretations, reporting). | Protocol, pages 6 and supplementary file 4 |
| **12** | Appraisal process | Indicate whether the appraisal was conducted independently by more than one reviewer and if consensus was required. | Protocol, pages 6-7, supplementary file 4 |
| **13** | Appraisal results | Present results of the quality assessment and indicate which articles, if any, were weighted/excluded based on the assessment and give the rationale. | Page 12, Table 1 & supplementary file 4 |



Kinnear FJ, *et al. BMJ Open* 2019; 9:e030290. doi: 10.1136/bmjopen-2019-030290

| No | Item | Guide and description | Reported on page # |
|---|---|---|---|
| **14** | Data extraction | Indicate which sections of the primary studies were analysed and how were the data extracted from the primary studies? (e.g. all text under the headings *"results /conclusions" were extracted electronically* and entered into a computer software). | Protocol and page 7 |
| **15** | Software | State the computer software used, if any. | Protocol and page 7 |
| **16** | Number of reviewers | Identify who was involved in coding and analysis. | Page 7 and supplementary file 5 |
| **17** | Coding | Describe the process for coding of data (e.g. line by line coding to search for concepts). | Page 7 and supplementary file 5 |
| **18** | Study comparison | Describe how were comparisons made within and across studies (e.g. subsequent studies were coded into pre-existing concepts, and new concepts were created when deemed necessary). | Supplementary file 5 |
| **19** | Derivation of themes | Explain whether the process of deriving the themes or constructs was inductive or deductive. | Supplementary file 5 |
| **20** | Quotations | Provide quotations from the primary studies to illustrate themes/constructs, and identify whether the quotations were participant quotations of the author's interpretation. | Table 2 and pages 13-16 |
| **21** | Synthesis output | Present rich, compelling and useful results that go beyond a summary of the primary studies (e.g. new interpretation, models of evidence, conceptual models, analytical framework, development of a new theory or construct). | Table 4 and pages 22-25 |



Kinnear FJ, *et al. BMJ Open* 2019; 9:e030290. doi: 10.1136/bmjopen-2019-030290
