# Supplementary material for: Enablers and barriers to treatment adherence in heterozygous familial hypercholesterolaemia: a qualitative evidence synthesis
Source: BMJ Open. 2019 Jul 31;9(7):e030290. doi: 10.1136/bmjopen-2019-030290 (PMC6677970; doi:10.1136/bmjopen-2019-030290)
Supplement: Supplementary data [file bmjopen-2019-030290supp003.pdf]

Supplementary File 3- Full details of search strategy

Full details are available in the published protocol[37] but are detailed briefly below.

Search Strategy

MEDLINE, Embase, PsycINFO (via OVID), Cochrane library and CINAHL databases were searched from inception to 05/09/2018. We used a validated qualitative search filter[(121)] and population specific search terms. The search strategy that was used in MEDLINE is displayed in Appendix 1. The OpenGrey database and specialist websites (HEART UK, British Heart Foundation, The FH Foundation and The Simon Broome Register) were also searched up until 05/09/2018. The reference lists of the 50 papers taken to the full text screening stage were also hand searched. When only an abstract was available, the lead author was conducted in attempt to retrieve the full text. When contacting the lead authors of the included papers as part of the quality appraisal stage, enquiries were also made about any unpublished work.

Appendix 1: Search Strategy used in MEDLINE 05/09/2018

1. (familial adj1 hypercholesterolemia).ti,ab, kf.

2. (familial adj1 hypercholesterolaemia).ti,ab, kf.

3. (inherit* adj1 high adj1 cholesterol).ti,ab, kf.

4. *Hypercholesterolemia/ge [Genetics]

5. 1 or 2 or 3 or 4

6. interview*.ti,ab.

7. exp. Interviews/

8. experience*.tw.

9. qualitative.ti,ab.

10. 6 or 7 or 8 or 9

11. 5 and 10

DRAFT

Kinnear FJ, *et al. BMJ Open* 2019; 9:e030290. doi: 10.1136/bmjopen-2019-030290
