# Supplementary material for: Enablers and barriers to treatment adherence in heterozygous familial hypercholesterolaemia: a qualitative evidence synthesis
Source: BMJ Open. 2019 Jul 31;9(7):e030290. doi: 10.1136/bmjopen-2019-030290 (PMC6677970; doi:10.1136/bmjopen-2019-030290)
Supplement: Supplementary data [file bmjopen-2019-030290supp004.pdf]

Supplementary File 4- Quality appraisal methodology and results

Methodology

The CASP tool, endorsed by the Cochrane Collaboration,[41] asks 10 questions relating to the rigour of the methodology used, quality of reporting and relevance of findings. To ensure comprehensive evaluation of methodological quality, these questions were answered with further consideration of 12 criteria produced by an expert panel.[112] As the purpose of the quality appraisal was to determine the methodological strengths and limitations of studies included in the synthesis, the lead authors of each paper were contacted to obtain further information in an attempt to overcome the recognised issued of poor reporting in qualitative research. Information from multiple papers involving the same sample was pooled when appropriate. Each author was given 1 month to respond. Two reviewers (AS, FK) independently appraised each study, assigning a rating of 0, 1 or 2 for each question which reflected the extent to which the obtained information from paper and author answered the criteria (0=not addressed, 1=partially addressed, 2=fully addressed). The reviewers then met to come to a consensus of individual and total scores, resolving differences through discussion. The reviewers then decided upon threshold for low, medium and high rated quality that they felt adequately captured the quality of the included papers.

Summary of results

Table 1 displays the CASP score breakdowns for each paper. Table 2 displays further details of the methodological limitations and transferability considerations of each included paper.

DRAFT

Kinnear FJ, *et al. BMJ Open* 2019; 9:e030290. doi: 10.1136/bmjopen-2019-030290

Table 1: CASP appraisal scores of included studies

Sample number	Reference	Was there a clear statement of research aims?	Is qualitative methodology appropriate?	Was the research design appropriate to address the aims of the research?	Was the recruitment strategy appropriate to the aims of the research?	Were the data collected in a way that addressed the research issue?	Has the relationship between researcher and participants been adequately considered?	Have ethical issues been taken into consideration?	Was the data analysis sufficiently rigorous?	Is there a clear statement of findings?	Is the research valuable?	Overall score (out of 20)	Did author provide further information?
**1**	Agard et al, 2005[49]	2	2	2	2	2	0	1	0	1	1	**13**	**NO**
**2**	DeAngelis et al, 2017[50]	2	2	2	1	1	1	0	1	1	2	**13**	**NO**
**3**	Frich, 2007[46]	2	2	2	2	2	2	2	2	2	2	**20**	**YES**
	Frich et al, 2006[62]	2	2	2	2	2	2	2	2	1	2	**19**	**YES**
	Frich et al, 2007[63]	2	2	2	1	2	2	2	2	2	1	**18**	**YES**
	Frich et al, 2007[24]	2	2	2	2	2	2	2	2	2	2	**20**	**YES**
**4**	Hallowell et al, 2017[51]	2	2	2	2	2	1	2	1	1	2	**17**	**YES**
	Jenkins et al, 2013[53]	2	2	2	1	2	0	2	1	1	2	**15**	**YES**
	Jenkins et al, 2013[52]	2	2	2	1	2	0	2	1	0	1	**13**	**YES**
**5**	Hardcastle et al, 2015[23]	2	2	2	2	2	1	2	1	2	2	**18**	**YES**
**6**	Hollands et al, 2012[54]	2	2	2	2	0	0	2	1	1	1	**13**	**NO**
**7**	Hollman et al, 2004[55]	2	2	2	2	2	1	2	2	2	2	**19**	**YES**
**8**	Keenan et al, 2018[56]	2	2	2	2	2	0	2	1	1	2	**16**	**YES**
**9**	Kirkegaard et al, 2014[57]	2	2	2	2	2	1	2	2	1	1	**17**	**YES**

DRAFT

**10**	Mackie et al, 2015[58]	2	2	2	2	2	2	1	2	2	2	**19**	**NO**
	Sliwinski et al, 2017[25]	2	2	2	2	2	2	1	2	2	2	**19**	**NO**
**11**	Meulenkamp et al, 2008[59]	2	2	2	2	2	1	2	2	2	2	**19**	**YES**
**12**	Mortensen et al, 2008[60]	2	2	2	1	1	0	0	1	1	1	**11**	**NO**
**13**	Urke, 2016[47]	2	2	2	2	2	2	2	2	2	2	**20**	**NO**
**14**	Weiner, 2006[48]	2	2	2	1	2	1	2	1	2	2	**18**	**YES**
	Weiner and Durrington, 2008[26]	2	2	2	1	2	1	2	1	2	2	**17**	**YES**
	Weiner, 2009[64]	2	2	2	1	2	1	2	1	1	1	**16**	**YES**
	Weiner, 2011[65]	2	2	2	1	2	1	2	1	1	2	**16**	**YES**
**15**	Senior et al, 2002[61]	2	2	2	2	1	0	0	1	1	1	**12**	**NO**

Scoring system: 0=No criteria fulfilled or can't tell; 1= some criteria fulfilled; 2= All criteria fulfilled. In reference to the criteria suggested for each question by CASP tool(53) and further criteria as described by Santiago-Delefosse et al.(122)

DRAFT

Table 2: Summary of methodological limitations and transferability considerations of the included papers

Sample number	Reference	CASP quality SCORE & rating	Methodological and reporting limitations	Transferability considerations of sample
**1**	Agard et al, 2005[49]	13 Low	Lack of details provided about the rigour of the analysis process.<br>Authors self-selected the data from interviews to transcribe.<br>Data saturation not discussed.<br>Relationship between researcher and participants was not adequately considered.<br>Credibility of findings and the limitations of study design not addressed when reporting the findings.<br>No details of informed consent or if participants were told about data confidentiality or their right to withdraw.	No sampling strategy used but sample comprised of a good range of ages, genders, history of CVD events and age of diagnosis.<br>All recruited from one clinic.<br>All from Sweden.
**2**	DeAngelis et al, 2017[50]	13 Low	Ethical issues not addressed.<br>Group meetings may have resulted in lack of representative findings as certain individuals may have dominated the conversations or individuals may have felt unable to voice their own opinions.<br>Lack of disconfirming cases presented<br>No details of informed consent or if participants were told about data confidentiality or their right to withdraw.	All very motivated and engaged individuals to volunteer for this group.<br>Many receiving apheresis treatment.
**3**	Frich, 2007[46]	20 high	Study limitations not addressed when reporting the findings.	All motivated to seek treatment as active attendees of lipid clinic.<br>Majority young (70% 10-39 years) and asymptomatic.<br>Large (40) sample size.<br>All from Norway.<br>All recruited from one lipid clinic.
	Frich et al, 2006[62]	19 High	Lack of disconfirming cases presented and discussion against the findings.	
	Frich et al, 2007[63]	18 High	Lack of disconfirming cases presented and discussion against the findings.	
	Frich et al, 2007[24]	20 High	Study limitations not addressed when reporting the findings.	
**4**	Hallowell et al, 2017[51]	17 Medium	Lack of details provided about the rigour of the analysis process<br>Relationship between researcher and participants was not adequately considered	All participants regularly attend lipid clinics and opted in for DNA testing.<br>Relatively well education (42% university education).<br>All participants from Scotland.<br>No sampling strategy used so likely not representative.<br>Half of patients from professional/skilled non-manual background.<br>Ethnicity not provided but authors state majority white British.<br>Recruited from two lipid clinics.
	Jenkins et al, 2013[53]	15 Medium	Credibility of findings and the limitations of study design not addressed when reporting the findings<br>Relationship between researcher and participants was not adequately considered.	
	Jenkins et al, 2013[52]	13 Low	Lack of details provided about the rigour of the analysis process.<br>Credibility of findings and the limitations of study design not addressed when reporting the findings.<br>Relationship between researcher and participants was not adequately considered.	
**5**	Hardcastle et al, 2015[23]	18 High	Analysis carried out by one individual only with no independent verification of themes conducted.<br>Relationship between researcher and participants was not adequately considered.	Sample not randomly selected.<br>Recruited from one clinic.<br>All live in metropolitan Perth, Australia.
**6**	Hollands et al, 2012[54]	13 Low	Lack of disconfirming cases presented and arguments against findings.<br>Data saturation not discussed.<br>Relationship between researcher and participants was not adequately considered.<br>Credibility of findings and the limitations of study design not addressed when reporting the findings.	Recruited across 11 lipid clinics.<br>All from the U.K.<br>All recently identified as being at risk of FH and during study received either clinical or DNA test results.

DRAFT

				Sample included in analysis includes participates with DNA positive and Non-DNA positive diagnosis.<br>Majority (14/19) white British.
**7**	Hollman et al, 2004[55]	19 High	Relationship between researcher and participants was not adequately considered.	All Swedish.<br>All recruited from one lipid clinic.
**8**	Keenan et al, 2018[56]	16 Medium	Credibility of findings and the limitations of study design not addressed when reporting the findings.<br>Relationship between researcher and participants was not adequately considered.<br>Analysis carried out by one individual.	All had consented to genetic testing.<br>All from Scotland.<br>All participants white, and majority highly educated.<br>Majority of participants asymptomatic.<br>Patients were self-selected from HCP who excluded participants if they felt they were too vulnerable, which included if had experienced a recent bereavement.<br>13 of parents had FH, 4 were spouses of those with FH
**9**	Kirkegaard et al, 2014[57]	17 Medium	Lack of results to support conclusions drawn.<br>Credibility of findings and the limitations of study design not addressed when reporting the findings.	All asymptomatic.<br>Only 2 FH patients and 1 relative of FH patient.
**10**	Mackie et al, 2015[58]	19 High	No details of informed consent or if participants were told about data confidentiality or their right to withdraw.	Most participants had private medical insurance, were white and all actively engaged with the healthcare system.<br>All recruited from same healthcare system.<br>All patients from Massachusetts, U.S.A.
	Sliwinski et al, 2017[25]	19 High	No details of informed consent or if participants were told about data confidentiality or their right to withdraw.	
**11**	Meulenkamp et al, 2008[59]	19 High	Relationship between researcher and participants was not adequately considered-three interviewers carried out the interviews and the potential bias this may incur was not addressed	All recruited from one health intuition.<br>All engaged with healthcare system and willing to talk about their condition.<br>11/16 were females.
**12**	Mortensen et al, 2008[60]	11 Low	Lack of details provided about the study methodology or rigour of analysis process.<br>Data saturation not discussed.<br>Relationship between researcher and participants was not adequately considered.<br>Credibility of findings and the limitations of study design not addressed when reporting the findings.<br>Ethical issues not addressed.	Half participants were reaching treatment goals, half were not.<br>All recruited from one genetic centre.<br>All Danish.<br>Only 1 female in the group of patients reaching treatment targets
**13**	Urke, 2016[47]	20 High	Coding and analysis of data was primarily independent, with the student's supervisors only overseeing it.	Sample comprised of non-attenders at clinic-not been seen for at least 2 years<br>Wide geographical spread, but all participants from Norway<br>Participants recruited from one clinic
**14**	Weiner, 2006[48]	18 High	Analysis by single researcher and potential bias not addressed<br>Data saturation not discussed.<br>Relationship between researcher and participants was not adequately considered.	Quota sampling used but all were white and majority (28/31) white British, 65% were ≥46 years old and 50% from professional occupations.<br>Participants recruited from one clinic.<br>Half self-reported experiencing some form of CHD.<br>All from North England, U.K.<br>All attended lipid clinic for at least 1 year, most for substantially longer.
	Weiner and Durrington, 2008[26]	17 Medium	Analysis by single researcher and potential bias not addressed.<br>Data saturation not discussed.<br>Relationship between researcher and participants was not adequately considered.<br>Credibility of findings not addressed when reporting the findings.	
	Weiner, 2009[64]	16 Medium	Analysis by single researcher and potential bias not addressed.<br>Data saturation not discussed.<br>Relationship between researcher and participants was not adequately considered.<br>Credibility of findings not addressed when reporting the findings.	
	Weiner, 2011[65]	16 Medium	Analysis by single researcher and potential bias not addressed.<br>Data saturation not discussed.<br>Relationship between researcher and participants was not adequately considered.<br>Credibility of findings not addressed when reporting the findings.	

DRAFT

**15**	Senior et al, 2002[61]	12 Low	Lack of details provided about the study methodology or rigour of analysis process.<br>Ethical issues not addressed.<br>Data saturation not discussed.<br>Credibility of findings or limitations of study methodology not addressed when reporting findings.<br>Relationship between researcher and participants was not adequately considered.	All motivated to participate in research as recruited from ongoing trial.<br>All lived in central London.<br>All clinical diagnosis, but 5 had DNA diagnosis confirmed and 2 had negative DNA test.

Additional references (To those listed in main manuscript)

112. Santiago-Delefosse M, Gavin A, Bruchez C, Roux P, Stephen SL. Quality of qualitative research in the health sciences: Analysis of the common criteria present in 58 assessment guidelines by expert users. Social Science & Medicine. 2016;148:142-5

DRAFT

Kinnear FJ, *et al. BMJ Open* 2019; 9:e030290. doi: 10.1136/bmjopen-2019-030290
