# Supplementary material for: Enablers and barriers to treatment adherence in heterozygous familial hypercholesterolaemia: a qualitative evidence synthesis
Source: BMJ Open. 2019 Jul 31;9(7):e030290. doi: 10.1136/bmjopen-2019-030290 (PMC6677970; doi:10.1136/bmjopen-2019-030290)
Supplement: Supplementary data [file bmjopen-2019-030290supp005.pdf]

DRAFT

**Supplementary File 5: Full details of thematic synthesis methodology**

Stage 1: Line by line coding

In our protocol we originally planned to analyse the extracted data according to our review questions regarding factors influencing adherence to treatment. However, few studies directly addressed this question, therefore the authors put these review questions to one side for the data extraction process and revisited them for the coding stage.

Two reviewers (AS, FJK) had previously read all papers independently for the critical appraisal stage. They were therefore familiar with the papers and had discussed them. At this stage they independently reread and coded, on paper, the extracted data from seven papers. The process involved line by line coding of the extracted data in which each line of text was assigned a free code according to its meaning and content. The codes were inductively created in response to the findings uncovered. The two reviewers then met to discuss and compare their findings before then deciding upon a preliminary coding frame which they then used when coding the extracted data from three further papers independently. In addition, new codes were created when necessary and the reviewers met again to discuss the findings, making revisions to the coding frame. One reviewer (FJK) then independently coded the extracted data from each paper using NVivo software. The coding frame was modified and added to throughout this process, with any changes made discussed with a second reviewer (AS). By this stage, no new codes were being identified, but some codes were consolidated into one code and others given more clarification about their meaning. A copy of the finalised coding frame is available to view (Appendix 1). A second reviewer (AS) performed secondary coding on 10% of the papers (three papers) before meeting with FJK to compare findings and ensure consistency of interpretation.

This line by line coding facilitated the translation of concepts from one study to another- a key component of qualitative synthesis. Most sentences were categorised using more than one code as a result of having content which had more than one possible meaning e.g. ‘perceived risk’ and ‘relative risk’ or ‘perceived seriousness’ and ‘emotional impact’.

Stage 2: Development of descriptive themes

This stage involves the development of initial descriptive themes based upon the raw data that closely reflect the aggregative findings of the included studies.[40] The two reviewers who had carried out the coding (AS, FJK) met with a third reviewer (EW) to discuss the findings



Kinnear FJ, *et al. BMJ Open* 2019; 9:e030290. doi: 10.1136/bmjopen-2019-030290

of the coding process. One reviewer (FJK) produced summary reports of each of the 19 identified free codes which provided an overview of the findings across the papers including illustrative quotes and disconfirming cases. The summaries were descriptive in nature and avoided any interpretation. These summaries formed the basis of discussion between the three reviewers. At this second stage, the discussion was carried out in the context of the first research question- what are the experiences and beliefs of individuals' in relation to their condition, its associated morbidity and mortality risk and treatment?'. The discussion was exploratory in nature and no priori framework was imposed upon the findings at this stage. The aggregative findings of the studies, as consolidated in the code summaries, were deliberated, with examination of any similarities, differences and relationships between codes explored. From this discussion, 20 descriptive themes were identified. These descriptive themes were reflective of prevalent and persistent findings across the studies. Some of these themes were reflective of original codes used in the coding process, others were new themes created to capture more specific and detailed aspects of the original findings of coding process. For example, the findings captured using the code 'family influence' were further categorised into the descriptive themes 'parental influence upon treatment related behaviours' and 'FH and its treatment becomes normalised within families'.

One reviewer (FJK) then produced a draft summary of these descriptive themes which was reviewed and discussed with AS and EW before a final version was agreed upon.

Stage 3: Development of analytical themes

The generated descriptive themes captured and aggregated the beliefs and experiences of individuals with FH in relation to their condition and its treatment. The third stage of thematic synthesis aims to go beyond the primary content of the original papers to generate additional concepts or understandings.[40] This is considered an essential component of any qualitative synthesis methodological approach.[42] In this review, this meant using the descriptive themes to answer our research questions regarding how these beliefs and experiences may influence an individuals' adherence to treatment and to identify any enablers and/or barriers to this.

This was achieved by first examining each descriptive theme individually in the context of treatment adherence through consideration of the relationship between the content captured in each descriptive theme and individuals ability and/or inclination to adhere to treatment. Secondly, any relationships between the descriptive themes were explored to identify

DRAFT

common factors. Each reviewer (FJK, AS, EW) carried this out independently before meeting as a group to discuss further. From these discussions, over-arching analytical themes were identified. These analytical themes were then deliberated in the context of identifying enablers and barriers to treatment adherence which could be used to inform clinical practice, policy development and research intervention design. The reviewers met on three occasions to discuss their findings collaboratively. It was an iterative process in which the analytical themes were modified until the reviewers felt they adequately explained all the initial descriptive themes and identified enablers and barriers to treatment.

For example, three of the descriptive themes related to the involvement of other family members in an individuals' experiences of having FH and its treatment (FH and its treatment become normalised within families, parental influence upon treatment related behaviours and desire to protect children). From these descriptive themes, the reviewers identified the importance of the behaviours and beliefs of other family members upon an individuals ability and receptivity to adhering to treatment. This finding was captured in the analytical theme entitled 'family influence'. From this analytical theme, the reviewers identified two enablers to treatment adherence. These enablers were the delivery of care and treatment advice through family-based clinics and the commencement of treatment from a young age.



Kinnear FJ, *et al. BMJ Open* 2019; 9:e030290. doi: 10.1136/bmjopen-2019-030290

Appendix 1: Finalised coding frame

| Code | Brief Description |
|---|---|
| Understanding/biological knowledge of FH | Account/description of what FH is, their understanding of its aetiology, its genetic transmission, its effect upon their body, any symptoms and any associated short and long term health implications |
| Perceived risk | The perceived risk of FH as a condition. Their thoughts/beliefs of short and long term health consequences of FH. Both genetic and behavioural associated risk. |
| Perceived seriousness | How serious/important FH and/or it's associated health consequences are believed to be |
| Family history | Account/description of family history of FH diagnosis, treatment and/or adverse outcomes such as death/serious illness |
| Life events | Significant milestones/occasions in life i.e. becoming parent, leaving school, getting married, ageing |
| Co-morbidities | Other illnesses/conditions that are not FH |
| Relative risk | Participant compares own risk to that of another person (family member, peer, abstract person) or to risk associated with another condition/illness |
| Management of condition | Account/description of the use/role of medicine or lifestyle in the treatment of FH. |
| Perceived efficacy of treatment | Perceptions/beliefs of the effectiveness of treating FH (medication, lifestyle and other) |
| Self-efficacy | The perception of an participant upon their own ability to follow treatment recommendations |
| Enablers and barriers for treatment | Any factors that help, enable, motivate OR Any factors that demotivate, stop or hinder a participant to seek and/or follow treatment advice |
| Ownership/personal responsibility | How a participant reflects/describes their perceived ownership of their condition and it's treatment. How much they perceive the condition to be their responsibility to manage/treat. |
| Emotional impact | Any emotion that FH diagnosis, management and/or associated health outcomes evokes in participants. Includes perceived stigma. |
| Impact on life | Any change participant has made to their life (everyday or longer term) as a result of their diagnosis of FH or its treatment |
| Professional support | Account/description of any involvement of healthcare professionals and/or medical procedures |
| Social support | Account/description of the role of family and/or friends in a patients' experience of their condition and it's management. practical or emotional support that individuals receive with regard to managing FH - i.e, treatment adherence. |
| Family influence | The influence of participants family upon their decision and ability to seek/adhere to treatment. Individuals' awareness of how others in their family network have dealt with screening and treatment and making decisions based on what other family members have done. |
| Information/help seeking | Accounts/descriptions of information or resources that participants would find useful |
| Parental views | Accounts/descriptions/thoughts/beliefs of parents in relation to their children. |

DRAFT
