# Supplementary material for: Enablers and barriers to treatment adherence in heterozygous familial hypercholesterolaemia: a qualitative evidence synthesis
Source: BMJ Open. 2019 Jul 31;9(7):e030290. doi: 10.1136/bmjopen-2019-030290 (PMC6677970; doi:10.1136/bmjopen-2019-030290)
Supplement: Author's manuscript [file bmjopen-2019-030290.draft_revisions.pdf]

BMJ Open is committed to open peer review. As part of this commitment we make the peer review history of every article we publish publicly available.

When an article is published we post the peer reviewers' comments and the authors' responses online. We also post the versions of the paper that were used during peer review. These are the versions that the peer review comments apply to.

The versions of the paper that follow are the versions that were submitted during the peer review process. They are not the versions of record or the final published versions. They should not be cited or distributed as the published version of this manuscript.

BMJ Open is an open access journal and the full, final, typeset and author-corrected version of record of the manuscript is available on our site with no access controls, subscription charges or pay-per-view fees (http://bmjopen.bmj.com).

If you have any questions on BMJ Open's open peer review process please email info.bmjopen@bmj.com

Enablers and barriers to treatment adherence in heterozygous familial hypercholesterolaemia: A qualitative evidence synthesis

Journal:	*BMJ Open*
Manuscript ID	bmjopen-2019-030290
Article Type:	Research
Date Submitted by the Author:	07-Mar-2019

Complete List of Authors:	Kinnear, Fiona; University of Bristol, NIHR Bristol Biomedical Research Centre (Nutrition theme)<br>Wainwright, Elaine; Bath Spa University, Psychology department<br>Perry, Rachel; University of Bristol, NIHR Bristol Biomedical Research Centre (Nutrition Theme)<br>Lithander, Fiona; University of Bristol, NIHR Bristol Biomedical Research Centre (Nutrition Theme)<br>Bayly, Graham; University Hospitals Bristol NHS Foundation Trust, Department of Clinical Biochemistry<br>Huntley, Alyson; University of Bristol, Bristol Medical School<br>Cox, Jennifer; University of Bristol, NIHR Bristol Biomedical Research Centre (Nutrition Theme)<br>Shield, Julian; University of Bristol, NIHR Bristol Biomedical Research Centre (Nutrition Theme)<br>Searle, Aidan; University of Bristol, NIHR Bristol Biomedical Research Centre (Nutrition Theme)

Keywords:	NUTRITION & DIETETICS, PREVENTIVE MEDICINE, QUALITATIVE RESEARCH, Coronary heart disease < CARDIOLOGY, Paediatric cardiology < CARDIOLOGY, Lipid disorders < DIABETES & ENDOCRINOLOGY

SCHOLARONE™
Manuscripts

Enablers and barriers to treatment adherence in heterozygous familial hypercholesterolaemia: A qualitative evidence synthesis

Fiona J Kinnear[1,2] Corresponding author, Elaine Wainwright[3], Rachel Perry[2], Fiona E Lithander[2], Graham Bayly[4], Alyson Huntley[5], Jennifer Cox[2], Julian P Hamilton-Shield[2], Aidan Searle[2].

1. Email Address: Fiona.kinnear@bristol.ac.uk; Telephone number: 0117 3421754
2. NIHR Bristol Biomedical Research Centre Nutrition Theme, University Hospitals Bristol NHS Foundation Trust and the University of Bristol, Bristol, UK
3. Psychology Department, Bath Spa University and Honorary Research Fellow, Department for Health, University of Bath, Bath, UK
4. Department of Clinical Biochemistry, University Hospitals Bristol NHS Foundation Trust, Bristol, UK

5. Population Health Science, Bristol Medical School, The University of Bristol, Bristol, UK

Word count excluding title page, abstract, references, figures and tables: 5700

For peer review only

ABSTRACT

Objectives Individuals with heterozygous familial hypercholesterolemia (FH) are at high risk of developing cardiovascular disease (CVD). This risk can be substantially reduced with lifelong pharmacological and lifestyle treatment however research suggests adherence is poor. This study aimed to synthesis research describing the experiences and beliefs of individuals with FH to identify enablers and barriers to treatment adherence.

Design A comprehensive search strategy was undertaken across MEDLINE, Embase, PsycINFO via OVID, Cochrane library and CINAHL databases and grey literature to identify all available qualitative research conducted in individuals with FH and their family members which collected data regarding their experiences of and beliefs about their condition and its treatment. Quality assessment was undertaken using the Critical Appraisal Skills Programme for qualitative studies. A thematic synthesis was conducted to uncover descriptive and generate analytical themes. These findings were then used to identify enablers and barriers to treatment adherence for application in clinical practice.

Results 24 papers reporting the findings of 15 population samples (246 individuals with FH and 13 of their family members) across seven countries were included. Data captured within 20 descriptive themes were considered in relation to treatment adherence and six analytical themes were generated: risk assessment; perceived personal control of health; disease identity; family influence; informed decision making; and incorporating treatment into daily life. These findings were used to identify seven enablers (e.g. 'commencement of treatment from a young age') and six barriers (e.g. 'incorrect and/or inadequate knowledge of treatment advice') to treatment adherence. There was insufficient data to explore if the findings differed between adults and children.

Conclusions The findings reveal several enablers and barriers to treatment adherence in individuals with FH. These could be utilised in clinical practice to facilitate optimal adherence to lifelong treatment thereby minimising the risk of CVD in this vulnerable population.

PROSPERO registration number CRD42018085946

For peer review only - http://bmjopen.bmj.com/site/about/guidelines.xhtml

Strengths and limitations of this study

- This is the first thematic synthesis of the qualitative literature exploring the beliefs and experiences of individuals with familial hypercholesterolaemia to identify enablers and barriers to treatment adherence that can be targeted in clinical practice
- Robust procedures for conducting a thematic synthesis were adopted, informed by the Cochrane Qualitative Research Methods Group guidelines and they were reported in line with the Enhancing Transparency in Reporting the Synthesis of Qualitative Research statement
- The barriers and enablers were identified from themes which were representative of all the included studies, increasing their validity
- While included studies were conducted across eight countries, all were within the developed world which could limit the generalisability of the findings

For peer review only - http://bmjopen.bmj.com/site/about/guidelines.xhtml

INTRODUCTION

Heterozygous familial hypercholesterolaemia FH is one of the most common inherited genetic disorders, estimated to affect as many as 1 in 250 individuals worldwide.[1, 2] Left untreated the exposure to chronically elevated levels of low density lipoprotein cholesterol LDL-C from birth confers an increased risk of cardiovascular disease CVD,[2, 3] with approximately 50% and 85% of affected women and men respectively experiencing a coronary event before the age of 65.[4] While this risk can be significantly reduced with early detection and treatment, many affected individuals remain at higher risk of premature CVD morbidity and mortality.[5-9] The most beneficial effects of treatment are evident in primary prevention before the onset of CVD.[5, 10] With diagnostic rates are as low as 1% in some countries,[11] current efforts are focussed on identifying individuals with FH via screening and genetic testing programs.[12, 13] Treated as outpatients and asked to follow lifelong treatment, it is critical to ensure that this increasing patient group are able to self-manage their disease. With many patients not reaching treatment targets[14-16] it is an area that warrants further investigation.

The current treatment recommended for individuals with FH is lipid lowering medication from the age of 8-10 years, alongside lifestyle advice to encourage engagement in physical activity PA, maintenance of a healthy weight, avoidance of smoking and consumption of a healthy diet.[17, 18] The available evidence suggests adherence to medication in adults may be sub-optimal,[16, 19-21] which may explain why target LDL-C levels are often not achieved.[14-16] Little is known about medication adherence rates in children, however many do not achieve the recommended[22] 50% reduction in LDL-C.[23, 24] Self-reported adherence to lifestyle advice is low in adults[25] and children[26, 27] which is substantiated by the presence of other CVD risk factors associated with unhealthy lifestyles in these groups such as hypertension and obesity.[16, 24, 28, 29] Adherence to lifestyle advice is essential for individuals with FH not only to decrease LDL-C levels, but to minimise their exposure to other risk factors which are independently associated with adverse CVD outcomes.[28-30]

To improve adherence to treatment recommendations, an understanding of the factors affecting adherence is required. The American Heart Association AHA has recognised the need to gain a deeper understanding of the experiences of individuals with FH before addressing the further identified research gaps.[31] Preliminary research has found the beliefs and attitudes of FH patients towards the recommended treatment exert a significant effect upon their intention to engage in these behaviours.[32, 33] Qualitative research can provide further insight to how these beliefs and attitudes are developed and the nature by which they may influence subsequent behaviours.[34] Its exploratory nature also allows for the identification of other factors influencing an individuals ability and motivation to comply with treatment.[35, 36]

Qualitative research conducted in FH patients has found illness knowledge[37], risk perception[38], a lack of symptoms[39] and family history of disease[40] to influence treatment adherence. However, the transferability of these findings beyond the sample they are conducted in is limited.[41] Qualitative syntheses, which bring

For peer review only - http://bmjopen.bmj.com/site/about/guidelines.xhtml

together the findings from individual qualitative studies, can be used to gain a more in depth understanding of the issue and identify common themes which are applicable to a wider range of contexts.[42, 43] It is recognised as an important source of evidence to inform healthcare interventions and policy development[44-46] including those targeting treatment adherence[47-49] and is advocated by the World Health Organisation and the Cochrane Collaboration Group.[42, 50]

Objectives

1. Identify how the experiences and beliefs of individuals with FH influence their adherence to pharmacological and lifestyle treatment recommendations

2. Explore if these findings differ between children and adults

3. Use the findings to generate new understanding of the enablers and barriers to treatment adherence which can be used to inform clinical practice

DRAFT
For peer review only

MATERIALS AND METHODS

The methods used for this qualitative synthesis are briefly described below with full details available in the published protocol[51] and on the PROSPERO database (registration number CRD42018085946). Minor deviations to the protocol were made, outlined in supplementary file 1. The Enhancing Transparency of Reporting the synthesis of Qualitative research (ENTREQ) statement[52] has been followed and a checklist is available in supplementary file 2.

Search strategy and selection criteria

A comprehensive, systematic and pre-planned search was conducted to find all available qualitative evidence exploring the experiences and beliefs of individuals, or their family members, with clinically diagnosed heterozygous FH in relation to their condition, its associated morbidity and mortality risk and recommended pharmacological and lifestyle change treatment. Full details are available in supplementary file 3.

Quality appraisal

The methodological quality of the studies was assessed using the Critical Appraisal Skills Program (CASP) tool for reviewing qualitative research.[53] As the purpose of the quality appraisal was to determine the methodological strengths and limitations of studies included in the synthesis, the lead authors of each paper were contacted to obtain further information in an attempt to overcome the recognised issued of poor reporting in qualitative research. Full details of how this tool was used are available in supplementary file 4.

Data extraction

Methodological and contextual information from each paper were extracted into a table designed for this review by two reviewers independently (FK, JC) after piloting in five papers. Two reviewers (FK, AS) independently reviewed all text under the results, conclusions and discussion headings of all papers, aswell as any supplementary files. Any data identified to be relevant to the research questions were extracted electronically using a tool designed for this review. In instances in which multiple papers reported the findings from a single study, the data from the primary paper PhD theses were extracted first, before supplementary publications were reviewed for any additional, unique data. Results were compared and discussed until agreement was reached.

Data analysis

Thematic synthesis[54], a widely accepted and commonly used approach in qualitative syntheses, was used to analyse the data.[55, 56] It involved three stages: line by line coding of the extracted data, generation of descriptive themes and development of analytical themes. Using Nvivo software, two reviewers (FK, AS) carried out the coding independently. The subsequent stages were carried out collaboratively between three

For peer review only - http://bmjopen.bmj.com/site/about/guidelines.xhtml

reviewers (FK, AS, EW). To enhance transparency, full details are available in supplementary file 5. The findings were discussed with three clinicians (JHS, GB, PD) currently providing care to individuals with FH to help develop feasible and relevant recommendations for clinical practice.

Sensitivity analysis

To ensure the quality appraisal results were used in a meaningful way,[55, 57] post-hoc sensitivity analysis was carried out to examine the extent to which the synthesis results were affected by exclusion of poor quality papers, described in full elsewhere.[58] It involved examining if any themes were lost when each paper was removed from synthesis and evaluate if there was a significant impact upon the 'thickness' of findings reported within each theme. 'Thickness' refers to the ability of the data to provide explanatory insights that can be generalised to the wider FH patient population.[59, 60] This was carried out through discussion between three reviewers (FK, AS, EW).

DRAFT For peer review only

RESULTS

Searches

Multiple papers reporting findings from the same sample of individuals and three PhD papers,[61-63] two of which had supplementary papers published in addition to the originally reported theses, were included. Each paper was considered to be a separate primary paper and referenced separately. Twenty-four papers were included in the synthesis, comprising of 18 original[37, 39, 61-76] and six supplementary papers[38, 40, 77-80] reporting the findings of 15 population samples (Figure 1).

Characteristics of studies and participants

In total, 264 individuals with FH and 13 family members were involved, aged 8-69 years. Seven papers[38, 39, 61, 73, 74, 77, 78] reported findings from three samples which included individuals under 18 years. Four papers reported parental views of having children with FH.[39, 71, 73, 74] The characteristics of the included papers and samples are presented in Table 1.

For peer review only - http://bmjopen.bmj.com/site/about/guidelines.xhtml

Table 1: Characteristics of included papers

Sample number	Author & date of paper	CASP quality rating[a, b]	Research aim	Country & recruitment setting	Sample Size[c]	Sample characteristics	Data collection methods
1	Agard et al, 2005[64]	Low	To explore the extent to which FH influences the life of the patients affected	Sweden<br>Outpatients treated at lipid clinic	23	10 M & 13 F; Mean age 48yrs (range: 31-67yrs); 4 with or had Hx of CVD	Face to face SSI
2	DeAngelis et al, 2017[65]	Low	To determine individual and group patient ideas and priorities regarding ways to enhance their own health	U.S.A.<br>Patients & family from patient centred outcomes research institute and outpatient clinic	7	6 FH patients, 1 family member	15 group meetings
3	Frich, 2007[61]	High*	To explore how individuals with FH perceive and manage their condition	Norway<br>Specialist clinic for metabolic lipid disorders	40	20 M & 20 F; Mean age 31yrs (range 14-57yrs); 7 had CVD symptoms; 19 had children	Face to face SSI
	Frich et al, 2006[77]	High*	To explore how patients with diagnosis of FH understand and perceive their vulnerability to CHD				
	Frich et al, 2007[78]	High*	To explore how patients at risk of CHD portray candidates for CHD				
	Frich et al, 2007[38]	High*	To explore patients' experiences of guilt and shame with regard to how they manage FH				
4	Hallowell et al, 2017[66]	High*	To investigate index patients' experiences of undergoing DNA testing as part of screening programme	Scotland<br>Two lipid clinics	38	17 M & 21 F; Mean age 52.6yrs (range 18-67yrs); 31 had children; 16 educated to university level	Face to face in depth interviews, 1 online
	Jenkins et al, 2013[68]	Medium*	To explore patient's interpretations of their DNA results for FH				
	Jenkins et al, 2013[67]	Low*	To explore the concept of inter-embodiment and its potential for advancing sociological research into illness biography and genetic identity				
5	Hardcastle et al, 2015[37]	High*	To investigate the perceptions and experiences of patients with a	Australia	18	10 M & 8 F; Mean age 50.2 yrs	Face to face SSI

For peer review only - http://bmjopen.bmj.com/site/about/guidelines.xhtml

			genetic diagnosis of FH involved in a cascade screening programme. To explore how these patients conceptualise FH and how such beliefs affect treatment compliance and lifestyle changes	Lipid disorders clinic		(range 25-74 yrs); 2 had CVD symptoms	
6	Hollands et al, 2012[69]	Low	Examine the impact of disease risk assessments based on both genetic and non-genetic information, or solely non-genetic information	U.K. Lipid clinics at 11 hospitals	20	12 M & 8 F; Mean age 30.9yrs for DNA diagnosed & 40.7yrs for non-DNA; 17 white, 1 white Asian, 2 black Caribbean	3 telephone interviews
7	Hollman et al, 2004[70]	High*	To describe the QOL and to understand the underlying meaning of the concept of QOL in patients with FH	Sweden Outpatient clinic	12	6 M & 6 F; 20-69yrs; 7 had children; 3 university level education; no Hx of CHV	Face to face SSI
8	Keenan et al, 2018[71]	Medium*	To explore parent's views and experiences of genetic testing and early treatment of children with FH in Scotland, experiences of their children's care pathway and to identify any barriers or facilitators in testing and treatment uptake	Scotland Clinical genetic services and lipid clinics from 3 sites	17	6 M & 11 F; 20-69yrs; all white; 12 had post-secondary qualifications; 3 symptoms or Hx of CVD	SSI 15 face to face, 2 over phone
9	Kirkegaard et al, 2014[72]	Medium*	Explore how cholesterol reducing medication and risk of CVD are interpreted by asymptomatic patients with high cholesterol	Denmark 5 GP centres.	3	1 M & 2 F; 24-62yrs; no CVD symptoms	Face to face SSI
10	Mackie et al, 2015[73]	High	Explore how family medical history, family narratives of medical experiences and AYA medical experiences together function as 'experiential evidence' and influence screening and treatment decisions	U.S.A Paediatric preventative cardiology practice	24	12 AYAs with FH and 12 parents of AYAs with FH 4 dyads<br><br>AYAs:6 M &, 6 F; Mean age 18.4yrs; 9 white, 1 black and 1 Asian<br>Parents: 2 M & 10 F; Mean age 49.3yrs; 1 Asian, 9 white	Face to face SSI with AYA and parent separately
	Sliwinski et al, 2017[39]	High	To examine challenges transitioning to adult care for young adults with FH, and their parents, in the context of 2 developmental tasks: transitioning from childhood to early adulthood and summing responsibility for self-management of a chronic disease				
11	Meulenkamp et al, 2008[74]	High*	To study the experiences of children identified by family screening who were found to be a mutation carrier for a genetic CVD	Netherlands Paediatric lipid clinic	16 childre	5 M & 11 F; 8-17yrs Number & age of parents not	Face to face SSI children and

					n from 10 families	given	parents separately
12	Mortensen et al, 2008[75]	Low	Comparative study to examine the QOL impact of FH in patients who had and had not reached the target of treatment	Denmark<br>Centre of inherited CVD	10	6 M & 4 F; 20-72yrs; no CVD Hx	Focus groups
13	Urke, 2016[62]	High	Explore how young adults, who stopped attending lipid clinic for medical and nutritional consultations, managed challenges related to living with FH and to the lifelong treatment	Norway<br>Outpatient clinic	11	6 M & 5 F; Median age 29yrs (range 26-35 yrs); 8 educated to university levels	SSI 9 face to face<br>2 over phone
14	Weiner, 2006[63]	High*	How much and in which way patients with FH and professionals involved with the condition construct FH and CHD as genetic conditions	England<br>Lipid clinic	31	17 M & 14 F; Mean age 52 yrs (range 24-69 yrs); 31 white; 15 with current CVD	Face to face SSI
	Weiner and Durrington, 2008[40]	Medium*	To explore patients' understanding and experiences of FH and the significance of the hereditary aspect of the condition				
	Weiner, 2009[79]	Medium*	Consider how people with FH construct FH, high cholesterol and CHD				
	Weiner, 2011[80]	Medium*	Explore the notion of genetic responsibility, focussing particularly on responsibilities to family and kin				
15	Senior et al, 2002[76]	Low	Investigate perceptions of having an inherited predisposition to heart disease in people diagnosed with, and receiving treatment for FH	England<br>2 lipid clinics	7	5 M & 2 F; 39-58 yrs	Face to face SSI

SSI= semi structured interview; M= male; F= female; CHD = coronary heart disease; CVD = cardiovascular disease; QOL = Quality of life; AYA= adolescent and young adult; Hx = history

[a] CASP score: high=18-20; medium=14-17; low quality=<14.

[b] Papers for which lead author provided requested further information are marked with *

[c] The sample size and characteristics describe only those in sample with clinically diagnosed heterozygous FH and their family members.

DRAFT
For peer review only

For peer review only - http://bmjopen.bmj.com/site/about/guidelines.xhtml

Quality Appraisal and Sensitivity Analysis

Appraisal scores of papers ranged from 11-20 out of 20, with eleven rated high, seven medium and six low Table 1. The most common methodological limitations uncovered were relating to ethical issues, researcher reflexivity and rigour of data analysis. Consideration of a researchers' potential influence and bias upon data collection and analysis was critically examined fully in seven papers,[38,39,61,62,73,77,78] partially in 10,[37,40,65,63,66,70,72,74,79,80] and not addressed in seven.[64,67,68,69,71,75,76] Ethical approval was obtained, or reasons given for exemption, in all but two papers,[75,76] however participants were not provided adequate information about withdrawal and anonymisation of data processes in a further 4 papers.[39,73,64,65] The data analysis was carried out by one researcher only in seven papers[37,71,40,62,63,79,80] and it was unclear if more than one person was involved at each stage of analysis in four papers.[75,76,66,67]

Eight lead authors responded to our request for further information, providing information for 16 of the 24 papers. Five of the six papers rated as low-quality were papers for which the author did not respond. This reflects our belief that low ratings may be reflective of poor reporting rather than poor methodology, supporting to our decision not to exclude papers. The sensitivity analysis carried out found that the removal of the five poor quality papers had no significant effect upon the synthesis findings- in both the descriptive and analytical themes uncovered and the depth of the findings. More detailed information of methodological and transferability issues is available in supplementary file 4.

Data analysis

Six analytical themes were derived from the findings captured by 20 identified descriptive themes, as displayed in Table 2 alongside illustrative quotes. Table 3 shows the occurrence of the descriptive themes within the extracted data from the 24 papers. While each analytical theme has a direct influence upon treatment adherence, they are not exclusive in nature and inter-theme relationships are evident as displayed in the thematic schema in Figure 2. Additionally, some themes by their integrative nature, had a greater influence upon treatment adherence as indicated by the shaded boxes. There were insufficient data regarding children and young people to explore whether the findings differed from adults.

Seven enablers and six barriers to treatment adherence (Table 4) were uncovered during the analysis of these themes and are described alongside the analytical themes below. In this section 'treatment' refers to both lifestyle and medication behaviours, unless otherwise specified.

Analytical themes

Risk assessment

Individuals lived experience of their disease, coupled with their beliefs concerning its known risks, increased or decreased their sense of vulnerability to its long-term health consequences. Knowledge of how FH had

For peer review only - http://bmjopen.bmj.com/site/about/guidelines.xhtml

affected family members was the most prevalent factor considered by individuals when assessing their risk. Individuals with lived experience of a family member being ill or dying prematurely due to FH, had a heightened sense of risk.[59, 61, 62, 65, 68, 69, 71-75] Individuals unaware of FH in their families or with family members living a life unaffected by its consequences, perceived themselves at lower risk:[59, 65, 69, 71, 74, 75] 'My dad's now in his 70s…it's not something I feel particularly threatened about having.'[69]

As FH does not 'make you feel ill' [65], individuals found having FH 'easy to forget, and easy not to take seriously.'[60] This was salient amongst younger individuals without existing CVD symptoms [36, 38, 60, 61, 71, 72, 78] for whom '…cholesterol always comes last. It will never be a focus until something happens to me.'[60] Older individuals who had lived through, or were currently experiencing CVD, perceived themselves at higher risk.[36, 69, 74, 75] Others framed their perception of risk in the context of the risk they believed other diseases presented, concluding that FH health consequences were not as serious:[36, 60, 61, 64, 66, 67, 74]'I didn't think it was life threatening, like being told you've got cancer.'[36]

For the majority of individuals, their risk assessment led to a perception that FH did not present a great risk to their current or long-term health.[36, 60-62, 64, 69, 72-74] This mismatch between the perceived and actual risk has been identified as a barrier to treatment adherence.

Perceived personal control of health

Individuals acknowledged the threat that FH posed to their health, but there was a widely held belief that they had the ability to modify their own personal risk.[36-38, 60-64, 66, 67, 69-72, 74, 75] They recognised that this required active engagement with treatment[36-38, 60, 62-64, 66-69, 71, 74, 75] and held themselves accountable for managing their disease[36-38, 60-64, 66-71, 73-75] experiencing a 'bad conscience'[62] and 'guilt'[76] when they did not meet the expectations they had set themselves. Treatment was perceived to be effective[37, 60, 62, 64, 66-75] with individuals viewing FH as 'treatable'[61] and 'controllable'.[36] In particular, medication was regarded by individuals to be a mandatory and effective component of treatment.[37, 60, 62, 64, 66-75] They believed FH could be 'solved'[72] with medication and lead to achievement of cholesterol levels 'like most people.'[36] While individuals spoke of their efforts to change their lifestyle behaviours,[37, 38, 60, 62, 64, 66-75] many believed their cholesterol levels would not be 'radically changed'[74] by doing so[60, 61, 71, 73] as it 'doesn't matter what I eat or how much exercise I'm still going to have high cholesterol without tablets.'[36]

This confidence in the ability to successfully self-manage their condition was identified as an enabler to treatment adherence. The perceived effectiveness of medication led to a devaluing of the importance of following lifestyle treatment[36, 60, 61, 70, 71, 73] and this prioritisation of medication was identified as a barrier to adhering to lifestyle treatment.

For peer review only - http://bmjopen.bmj.com/site/about/guidelines.xhtml

Disease identity

Individuals placed great importance, especially in social situations, to emphasis that they were 'not to blame'[73] for their high cholesterol.[37, 39, 61, 63, 64, 66, 67, 70, 73, 74, 76] High cholesterol was associated with unhealthy lifestyles and individuals wished to distance themselves from this negative connotation.[37, 61, 67, 70, 73, 74, 76] A positive genetic test provided 'a definitive'[64], rather than a possible, explanation for their high cholesterol[63, 66, 67] and positively influenced individuals perceptions and behaviours.[63, 64, 66, 67, 69] If individuals had been following treatment of their volition before the diagnosis, it helped 'reaffirm their commitment'[66] to treatment.[64, 67] If they had been previously unaware of their condition it prompted them to seek treatment:[66, 69] 'I know now and can take preventative measures.'[67]

Therefore, receiving a formal diagnosis was identified as an enabler to treatment adherence as being given a medical explanation empowered individuals to take control of their condition through engaging with treatment.

Family influence

Parents expressed a high level of concern about the well-being of their affected children[38, 61, 63, 64, 66, 69, 71, 72] and this parental responsibility to care for children was identified as another enabler of treatment adherence. They assumed responsibility to ensure their children adhered to medical and lifestyle treatment,[38, 61, 63, 64, 66, 69, 71, 72] taking action to 'bring them up with healthy eating habits'[64] and 'make sure that they take their medication.'[61] This involvement was reflected in the finding of individuals attributing their current treatment knowledge and behaviours to their parents:[60-62] 'everything I've learned from home.'[60] Parents also made treatment-related decisions on their behalf [38, 61, 63, 66, 71, 72] until they were 'old enough to decide.'[69] As such, the early adulthood years presented a challenge for treatment adherence as the young adults transitioned from being under the care of their parents to assuming responsibility for their behaviours.[38, 60]

Growing up surrounded by family members following treatment recommendations and establishing healthy behaviours from a young age was found to instil lifelong habits in individuals.[38, 61, 69, 71, 72] Those who had grown up from a young age alongside diagnosed family members spoke of their condition and its treatment as something that had become 'normalised'[60] as it was all they had ever known.[38, 61, 69, 71, 72] Those who had parents who had bad experiences of medication were apprehensive about taking tablets,[71] but for many it led to the view that taking medication was ordinary[69] and not a 'big deal.'[71] Two enablers to treatment adherence were identified from these findings: commencement of treatment from a young age and having other family members following similar treatment regimes.

For peer review only - http://bmjopen.bmj.com/site/about/guidelines.xhtml

Informed decision making

Individuals lacked an in-depth understanding of their disease and its treatment,[36-38, 60-64, 69-72, 74] with many having 'unanswered questions'[62] and requesting more information.[38, 62-64] Misconceptions and false information regarding the role of treatment for FH were prevalent:[37, 38, 60-62, 64, 69-72, 74] 'you can actually eat a lot of fat and the medicine takes care of it.'[36] Individuals were worried about the longer-term impact of statin therapy on their, and their children's, health[62, 71] as 'it is a recent drug, and you don't know what the long term effect could be.'[69] Lived experience of side effects were reported by some individuals[62, 71, 73] and many more were fearful of developing them in the future[68, 69, 71] as 'many others have severe side effects from what i'm taking.'[73] This incorrect and/or inadequate knowledge of treatment advice and concerns over the short- and long-term use of lipid lowering medication were identified as barriers to treatment adherence.

Individuals frequently mentioned their encounters with healthcare professionals HCPs[36, 37, 59-61, 63, 65, 66, 69, 70, 72, 73],viewing them as playing a 'big role'[38] in their 'team approach'[71] to the management of their FH. Regardless of whether individuals recalled these encounters in a positive[37, 38, 60, 61, 63, 69, 71] or negative[37, 59, 60, 69, 73] light, these interactions and relationships with HCPs influenced their understanding of FH and its treatment. Therefore, a positive relationship with HCPs was identified as an enabler to treatment adherence.

Integrating treatment into daily life

Individuals did not feel they had to make many changes to their everyday life as a result of their diagnosis.[36, 60-62, 64, 67, 74] Their disease did not them from 'living the life they wanted'[60] or require consideration when making life decisions[36, 60, 62, 67, 74] such as having children.[61, 64] However, when faced with other commitments, such as family and career obligations, individuals found it more difficult.[36, 38, 60, 62, 67, 73, 75] During these periods individuals tended to be less focused on managing their disease viewing it as something they could pick up again when they had more time and energy.[36, 38, 60, 69, 75] This prioritisation of other life events over the self-management of condition was identified as a barrier to treatment adherence.

The treatment recommendations were perceived to be simple to follow and to have little impact on their QOL.[36, 60-62, 64, 66-69, 74] However, this perception is in stark contrast to the actual lived experiences of following treatment- especially the lifestyle recommendations. Dietary advice was perceived to be restrictive and interpreted by individuals to mean they could not eat their favourite foods[37, 38, 60, 61, 70, 72] or enjoy social occasions:[37, 38, 67, 70, 72, 73]'I won't bother eating food I don't like, just to follow a certain diet.'[60] Additionally, patients were concerned about the opinions of their peers in social situations in which they felt they had to make certain dietary choices.[38, 60, 61, 72, 73] These findings were prominent amongst younger individuals.[38, 60, 72] As a result, the dietary advice was the 'most difficult aspect'[62] of treatment, with many reporting they struggled to follow them at all times.[36-38, 60, 61, 70, 72, 73] This finding of dietary advice being perceived as difficult to follow was identified as a barrier to adherence.

For peer review only - http://bmjopen.bmj.com/site/about/guidelines.xhtml

Reflective of the difficulties faced when trying to follow treatment guidelines, individuals expressed a need for additional information[36, 62, 63, 69] and ‘guidelines in order to help you start that change.’[38] Some sought additional information from their HCPs,[36, 38, 62, 63, 69] while others called for practical advice and educational resources,[38, 62, 63, 69] as ‘everyone knows the theory, but putting it into practice is quite hard.’[36] From this, practical resources and support for following lifestyle treatment advice was identified as an enabler to treatment adherence.

DRAFT For peer review only

Table 2: Analytical themes and their composite descriptive themes with illustrative quotes

Analytical theme	Descriptive themes	Illustrative quotes from participants (1st order)	Illustrative interpretations from authors (2nd order)
**Risk assessment**	FH is a silent disease	'not a condition that has any symptoms, that makes you feel ill or anything.'[67]	'The majority of interviewees did not look upon the condition as a disease…If they were not affected by a cardiac disease…they regarded themselves as healthy.'[64]
	Family history modifies perception of FH related threat to health	'I'm not going to get past sixty. Dad never got past sixty.'[68]	'To them, reaching the age of death of a parent with FH was anticipated with fear of having a heart attack themselves.'[75]
	FH is not as threatening to health as other conditions	'Its not that bad...Its not like having something like Huntingdon's or something like that.'[66]	They mentioned conditions with more drastic consequences such as allergies, epilepsy or diabetes.'[62]
**Perceived personal control of health**	FH is a manageable condition	'well it's treatable isn't it by diet and drugs. It's not something that's incurable.'[63]	'FH carrier children demonstrated high feelings of control over their condition.'[74]
	Individuals feel personally responsible for managing their FH	'it means you could be in danger of like what could possibly happen like in the future if you don't change anything.'[73]	'FH patients have a strong desire to empower themselves in order to improve their own health.'[65]
	FH medication is effective	'I believe that as I am taking the pills that my risk of heart attack is no greater than anyone else of my age or weight.'[76]	'Preventative medical treatment built confidence in the potential for living a long life.'[70]
	FH lifestyle treatment viewed as less important than medication	'I could never get that down no matter 'ow much dieting or exercise I do…so it can only be reduced through medication.'[63]	'Many tended to devalue the importance of lifestyle changes in controlling FH and place their hope in medication.'[37]
**Disease identity**	Importance of establishing that high cholesterol levels are not self-inflicted	'It enables me to emphasise that it is not my fault, that it's something inherited.'[77]	'they always described FH as a hereditary condition to underline that their cholesterol issues were not due to unhealthy lifestyle.'[75]
	Receiving genetic diagnosis provides certainty	'I guess it is a relief in a funny way because I had an answer to what was quite a surprising medical condition that I had…so at least I know now and can take preventative measures.'[69]	it provided an aetiological explanation and diagnostic label, confirmed current risk management practices…'[38]
**The influence of family**	Desire to protect children	'we want to help him…so we have decided to give him statins until he is 16…we've covered him until he's old enough to decide for himself.'[71]	'In fact, the main concern for the affected parents appeared to be the well-being of their children…'[64]
	Parental influence upon treatment related behaviours	'my parents, specifically my mom, were really integral in teaching us types of food to eat..'[39]	'AYAs expressed how their perceptions of their parents experience have influenced their perceptions of the respective treatment options.'[73]
	FH and its treatment become normalised within families	'Since I grew up with FH and had a relatively good diet and good habits and routines, it makes it easier.'[62]	'FH carrier children typically reported it had become habit to maintain a healthy, non-fat diet. Commonly the whole family, including the non-carriers, kept to the same diet restrictions.'[74]
**Infor med**	HCP interactions	'My daughter. I don't think she really understood what high cholesterol really meant until she came here and talked with doctor.'[73]	'The doctors presentation of FH, however, influenced all patients perceptions of the risk and severity of the diagnosis.'[75]

DRAFT
For peer review only

**decision making**	Inadequate and/or incorrect knowledge about FH & treatment	'in the newspapers, the stories that you cut out butter, red meat, etc., and you'll be okay.'[76]	'Many informants still had unanswered questions or were felt to lack relevant knowledge.'[64]
	Concerns about side effects of FH medication	'would I be able to have children at all after taking all these medicines for years?'[64]	'Parents reported having strong concerns about statin treatment in children, not only because of their long-term safety but also potential side effects.'[71]
**Incorporating treatment into daily life**	FH and it's treatment does not have big impact upon life	'You don't have to plan your life around it. You don't have to wonder, can you have children or not.'[66]	'FH was not viewed as a significant burden, but more of a lifestyle adjustment, involving a healthy diet, exercise, and statin treatment from an early age.'[71]
	Balancing FH treatment with other competing priorities	'Our two children, who were often ill…My husband…travelled all the time, so I almost had more than I could put up with at that moment.'[77]	'Young adults also articulated challenges maintaining diet and exercise regimes while adjusting to a new routine and environment at college or in workforce.'[39]
	Lifestyle advice treatment is restrictive and difficult to follow	'I've changed my diet as much as I can… don't want to bother too much and speculate, live an unworthy life and diet at the age of seventy. I'd rather be happy and die when I'm fifty.'[38]	'Making dietary changes had been the worst aspect of their condition, and this included people who already had CHD.'[77]
	Social implications of following FH treatment	'Some people comment on the things I eat. And then I'm like 'well actually I have to eat this because I've got FH and I have to watch my diet.'[69]	'10 young adults articulated how concern over peers' opinions or overt peer pressure-restricted social activities centered around eating.'[39]
	Desire for further support and guidance	I think having the resources would make it easy to adhere to lifestyle treatment…like seeing a nutritionist that can give you options...'[39]	'..expressed a desire to be able to access educational resources in one place and for a way to reach out to others who could provide solidarity, comfort and aid with management of FH.'[65]

AYA= adolescent and young adult; HCP = healthcare professional; CHD = cardiovascular heart disease

For peer review only - http://bmjopen.bmj.com/site/about/guidelines.xhtml

Table 3: Occurrence of descriptive themes across the included papers and samples [a]

		Descriptive themes																			
Sample number	Paper	FH is a silent disease	Family history modifies perception of FH related threat to health	FH is not as threatening to health as other conditions	FH is a manageable condition	Individuals feel personally responsible for managing their FH	FH medication is effective	FH lifestyle treatment viewed as less important that medication	Importance of establishing that high cholesterol levels are not self-inflicted	Receiving genetic diagnosis provides certainty	Desire to protect children	Parental influence upon treatment related behaviours	FH and its treatment become normalised within families	HCP relationships	Inadequate and/or incorrect knowledge about FH and its treatment	Concerns about side effects of FH medication	FH and its treatment does not have big impact upon life	Balancing FH treatment with other competing priorities	Lifestyle advice treatment is restrictive and difficult to follow	Social implications of following FH treatment	Desire for further support and guidance
1	Agard et al, 2005[64]	✓	✓		✓	✓	✓				✓	✓		✓	✓	✓	✓	✓	✓	✓	✓
2	DeAngelis et al, 2017[65]					✓				✓	✓			✓	✓						✓
3	Frich, 2007[61]		✓											✓	✓						
	Frich et al, 2006[77]		✓		✓	✓	✓											✓			
	Frich et al, 2007[78]								✓						✓						
	Frich et al, 2007[38]				✓	✓			✓	✓				✓	✓				✓	✓	
4	Hallowell et al, 2017[66]			✓	✓	✓	✓		✓	✓	✓	✓			✓		✓				✓
	Jenkins et al, 2013[68]			✓	✓	✓	✓		✓	✓	✓			✓			✓				
	Jenkins et al, 2013[67]	✓	✓											✓							
5	Hardcastle et al, 2015[37]	✓	✓	✓	✓	✓	✓	✓			✓			✓	✓		✓	✓	✓		✓
6	Hollands et al, 2012[69]					✓			✓	✓							✓			✓	
7	Hollman et al, 2004[70]		✓		✓	✓	✓									✓					
8	Keenan et al, 2018[71]		✓		✓	✓	✓				✓	✓		✓	✓	✓		✓	✓		✓
9	Kirkegaard et al, 2014[72]		✓			✓	✓	✓	✓					✓	✓				✓	✓	
10	Mackie et al, 2015[73]	✓	✓		✓	✓	✓	✓			✓	✓	✓	✓	✓	✓					
	Sliwinski et al, 2017[39]	✓				✓					✓	✓	✓	✓	✓			✓	✓	✓	✓
11	Meulenkamp et al, 2008[74]	✓	✓		✓	✓	✓				✓	✓	✓	✓	✓		✓		✓	✓	
12	Mortensen et al, 2008[75]				✓	✓	✓	✓	✓		✓			✓		✓		✓		✓	
13	Urke, 2016[62]	✓		✓	✓	✓	✓	✓				✓	✓	✓	✓		✓	✓	✓	✓	
14	Weiner, 2006[63]	✓	✓	✓	✓	✓	✓	✓	✓		✓	✓	✓	✓	✓		✓	✓	✓		
	Weiner and Durrington, 2008[40]								✓												
	Weiner, 2009[79]								✓												
	Weiner, 2011[80]	✓																			
15	Senior et al, 2002[76]		✓	✓	✓	✓	✓		✓						✓		✓				

HCP = healthcare professional

[a] Themes identified within supplementary papers were only documented if they were evident in extracted data not reported in the primary paper and vice versa.

For peer review only - http://bmjopen.bmj.com/site/about/guidelines.xhtml

Table 4: Identified enablers and barriers to treatment adherence

Enablers	Barriers
Other family members following treatment regime	Mismatch between perceived and actual risk
Commencement of treatment from a young age	Concerns over the use of lipid lowering medication
Parental responsibility to care for children	Prioritisation of medication over lifestyle treatment
Confidence in ability to successfully self-manage their condition	Lifestyle treatment is difficult to comply with
Receiving formal diagnosis of FH	Prioritisation of other life events
Practical resources & support for following lifestyle treatment	Inadequate and/or incorrect knowledge of treatment advice
A positive relationship with healthcare professional	

DRAFT
For peer review only

For peer review only - http://bmjopen.bmj.com/site/about/guidelines.xhtml

DISCUSSION

This synthesis has produced new insights into the factors influencing treatment adherence in individuals with FH. The findings are discussed in relation to the available literature within the FH population and implications for clinical practice and future research.

We found that for the majority, FH is not perceived as threatening to health. This is in accordance with findings from a cross-sectional questionnaire study in which adults felt at low risk of developing CVD.[25] We also found that individuals with symptoms of CVD or a family history of FH related CVD felt at greater risk of experiencing FH associated adverse outcomes, in line with the findings of several other questionnaire studies.[25, 81, 82] This low perception of risk may be the result of the disease being relatively symptomless and the adverse consequences too far in the future to comprehend. This idea is reinforced by studies reporting heightened perceived risk amongst older individuals[83] and over 90% of young adults perceiving their health to be average or above that of the general population.[16] Similarly, a systematic review of qualitative studies, which aimed to explore reasons behind the low diagnostic rate of FH, reported that the significance an individual placed on their diagnosis was decreased in those who were younger, without a family history of CVD and no current symptoms.[84] This low perception of significance decreased the likelihood of an individual informing their relatives about their genetic risk of having FH, which is likely mediated by a low sense of risk to their, and their family members, health.

The minimal threat to health likely contributes to the findings that being diagnosed with FH does not increase the occurrence of psychosocial dysfunction in children,[27, 85] nor negatively impact upon self-reported quality of life QOL in adults.[86-88] While worry and anxiety amongst individuals have been reported in two small samples,[16, 83] a systematic review found individuals with FH to exhibit lower symptoms of anxiety and mental health related QOL relative to general population controls, and similar levels of physical health related QOL and depression.[89] While these findings are positive, individuals who do not view their disease as a serious threat may be less motivated to adhere to treatment. This hypothesis is substantiated by findings of higher self-reported medication adherence in older individuals[21, 90] and individuals under 36 years being ten times more likely to be non-adherent with medication.[91]

These findings are concerning as individuals who do not adhere fully to treatment will remain at a high risk of CVD. This is demonstrated by lower LDL-C achieved by individuals with higher rates of self-reported medication adherence.[21, 91, 92] Furthermore, while treatment has substantially reduced the risk of CVD individuals still remain at a higher risk than the general population.[9, 93, 94] This may be a consequence of LDL-C targets not being met by large numbers of treated adults[15, 16, 91, 92, 95] and children[96, 97] and/or the presence of other risk factors independently associated with CVD.[29, 30] It appears that many individuals with FH are not cognisant of the true risks associated with their disease.

For peer review only - http://bmjopen.bmj.com/site/about/guidelines.xhtml

However, our findings suggest this low perceived threat to health may be mediated by beliefs that the risks are avoidable through active engagement with effective treatment. This is in line with findings from self-report questionnaires showing that individuals with FH believe strongly in the efficacy of lifestyle and medication treatment.[16, 25, 27, 98] These beliefs have in turn been found to be positively associated with rates of medication adherence.[99] Furthermore, in a study conducted across seven countries, treatment control beliefs influenced attitudes toward medication, which in turn increased intention to take medication.[33]

In disagreement with these findings, individuals attitudes toward treatment behaviours have been found to influence their intention to engage in healthy eating, PA and medication recommendations, as opposed to illness or treatment efficacy beliefs.[32] We found individuals to have negative attitudes toward the dietary recommendations, perceiving them as restrictive and impacting upon their QOL, in line with other studies investigating child and parental concerns.[27, 100] Some individuals also believed they were unnecessary if taking medication, which may explain findings of low uptake of lifestyle treatment compared to medication.[25, 26] We also found negative attitudes towards medication due to side effects experienced and anxieties about long-term safety, similar to findings of studies investigating adherence.[16, 20, 95] However in contrast to these studies, we found anxiety about the development of side effects and complications of long-term use was more influential upon adherence than lived experience of side-effects. Our findings of negative attitudes towards medication and lifestyle advice are surprising as the dietary recommendations do not differ substantially from those for the general population and the safety and tolerability of statins have been demonstrated in adults[101] and children.[102-104] Our further finding of widespread inadequate knowledge of the treatment recommendations may serve as an explanation.

Given our finding that individuals decisions about treatment are informed by their medication concerns and treatment knowledge it is important to understand the content and context in which this information is being communicated and interpreted. We found many individuals had a poor understanding of the dietary and PA guidelines, smoking and weight management were infrequently mentioned. Awareness of the role of PA in treatment has been found to be low[19] and while individuals are mindful of the need for dietary treatment,[19, 100] little is known about the depth of this knowledge. Findings of studies investigating the care provided to individuals with FH report that not all are receiving the recommended lifestyle advice[26, 105, 106] or medication treatment.[26, 95, 97, 105, 107, 108] Furthermore, these may not be provided by HCPs with the required specialist knowledge of FH.[26, 106] This inconsistency in care provided may explain the finding of this synthesis, and other studies,[19, 26] that many individuals would like more information about their treatment. Even if individuals do receive care in line with treatment guidelines, they are still exposed to media coverage of research which they may falsely interpret to be relevant to their condition. Concerns about general medication overuse have been found to be more influential than side effect beliefs in shaping attitudes toward FH medication[33] which is concerning as statin medications are portrayed negatively in the media[109]

For peer review only - http://bmjopen.bmj.com/site/about/guidelines.xhtml

and many individuals with FH search for information on the internet.[26] Ensuring individuals have a comprehensive and factually correct understanding of the treatment recommendations is essential to optimise adherence.

We have found this to be particularly important for parents who, in addition to making treatment decisions for their affected children, influence their ability to adhere to treatment in later life through their involvement in the establishment of lifelong habits. A study conducted in FH children reported that those who were too young to remember being diagnosed, did not perceive the dietary guidelines as restrictive as it was all they had ever known.[85] The dietary intakes of children with FH who have received nutrition counselling have been found to be more in line with treatment recommendations than age-matched controls,[110-112] and the dietary habits are maintained into young adulthood[113] highlighting the importance of providing this advice at a young age. Forgetfulness is frequently reported as a reason for medication non-adherence[16, 20, 21, 27, 90, 92] and starting treatment at a young age may help overcome this by instilling a routine, as found in a small study of children with FH.[114] As we have found parents take responsibility for their child's care, it is important to ensure that when patients reach an age where they become responsible for their own care, they are equipped with the relevant knowledge to continue to make informed decisions. There was insufficient data from young people with FH included in the synthesis to draw conclusions about best practice for this age group. However, it appears that transitioning from living at home, adjusting to new routines and prioritising other things in life may present a challenge to adhering to treatment in late adolescence and early adulthood.[39, 62] These reasons were suggested to explain the low recruitment rate 57% of individuals 10 years after they had taken part in a childhood statin trial.[16] The authors also reported not being able to reach 33% of the individuals highlighting further difficulties of maintaining contact with patients when they leave home.

Our findings also highlight the importance of receiving a genetic confirmation of FH. Receiving a medical diagnosis empowered individuals to take control of their condition, providing motivation to continue or commence medication and lifestyle treatments. Others have reported genetic diagnosis to be associated with an increased perception of medication efficacy[82, 115] and adherence.[81] However, in contrast to our findings it has been reported that positive genetic results have either no effect[82] or weaken beliefs[115] regarding the efficacy of lifestyle treatment. However, in both cases the changes in beliefs did not have a negative impact upon their actual behaviours. Given our further finding that individuals find medical diagnosis useful in social situations, a common identified barrier to adhering to dietary recommendations, it may be that genetic diagnosis exerts positive effect upon adherence beyond its influence of illness and treatment beliefs.

Strengths and limitations

Our thematic synthesis adhered to ENTREQ guidelines and used transparent and robust methodology. The comprehensive search strategy, involvement of more than one researcher at each stage of analysis, input

For peer review only - http://bmjopen.bmj.com/site/about/guidelines.xhtml

from clinicians to corroborate the interpretation of the results, detailed appraisal of the included studies and post hoc sensitivity analysis all strengthen our findings. The analytical themes generated were produced from descriptive themes that were each evident across a large number of the included papers. The synthesis included data from 264 individuals with FH and 13 family members across eight countries, encompassing a wide range of ages, duration of diagnoses, primary and secondary CVD prevention and regional differences in healthcare provision. However, all individuals were from developed countries, the majority had high education levels and there were few from ethnic minority groups. This may limit the generalisability of the findings to all individuals with FH. Furthermore, the vast majority of individuals were recruited from lipid clinics and therefore already self-selected to receive treatment. Their beliefs may not reflect those opting out of treatment for their condition, an area we have highlighted as warranting future research. Lastly, there were insufficient papers to explore if the factors influencing treatment adherence differ between adults and children with FH and care should be taken when extrapolating results to younger individuals. However, we were able to produce clinical implications for children based on adult recollections of childhood experiences.

Implications for clinical practice

We have identified seven enablers and six barriers to treatment adherence Table 4 which could be acknowledged and incorporated by any HCP delivering personalised medication or lifestyle advice to individuals with FH.

Given the improved prognosis for individuals with FH and the increasingly young age at which individuals are being diagnosed, the gap between perceived and actual risk of CVD will continue to grow. HCPs should ensure individuals are aware of the risk to their health, without instilling fear. This could be achieved by emphasising the effectiveness of medical and lifestyle treatment in controlling this risk. It should be highlighted to younger individuals and parents that despite the asymptomatic nature of the condition, adhering to treatment from a young age will deliver the greatest benefits to their long-term health.

Discussion about medication should be delivered within an FH context, emphasising the requirement, and efficacy, of pharmacological treatment for their inherited condition and distinguishing this from treatment of other causes of high cholesterol. Individuals should be reassured that medication is safe and side effects uncommon, with reference to relevant clinical guidelines indicating their safety for use by children highlighted to parents. Individuals should also be informed that side effects are specific to each type of medication and encouraged to discuss any problems at appointments so that alterative medications can be offered. When discussing lifestyle treatment emphasis should be placed on the similarity of the recommendations to those advised to the general population. It should be communicated as a lifestyle change rather than a restrictive diet with advice tailored to the individual needs and preferences of each individual and/or family. HCPs should also ensure that patients have a factually correct understanding of the

For peer review only - http://bmjopen.bmj.com/site/about/guidelines.xhtml

dietary recommendations and provide credible resources individuals can access if they require further support or guidance. The benefits of adhering to lifestyle treatment should be revisited frequently with individuals with the benefits for both management of their disease and their overall well-being emphasised.

Children should be encouraged to start treatment from a young age to instil healthy habits, with parents advised that prior to medication, dietary recommendations can be followed from the age of five. Non-affected family members can also be encouraged to follow guidelines, facilitating a family-based approach to aid adherence. FH is an autosomal dominant condition, meaning that except for the rare instance of a *de novo* mutation, all affected individuals will have one affected parent. Therefore, ideally treatment advice should be provided in family-based clinic with both parent and children receiving advice together. If this is not possible then it would be beneficial to ensure adult and paediatric services are closely linked. Adolescent patients should be offered opportunity to transition to an adult clinic between the ages of 16-18 so they can start to take responsibility for their own treatment before they leave home.

Future research

With the optimal risk reduction evident in those starting treatment at a young age,[6, 10, 97] and our findings of the positive effects of this upon later life adherence, children and young adults stand to benefit the most from adhering to treatment. Further qualitative research exploring the perspectives of this age group is required to determine if differences exist in this age group compared to adults to allow HCPs to tailor advice to support maximal adherence.

The findings of widespread inadequate and/or incorrect knowledge and understanding of the treatment recommendations warrants investigation into what individuals are being told, and by whom. We recognise that individuals may be acquiring information from sources outside the clinic in which they receive care and further research to explore their understanding of the treatment recommendations would be beneficial to identify any common misconceptions or knowledge gaps which could be addressed by HCPs. We also identified a lack of data regarding the current lifestyle habits of individuals with FH. Obtaining this information would allow for comparison between the actual and recommended lifestyle habits which could identify areas for HCPs to prioritise when delivering advice. This would also provide baseline data to allow investigation into the potential additive of lifestyle factors in addition to medication upon LDL-C, for which it is recognised there is insufficient evidence.[103, 116]

As individuals who have already self-selected to attend clinic and receive treatment have concerns about the safety and necessity of medication for FH treatment, it is likely that there are many individuals with FH opting not to receive treatment for themselves or their child due to these concerns. Future research is needed to explore the perceptions of these individuals to develop effective interventions that could change these beliefs and encourage them to seek treatment.

For peer review only - http://bmjopen.bmj.com/site/about/guidelines.xhtml

Conclusions

This qualitative evidence synthesis has systematically reviewed and synthesised the available evidence concerning the experiences and beliefs of individuals with FH regarding their condition and its treatment. It has uncovered several enablers and barriers that are to be utilised in clinical practice to facilitate optimal treatment adherence in this high-risk clinical population group. It has also highlighted significant research gaps which need to be addressed to gain a more comprehensive understanding of how these individuals can be supported to adhere to lifelong treatment.

DRAFT
For peer review only

Acknowledgements

We would like to thank Catherine Borwick (Research Engagement Librarian) and Dr. Alison Gregory (Research Fellow) at The University of Bristol for their expertise and assistance in the development of this evidence synthesis. We would also like to thank Dr. Paul Downie (Consultant at University Hospitals Bristol and Royal United Hospitals Bath NHS Foundation trusts) for his input into translating the findings into clinical recommendations.

Author contributions: FK and RP devised and carried out the search strategy. FK, RP, FL and JHS carried out the study screening and selection stage. FK and JC carried out the study characteristic extraction stage. FK and AS carried out the results data extraction, quality appraisal, data analysis and interpretation. EW also carried out the data analysis and interpretation stages. AH contributed to the development and presentation of the qualitative methodology and results. FK, JHS and GB translated findings into clinical implications. FK prepared the manuscript. All authors reviewed the manuscript and approved the final version.

Funding: This evidence synthesis is funded by the National Institute for Health Research NIHR Bristol Biomedical Research Centre (Nutrition theme) at University Hospitals Bristol NHS Foundation Trust and The University of Bristol.

Disclaimer: The views expressed in this publication are those of the authors and not necessarily those of the NHS, the NIHR or the Department of Health.

Competing interests: None declared.

Abbreviations:

FH: Familial Hypercholesteroleamia

LDL-C: Low density lipoprotein cholesterol

CVD: Cardiovascular disease

CASP: Critical apprisal skills programme

PA: Physical activity

AHA: The American Heart Association

ENTREQ: Enhancing transparency in reporting the synthesis of qualitative research

CHD: Coronary heart disease

SSI: Semi-structured interview

AYA: Adult and young adolescent

HCPs: Healthcare professionals

QOL: Quality of life

For peer review only - http://bmjopen.bmj.com/site/about/guidelines.xhtml

References

1. Akioyamen LE, Genest J, Shan SD, Reel RL, Albaum JM, Chu A, et al. Estimating the prevalence of heterozygous familial hypercholesterolaemia: a systematic review and meta-analysis. BMJ Open. 2017;79.
2. Benn M, Watts GF, Tybjærg-Hansen A, Nordestgaard BG. Mutations causative of familial hypercholesterolaemia: screening of 98 098 individuals from the Copenhagen General Population Study estimated a prevalence of 1 in 217. European heart journal. 2016;3717:1384-94.
3. Wong B, Kruse G, Kutikova L, Ray KK, Mata P, Bruckert E. Cardiovascular Disease Risk Associated With Familial Hypercholesterolemia: A Systematic Review of the Literature. Clinical therapeutics. 2016;387:1696-709.
4. Civeira F. Guidelines for the diagnosis and management of heterozygous familial hypercholesterolemia. Atherosclerosis. 2004;1731:55-68.
5. Humphries SE, Cooper JA, Seed M, Capps N, Durrington PN, Jones B, et al. Coronary heart disease mortality in treated familial hypercholesterolaemia: Update of the UK Simon Broome FH register. Atherosclerosis. 2018;274:41-6.
6. Mohrschladt MF, Westendorp RG, Gevers Leuven JA, Smelt AH. Cardiovascular disease and mortality in statin-treated patients with familial hypercholesterolemia. Atherosclerosis. 2004;1722:329-35.
7. Versmissen J, Oosterveer DM, Yazdanpanah M, Defesche JC, Basart DCG, Liem AH, et al. Efficacy of statins in familial hypercholesterolaemia: a long term cohort study. BMJ. 2008;337.
8. Besseling J, Hovingh GK, Huijgen R, Kastelein JJP, Hutten BA. Statins in Familial Hypercholesterolemia: Consequences for Coronary Artery Disease and All-Cause Mortality. Journal of the American College of Cardiology. 2016;683:252-60.
9. Mundal LJ, Igland J, Veierod MB, Holven KB, Ose L, Selmer RM, et al. Impact of age on excess risk of coronary heart disease in patients with familial hypercholesterolaemia. Heart British Cardiac Society. 2018.
10. Neil A, Cooper J, Betteridge J, Capps N, McDowell I, Durrington P, et al. Reductions in all-cause, cancer, and coronary mortality in statin-treated patients with heterozygous familial hypercholesterolaemia: a prospective registry study. European heart journal. 2008;2921:2625-33.
11. Nordestgaard BG, Chapman MJ, Humphries SE, Ginsberg HN, Masana L, Descamps OS, et al. Familial hypercholesterolaemia is underdiagnosed and undertreated in the general population: guidance for clinicians to prevent coronary heart disease : Consensus Statement of the European Atherosclerosis Society. European heart journal. 2013;3445:3478-90.
12. Kerr M, Pears R, Miedzybrodzka Z, Haralambos K, Cather M, Watson M, et al. Cost effectiveness of cascade testing for familial hypercholesterolaemia, based on data from familial hypercholesterolaemia services in the UK. European heart journal. 2017;3823:1832-9.
13. Louter L, Defesche J, Roeters van Lennep J. Cascade screening for familial hypercholesterolemia: Practical consequences. Atherosclerosis Supplements. 2017;30:77-85.
14. Hartgers ML, Besseling J, Stroes ES, Wittekoek J, Rutten JHW, de Graaf J, et al. Achieved LDL cholesterol levels in patients with heterozygous familial hypercholesterolemia: A model that explores the efficacy of conventional and novel lipid-lowering therapy. J Clin Lipidol. 2018.
15. Pijlman AH, Huijgen R, Verhagen SN, Imholz BPM, Liem AH, Kastelein JJP, et al. Evaluation of cholesterol lowering treatment of patients with familial hypercholesterolemia: a large cross-sectional study in The Netherlands. Atherosclerosis. 2010;2091:189-94.
16. Langslet G, Bogsrud MP, Halvorsen I, Fjeldstad H, Retterstøl K, Veierød MB, et al. Long-term follow-up of young adults with familial hypercholesterolemia after participation in clinical trials during childhood. Journal of Clinical Lipidology. 2015;96:778-85.
17. NICE clinical guideline 71: Familial hypercholesterolemia: identification and management. Internet. 2008 cited 19/01/2018. Available from: http://nice.org.uk/guidance/cg71.
18. Catapano AL, Graham I, De Backer G, Wiklund O, Chapman MJ, Drexel H, et al. 2016 ESC/EAS Guidelines for the Management of Dyslipidaemias. European heart journal. 2016;3739:2999-3058.
19. Hollman G, Olsson AG, Ek AC. Disease knowledge and adherence to treatment in patients with familial hypercholesterolemia. The Journal of cardiovascular nursing. 2006;212:103-8.
20. Benson G, Witt DR, VanWormer JJ, Campbell SM, Sillah A, Hayes SN, et al. Medication adherence, cascade screening, and lifestyle patterns among women with hypercholesterolemia: Results from the WomenHeart survey. J Clin Lipidol. 2016;104:937-43.

For peer review only - http://bmjopen.bmj.com/site/about/guidelines.xhtml

21. Senior V, Marteau TM, Weinman J. Self-Reported Adherence to Cholesterol-Lowering Medication in Patients with Familial Hypercholesterolaemia: The Role of Illness Perceptions. Cardiovascular Drugs and Therapy. 2004;186:475-81.

22. Wiegman A, Gidding SS, Watts GF, Chapman MJ, Ginsberg HN, Cuchel M, et al. Familial hypercholesterolaemia in children and adolescents: gaining decades of life by optimizing detection and treatment. European heart journal. 2015;3636:2425-37.

23. Pang J, Martin AC, Bates TR, Hooper AJ, Bell DA, Burnett JR, et al. Parent-child genetic testing for familial hypercholesterolaemia in an Australian context. J Paediatr Child Health. 2018.

24. Humphries SE, Cooper J, Dale P, Ramaswami U. The UK Paediatric Familial Hypercholesterolaemia Register: Statin-related safety and 1-year growth data. Journal of Clinical Lipidology. 2017.

25. Claassen L, Henneman L, Kindt I, Marteau TM, Timmermans DRM. Perceived Risk and Representations of Cardiovascular Disease and Preventive Behaviour in People Diagnosed with Familial Hypercholesterolemia: A Cross-sectional Questionnaire Study. Journal of Health Psychology. 2010;151:33-43.

26. Avis HJ, Kusters DM, Vissers MN, Huijgen R, Janssen TH, Wiegman A, et al. Follow-Up of Children Diagnosed with Familial Hypercholesterolemia in a National Genetic Screening Program. The Journal of Pediatrics. 2012;1611:99-103.

27. de Jongh S, Kerckhoffs MC, Grootenhuis MA, Bakker HD, Heymans HSA, Last BF. Quality of life, anxiety and concerns among statin-treated children with familial hypercholesterolaemia and their parents. Acta Pædiatrica. 2003;929:1096-101.

28. Besseling J, Kindt I, Hof M, Kastelein JJP, Hutten BA, Hovingh GK. Severe heterozygous familial hypercholesterolemia and risk for cardiovascular disease: A study of a cohort of 14,000 mutation carriers. Atherosclerosis. 2014;2331:219-23.

29. Galema-Boers AM, Lenzen MJ, Engelkes SR, Sijbrands EJ, Roeters van Lennep JE. Cardiovascular risk in patients with familial hypercholesterolemia using optimal lipid-lowering therapy. Journal of Clinical Lipidology. 2018;122:409-16.

30. Pérez de Isla L, Alonso R, Mata N, Fernández-Pérez C, Muñiz O, Díaz-Díaz JL, et al. Predicting Cardiovascular Events in Familial Hypercholesterolemia. Circulation. 2017;13522:2133.

31. Gidding SS, Champagne MA, de Ferranti SD, Defesche J, Ito MK, Knowles JW, et al. The Agenda for Familial Hypercholesterolemia: A Scientific Statement From the American Heart Association. Circulation. 2015;13222:2167-92.

32. Hagger MS, Hardcastle SJ, Hingley C, Strickland E, Pang J, Watts GF. Predicting Self-Management Behaviors in Familial Hypercholesterolemia Using an Integrated Theoretical Model: the Impact of Beliefs About Illnesses and Beliefs About Behaviors. International Journal of Behavioral Medicine. 2016;233:282-94.

33. Hagger MS, Hardcastle SJ, Hu M, Kwok S, Lin J, Nawawi HM, et al. Effects of medication, treatment, and behavioral beliefs on intentions to take medication in patients with familial hypercholesterolemia. Atherosclerosis. 2018;277:493-501.

34. Green J, Britten N. Qualitative research and evidence based medicine. BMJ : British Medical Journal. 1998;3167139:1230-2.

35. George M, Freedman TG, Norfleet AL, Feldman HI, Apter AJ. Qualitative research-enhanced understanding of patients' beliefs: Results of focus groups with low-income, urban, African American adults with asthma. Journal of Allergy and Clinical Immunology. 2003;1115:967-73.

36. Remien RH, Hirky AE, Johnson MO, Weinhardt LS, Whittier D, Le GM. Adherence to Medication Treatment: A Qualitative Study of Facilitators and Barriers Among a Diverse Sample of HIV+ Men and Women in Four U.S. Cities. AIDS and Behavior. 2003;71:61-72.

37. Hardcastle SJ, Legge E, Laundy CS, Egan SJ, French R, Watts GF, et al. Patients’ Perceptions and Experiences of Familial Hypercholesterolemia, Cascade Genetic Screening and Treatment. International Journal of Behavioral Medicine. 2015;221:92-100.

38. Frich JC, Malterud K, Fugelli, Frich JC, Malterud K, Fugelli P. Experiences of guilt and shame in patients with familial hypercholesterolemia: a qualitative interview study. Patient Education & Counseling. 2007;691-3:108-13.

39. Sliwinski SK, Gooding H, de Ferranti S, Mackie TI, Shah S, Saunders T, et al. Transitioning from pediatric to adult health care with familial hypercholesterolemia: Listening to young adult and parent voices. Journal of Clinical Lipidology. 2017;111:147-59.

40. Weiner K, Durrington PN. Patients' understandings and experiences of familial hypercholesterolemia. Community genetics. 2008;115:273-82.

For peer review only - http://bmjopen.bmj.com/site/about/guidelines.xhtml

41. Finfgeld-Connett D. Generalizability and transferability of meta-synthesis research findings. Journal of Advanced Nursing. 2010;662:246-54.
42. Harris JL, Booth A, Cargo M, Hannes K, Harden A, Flemming K, et al. Cochrane Qualitative and Implementation Methods Group guidance series—paper 2: methods for question formulation, searching, and protocol development for qualitative evidence synthesis. Journal of Clinical Epidemiology. 2018;97:39-48.
43. Sheldon TA. Making evidence synthesis more useful for management and policy-making. Journal of health services research & policy. 2005;101_suppl:1-5.
44. Ring NA, Ritchie K, Mandava L, Jepson R. A guide to synthesising qualitative research for researchers undertaking health technology assessments and systematic reviews. 2011.
45. Wells M, Williams B, Firnigl D, Lang H, Coyle J, Kroll T, et al. Supporting 'work-related goals' rather than 'return to work' after cancer? A systematic review and meta-synthesis of 25 qualitative studies. Psycho-oncology. 2013;226:1208-19.
46. Thomas J, Sutcliffe K, Harden A, Oakley A, Oliver S, Rees R, et al. Children and Healthy Eating: A Systematic Review of Barriers and Facilitators2003.
47. Mechta Nielsen T, Frojk Juhl M, Feldt-Rasmussen B, Thomsen T. Adherence to medication in patients with chronic kidney disease: a systematic review of qualitative research. Clinical kidney journal. 2018;114:513-27.
48. Ma Q, Tso LS, Rich ZC, Hall BJ, Beanland R, Li H, et al. Barriers and facilitators of interventions for improving antiretroviral therapy adherence: a systematic review of global qualitative evidence. Journal of the International AIDS Society. 2016;191:21166.
49. Munro SA, Lewin SA, Smith HJ, Engel ME, Fretheim A, Volmink J. Patient adherence to tuberculosis treatment: a systematic review of qualitative research. PLoS medicine. 2007;47:e238.
50. Langlois EV, Tuncalp, O., Norris, S.L., Askew, I., and Ghaffar, A. . Qualitative evidence to improve guidelines and health decision-making. Bulletin of the World Health Organization. 2018 9679-79A.
51. Kinnear FJ, Perry R, Searle A, Hamilton-Shield JP, Lithander FE. How do the experiences and beliefs of adults and children with heterozygous familial hypercholesterolaemia influence their adherence to treatment? A systematic review of qualitative evidence protocol. Syst Rev. 2018;71:120.
52. Tong A, Flemming K, McInnes E, Oliver S, Craig J. Enhancing transparency in reporting the synthesis of qualitative research: ENTREQ. BMC medical research methodology. 2012;121:181.
53. CASP Qualitative Research Checklist Internet. 2017 cited 19/01/2018. Available from: http://docs.wixstatic.com/ugd/dded87_25658615020e427da194a325e7773d42.pdf
54. Thomas J, Harden A. Methods for the thematic synthesis of qualitative research in systematic reviews. BMC medical research methodology. 2008;81:45.
55. Noyes J, Booth A, Flemming K, Garside R, Harden A, Lewin S, et al. Cochrane Qualitative and Implementation Methods Group guidance series-paper 3: methods for assessing methodological limitations, data extraction and synthesis, and confidence in synthesized qualitative findings. J Clin Epidemiol. 2018;97:49-58.
56. Barnett-Page E, Thomas J. Methods for the synthesis of qualitative research: a critical review. BMC medical research methodology. 2009;9:59.
57. Carroll C, Booth A. Quality assessment of qualitative evidence for systematic review and synthesis: Is it meaningful, and if so, how should it be performed? Research Synthesis Methods. 2014;62:149-54.
58. Carroll C, Booth A, Lloyd-Jones M. Should we exclude inadequately reported studies from qualitative systematic reviews? An evaluation of sensitivity analyses in two case study reviews. Qualitative health research. 2012;2210:1425-34.
59. Noyes J, Popay J. Directly observed therapy and tuberculosis: how can a systematic review of qualitative research contribute to improving services? A qualitative meta-synthesis. Journal of Advanced Nursing. 2007;573:227-43.
60. Popay J, Rogers A, Williams G. Rationale and standards for the systematic review of qualitative literature in health services research. Qualitative health research. 1998;83:341-51.
61. Frich JC. Living with genetic risk of coronary heart disease: A qualitative interview study of patients with familial hypercholesterolemia. Thesis 2007.
62. Urke EB. Young adults with familial hypercholesterolemia and their thoughts on lifelong treatment. A qualitative interview study. . Master Thesis 2016.
63. Weiner K. Patient and Professional COnstructions of Familial Hypercholesterolaemia and Heart Disease: Testing the Limits of the Geneticisation Thesis. . PhD Thesis 2006.

For peer review only - http://bmjopen.bmj.com/site/about/guidelines.xhtml

64. Agard A, Bolmsjo IA, Hermeren G, Wahlstom J. Familial hypercholesterolemia: ethical, practical and psychological problems from the perspective of patients. Patient Educ Couns. 2005;572:162-7.
65. DeAngelis EJ, McIntosh S, Ahmed CD, Block RC. Familial hypercholesterolaemia patient–determined themes for community-engaged research. Health Education Journal. 2017;773:293-302.
66. Hallowell N, Jenkins N, Douglas M, Walker S, Finnie R, Porteous M, et al. A qualitative study of patients' perceptions of the value of molecular diagnosis for familial hypercholesterolemia FH. Journal of Community Genetics. 2017;81:45-52.
67. Jenkins N, Lawton J, Douglas M, Hallowell N. Inter-embodiment and the experience of genetic testing for familial hypercholesterolaemia. Sociology of Health and Illness. 2013;354:529-43.
68. Jenkins N, Lawton J, Douglas M, Walker S, Finnie R, Porteous M, et al. How do index patients participating in genetic screening programmes for familial hypercholesterolemia FH interpret their DNA results? A UK-based qualitative interview study. Patient Education and Counseling. 2013;903:372-7.
69. Hollands GJ, Armstrong D, Macfarlane A, Crook MA, Marteau TM. Patient accounts of diagnostic testing for familial hypercholesterolaemia: comparing responses to genetic and non-genetic testing methods. BMC Medical Genetics. 2012;13:87-.
70. Hollman G, Ek A, Olsson AG, Berterö C. The meaning of quality of life among patients with familial hypercholesterolemia. Journal of Cardiovascular Nursing. 2004;194:243-50.
71. Keenan KF, Finnie RM, Simpson WG, McKee L, Dean J, Miedzybrodzka Z. Parents' views of genetic testing and treatment of familial hypercholesterolemia in children: a qualitative study. Journal of Community Genetics. 2018.
72. Kirkegaard P, Edwards A, Risor MB, Thomsen JL. Risk of cardiovascular disease? A qualitative study of risk interpretation among patients with high cholesterol. BMC family practice. 2013;14:137.
73. Mackie TI, Tse LL, de Ferranti SD, Ryan HR, Leslie LK. Treatment decision making for adolescents with familial hypercholesterolemia: Role of family history and past experiences. Journal of Clinical Lipidology. 2015;94:583-93.e3.
74. Meulenkamp TM, Tibben A, Mollema ED, Van Langen IM, Wiegman A, De Wert GM, et al. Predictive genetic testing for cardiovascular diseases: Impact on carrier children. American Journal of Medical Genetics, Part A. 2008;14624:3136-46.
75. Mortensen GL, Madsen IB, Kruse C, Bundgaard H. Familial hypercholesterolaemia reduces the quality of life of patients not reaching treatment targets. Danish medical journal. 2016;635.
76. Senior V, Smith JA, Michie S, Marteau TM. Making sense of risk: An interpretive phenomenological analysis of vulnerability to heart disease. Special Issue: Interpretive phenomenological analysis and the new genetics. 2002;72:157-68.
77. Frich JC, Ose L, Malterud K, Fugelli P. Perceived vulnerability to heart disease in patients with familial hypercholesterolemia: a qualitative interview study. Annals of family medicine. 2006;43:198-204.
78. Frich JC, Malterud K, Fugelli P. How do patients at risk portray candidates for coronary heart disease? A qualitative interview study. Scandinavian Journal of Primary Health Care. 2007;252:112-6.
79. Weiner K. The tenacity of the coronary candidate: how people with familial hypercholesterolaemia construct raised cholesterol and coronary heart disease. Health London, England : 1997. 2009;134:407-27.
80. Weiner K. Exploring genetic responsibility for the self, family and kin in the case of hereditary raised cholesterol. Social Science & Medicine. 2011;7211:1760-7.
81. van Maarle MC, Stouthard ME, Bonsel GJ. Risk perception of participants in a family-based genetic screening program on familial hypercholesterolemia. American journal of medical genetics Part A. 2003;116a2:136-43.
82. Claassen L, Henneman L, van der Weijden T, Marteau TM, Timmermans DRM. Being at risk for cardiovascular disease: Perceptions and preventive behavior in people with and without a known genetic predisposition. Psychology, Health & Medicine. 2012;175:511-21.
83. Andersen LK, Jensen HK, Juul S, Faergeman O. Patients' attitudes toward detection of heterozygous familial hypercholesterolemia. Archives of internal medicine. 1997;1575:553-60.
84. Muir LA, George PM, Whitehead L. Using the experiences of people with familial hypercholesterolaemia to help reduce the risk of cardiovascular disease: a qualitative systematic review. Journal of Advanced Nursing. 2012;689:1920-32.
85. Tonstad S, Novik TS, Vandvik IH. Psychosocial function during treatment for familial hypercholesterolemia. Pediatrics. 1996;982 Pt 1:249-55.
86. van Maarle MC, Stouthard ME, Bonsel GJ. Quality of life in a family based genetic cascade screening programme for familial hypercholesterolaemia: a longitudinal study among participants. Journal of medical genetics. 2003;401:e3.

For peer review only - http://bmjopen.bmj.com/site/about/guidelines.xhtml

87. van Maarle MC, Stouthard ME, Marang-van de Mheen PJ, Klazinga NS, Bonsel GJ. How disturbing is it to be approached for a genetic cascade screening programme for familial hypercholesterolaemia? Psychological impact and screenees' views. Community genetics. 2001;44:244-52.
88. Hyttinen L, Kekäläinen P, Vuorio AF, Sintonen H, Strandberg TE. Health-related quality of life in elderly patients with familial hypercholesterolemia. International Journal of Technology Assessment in Health Care. 2008;242:228-34.
89. Akioyamen LE, Genest J, Shan SD, Inibhunu H, Chu A, Tu JV. Anxiety, depression, and health-related quality of life in heterozygous familial hypercholesterolemia: A systematic review and meta-analysis. Journal of psychosomatic research. 2018;109:32-43.
90. Galema-Boers A, Langendonk JG, Roeters Van Lennep J, Lenzen MJ, Van Domburg RT, Sijbrands EJG. Developing a prediction model to identify noncompliance in a nurse-led familial hypercholesterolemia clinic. European Heart Journal. 2011;1:66.
91. Galema-Boers JM, Lenzen MJ, van Domburg RT, Roeters van Lennep J, van Bruchem-van de Scheur GG, Sijbrands EJ, et al. Predicting non-adherence in patients with familial hypercholesterolemia. European journal of clinical pharmacology. 2014;704:391-7.
92. Braamskamp MJAM, Kusters DM, Avis HJ, Smets EMA, Wijburg FA, Kastelein JJP, et al. Long-Term Statin Treatment in Children with Familial Hypercholesterolemia: More Insight into Tolerability and Adherence. Paediatric Drugs. 2015;172:159-66.
93. Retterstol K, Mundal L, Igland J, Seppola Tell G, Holven K, Braglien Veierød M, et al. Incidence of various types of atherosclerotic disease in patients with genotyped familial hypercholesterolemia. Atherosclerosis. 2017;263:e26.
94. Hopkins PN. Putting Into Perspective the Hazards of Untreated Familial Hypercholesterolemia. Journal of the American Heart Association. 2017;66.
95. deGoma EM, Ahmad ZS, O'Brien EC, Kindt I, Shrader P, Newman CB, et al. Treatment Gaps in Adults With Heterozygous Familial Hypercholesterolemia in the United States: Data From the CASCADE-FH Registry. Circulation Cardiovascular genetics. 2016;93:240-9.
96. Saltijeral A, Perez de Isla L, Alonso R, Muniz O, Diaz-Diaz JL, Fuentes F, et al. Attainment of LDL Cholesterol Treatment Goals in Children and Adolescents With Familial Hypercholesterolemia. The SAFEHEART Follow-up Registry. Revista espanola de cardiologia English ed. 2017;706:444-50.
97. Humphries SE, Cooper J, Dale P, Ramaswami U. The UK Paediatric Familial Hypercholesterolaemia Register: Statin-related safety and 1-year growth data. J Clin Lipidol. 2018;121:25-32.
98. Senior V, Marteau T, Weinman J. Perceptions of control over heart disease in people with an inherited predisposition to raised cholesterol. Psychology, Health & Medicine. 2005;101:16-30.
99. Senior V, Marteau TM. Causal attributions for raised cholesterol and perceptions of effective risk-reduction: Self-regulation strategies for an increased risk of coronary heart disease. Psychology and Health. 2007;226:699-717.
100. Tonstad S. Familial hypercholesterolaemia: a pilot study of parents' and children's concerns. Acta paediatrica Oslo, Norway : 1992. 1996;8511:1307-13.
101. Collins R, Reith C, Emberson J, Armitage J, Baigent C, Blackwell L, et al. Interpretation of the evidence for the efficacy and safety of statin therapy. The Lancet. 2016;38810059:2532-61.
102. Vuorio A, Kuoppala J, Kovanen PT, Humphries SE, Tonstad S, Wiegman A, et al. Statins for children with familial hypercholesterolemia. Cochrane Database Syst Rev. 2017;7:Cd006401.
103. Lozano P, Henrikson NB, Morrison CC, Dunn J, Nguyen M, Blasi PR, et al. Lipid Screening in Childhood and Adolescence for Detection of Multifactorial Dyslipidemia: Evidence Report and Systematic Review for the US Preventive Services Task Force. Jama. 2016;3166:634-44.
104. Radaelli G, Sausen G, Cesa CC, Santos FS, Portal VL, Neyeloff JL, et al. Statin Treatments And Dosages In Children With Familial Hypercholesterolemia: Meta-Analysis. Arquivos brasileiros de cardiologia. 2018.
105. van Maarle MC, Stouthard MEA, de Mheen PJM-v, Klazinga NS, Bonsel GJ. Follow up after a family based genetic screening programme for familial hypercholesterolaemia: is screening alone enough? BMJ : British Medical Journal. 2002;3247350:1367-8.
106. Kinnear FJ, Lithander FE, Hamilton-Shield JP, Main L. What dietetic support is available to patients in outpatient lipid clinics in the uk? Atherosclerosis Supplements. 2018;34:e5.
107. Umans-Eckenhausen MA, Defesche JC, van Dam MJ, Kastelein JJ. Long-term compliance with lipid-lowering medication after genetic screening for familial hypercholesterolemia. Archives of internal medicine. 2003;1631:65-8.

For peer review only - http://bmjopen.bmj.com/site/about/guidelines.xhtml

108. Jones LK, Kulchak Rahm A, Manickam K, Butry L, Lazzeri A, Corcoran T, et al. Healthcare Utilization and Patients' Perspectives After Receiving a Positive Genetic Test for Familial Hypercholesterolemia. Circulation Genomic and precision medicine. 2018;118:e002146.
109. Chisnell J, Marshall T, Hyde C, Zhelev Z, Fleming LE. A content analysis of the representation of statins in the British newsprint media. BMJ Open. 2017;78.
110. Tonstad S, Sivertsen M. Food dislikes and dietary intake in children with familial hypercholesterolemia and controls. Scandinavian Journal of Nutrition/Naringsforskning. 1996;404:146-50.
111. Stender S, Skovby F, Haraldsdottir J, Andresen GR, Michaelsen KF, Neilsen BS, et al. Cholesterol-lowering diets may increase the food costs for Danish children. A cross-sectional study of food costs for Danish children with and without familial hypercholesterolaemia. European Journal of Clinical Nutrition. 1993;4711:776-86.
112. Dixon LB, Tershakovec AM, McKenzie J, Shannon B. Diet quality of young children who received nutrition education promoting lower dietary fat. Public health nutrition. 2000;34:411-6.
113. Molven I, Retterstol K, Andersen LF, Veierod MB, Narverud I, Ose L, et al. Children and young adults with familial hypercholesterolaemia FH have healthier food choices particularly with respect to dietary fat sources compared with non-FH children. Journal of nutritional science. 2013;2:e32.
114. West RJ. FAMILIAL HYPERCHOLESTEROLAEMIA. FACTORS INFLUENCING DRUG COMPLIANCE. Pediatric Research. 1980;14:1423.
115. Marteau T, Senior V, Humphries SE, Bobrow M, Cranston T, Crook MA, et al. Psychological impact of genetic testing for familial hypercholesterolemia within a previously aware population: a randomized controlled trial. American journal of medical genetics Part A. 2004;128a3:285-93.
116. Malhotra A, Shafiq N, Arora A, Singh M, Kumar R, Malhotra S. Dietary interventions plant sterols, stanols, omega-3 fatty acids, soy protein and dietary fibers for familial hypercholesterolaemia. Cochrane Database Syst Rev. 20146:CD001918.

For peer review only - http://bmjopen.bmj.com/site/about/guidelines.xhtml

Figure 1: PRISMA Flow diagram

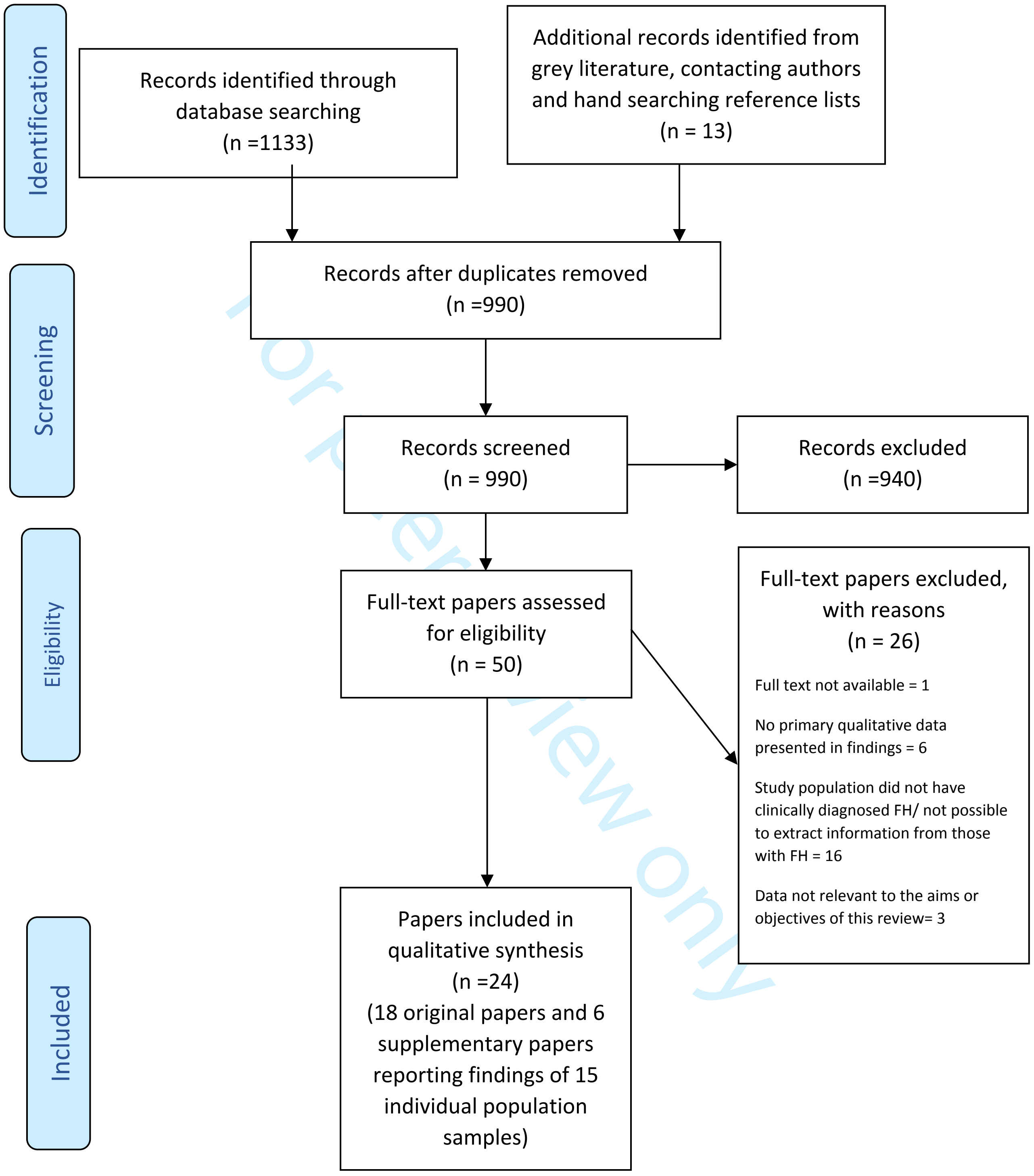

Figure 2: Thematic schema illustrating influence of analytical themes upon treatment adherence

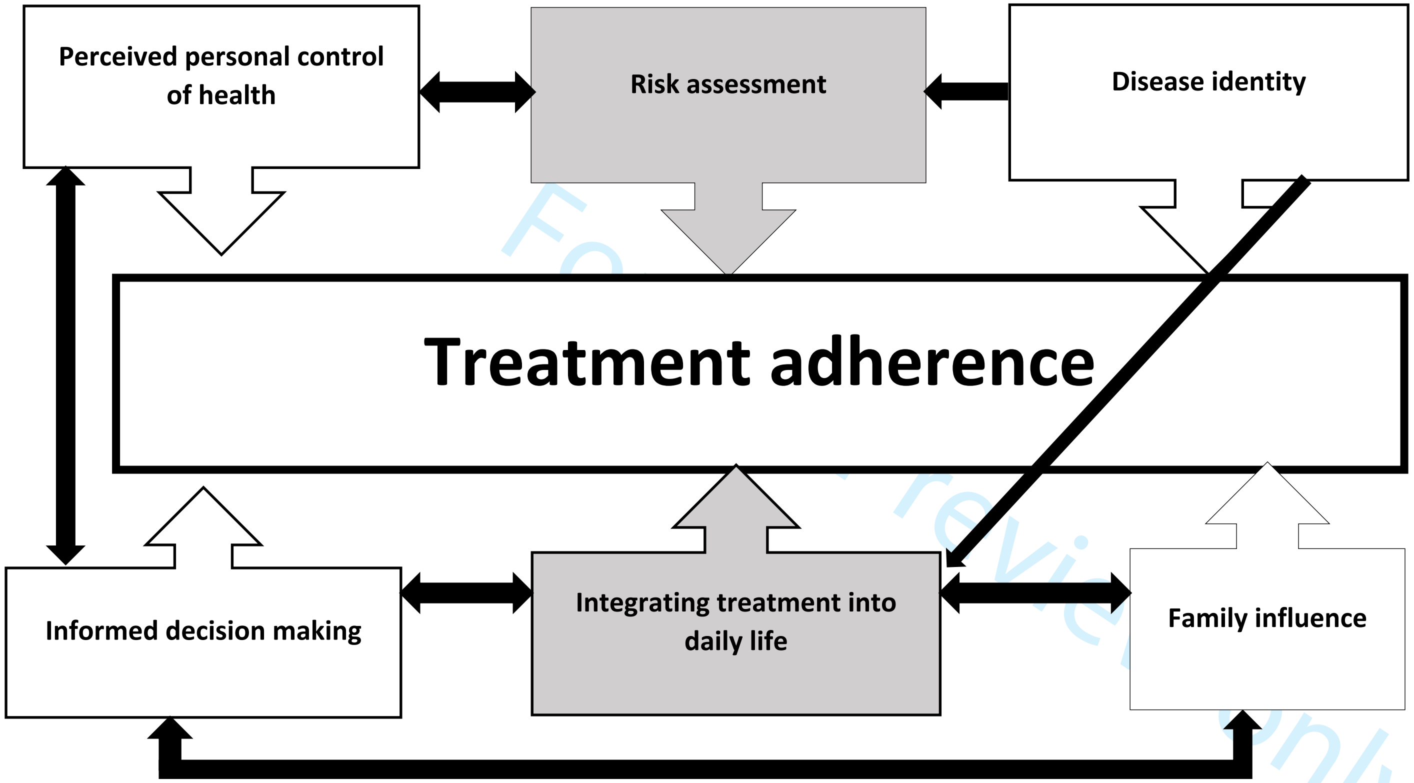

For peer review only

Supplementary File 1: Deviations from protocol

Stated in protocol	What we did	Rationale for deviation
'Only studies in which the full text is available in English will be eligible for inclusion'	We did not place any limits upon language of included papers	We aimed to overcome the recognised restrictions of individual qualitative study findings, by gathering and examining a wide range of patient perceptions and experiences. After an initial scope of the available evidence base in this population group, it was apparent that the number of potential papers to be retrieved would be manageable by the research team. Therefore, the decision was made to remove this exclusion criteria, in order to identify all relevant evidence in line with the comprehensive searching approach to be taken in this review. This is in line with available guidance which advises that language filter decisions should be made in reference to the aims of the review.(42,118,119)
'The participants include individuals aged ≥ 10 years'	We did not place any limits upon age of included participants.	The database searching retrieved a paper reporting findings from a sample which included children aged 8 years. As it was not possible to extract the data from only participants aged 10 years and older, using the original inclusion criteria the paper would have to be excluded from the synthesis. This paper was one of only 3 papers retrieved that reported findings from samples including children, therefore the findings were perceived to be very valuable to the synthesis. Children are often diagnosed with FH before the age of 10, and U.K. and international guidance advise treatment with lifestyle advice, with lipid lowering therapy to be implemented when they reach a suitable age.(17,18,120) Furthermore, it is stated in the NICE guidelines that lipid lowering drug treatment should be commenced by the age of 10 and statin therapy can be considered at 8-10 years of age.(17)
'Both stages of data extraction will be carried out independently by two reviewers (AS, FK)…'	First stage of data extraction (study details) was carried out by two reviewers (JC, FK) and second stage (study findings) by two reviewers (AS, FK).	This was to split work between review members.
'The two reviewers (FK, AS) will then work in collaboration to develop initial descriptive themes and categories based upon the raw data…'	Three reviewers (FK, AS, EW) worked in collaboration for the second two stages of thematic synthesis.	EW joined review team after publication of protocol. We felt having a further expert opinion from a health psychologist would improve the synthesis output.
'The findings are intended to be used in the development of future intervention or guidelines….'	The findings are presented with a focus on informing clinical practice	The findings were interpreted to be of particular importance to clinical practice. While the findings are still useful to intervention and guideline development, this paper will focus upon their application in a clinical setting,

For peer review only - http://bmjopen.bmj.com/site/about/guidelines.xhtml

Supplementary File 2- ENTREQ reporting guidelines checklist

ENTREQ: Enhancing transparency in reporting the synthesis of qualitative research [52]

No	Item	Guide and description	Reported on page #
**1**	Aim	State the research question the synthesis addresses.	Page 4.
**2**	Synthesis methodology	Identify the synthesis methodology or theoretical framework which underpins the synthesis, and describe the rationale for choice of methodology *(e.g. meta-ethnography, thematic synthesis, critical interpretive synthesis, grounded theory synthesis, realist synthesis, meta-aggregation, meta-study, framework synthesis).*	Protocol and page 5.
**3**	Approach to searching	Indicate whether the search was pre-planned (*comprehensive search strategies to seek all available studies)* or iterative (*to seek all available concepts until they theoretical saturation is achieved)*.	Protocol, page 5 and supplementary file 3.
**4**	Inclusion criteria	Specify the inclusion/exclusion criteria *(e.g. in terms of population, language, year limits, type of publication, study type).*	Protocol and supplementary file 3.
**5**	Data sources	Describe the information sources used (e.g. *electronic databases (MEDLINE, EMBASE, CINAHL, psycINFO, Econlit), grey literature databases (digital thesis, policy reports), relevant organisational websites, experts, information specialists, generic web searches (Google Scholar) hand searching, reference lists)* and when the searches conducted; provide the rationale for using the data sources.	Protocol and supplementary file 3.
**6**	Electronic Search strategy	Describe the literature search *(e.g. provide electronic search strategies with population terms, clinical or health topic terms, experiential or social phenomena related terms, filters for qualitative research, and search limits)*.	Protocol and supplementary file 3.
**7**	Study screening methods	Describe the process of study screening and sifting *(e.g. title, abstract and full text review,*	Protocol and page 5.

For peer review only - http://bmjopen.bmj.com/site/about/guidelines.xhtml

No	Item	Guide and description	Reported on page #
		number of independent reviewers who screened studies).	
**8**	Study characteristics	Present the characteristics of the included studies *(e.g. year of publication, country, population, number of participants, data collection, methodology, analysis, research questions).*	Page 7, table 1 and supplementary file4.
**9**	Study selection results	Identify the number of studies screened and provide reasons for study exclusion *(e,g, for comprehensive searching, provide numbers of studies screened and reasons for exclusion indicated in a figure/flowchart; for iterative searching describe reasons for study exclusion and inclusion based on modifications t the research question and/or contribution to theory development).*	Figure 1.
**10**	Rationale for appraisal	Describe the rationale and approach used to appraise the included studies or selected findings *(e.g. assessment of conduct (validity and robustness), assessment of reporting (transparency), assessment of content and utility of the findings).*	Protocol, page 5 and supplementary file 4.
**11**	Appraisal items	State the tools, frameworks and criteria used to appraise the studies or selected findings *(e.g. Existing tools: CASP, QARI, COREQ, Mays and Pope*[25]*; reviewer developed tools; describe the domains assessed: research team, study design, data analysis and interpretations, reporting).*	Protocol, pages 5-6 and supplementary file 4.
**12**	Appraisal process	Indicate whether the appraisal was conducted independently by more than one reviewer and if consensus was required.	Protocol, pages 5-6, supplementary file 4.
**13**	Appraisal results	Present results of the quality assessment and indicate which articles, if any, were weighted/excluded based on the assessment and give the rationale.	Page 11, supplementary file 4.

For peer review only - http://bmjopen.bmj.com/site/about/guidelines.xhtml

No	Item	Guide and description	Reported on page #
**14**	Data extraction	Indicate which sections of the primary studies were analysed and how were the data extracted from the primary studies? *(e.g. all text under the headings "results /conclusions" were extracted electronically and entered into a computer software).*	Protocol and page 5.
**15**	Software	State the computer software used, if any.	Protocol and page 5.
**16**	Number of reviewers	Identify who was involved in coding and analysis.	Page 5 and supplementary file 5.
**17**	Coding	Describe the process for coding of data *(e.g. line by line coding to search for concepts).*	Page 5 and supplementary file 5.
**18**	Study comparison	Describe how were comparisons made within and across studies *(e.g. subsequent studies were coded into pre-existing concepts, and new concepts were created when deemed necessary).*	Supplementary file 5.
**19**	Derivation of themes	Explain whether the process of deriving the themes or constructs was inductive or deductive.	Supplementary file 5.
**20**	Quotations	Provide quotations from the primary studies to illustrate themes/constructs, and identify whether the quotations were participant quotations of the author's interpretation.	Table 2 and pages 11-15
**21**	Synthesis output	Present rich, compelling and useful results that go beyond a summary of the primary studies (e.g. *new interpretation, models of evidence, conceptual models, analytical framework, development of a new theory or construct).*	Pages 20-25.

3

For peer review only - http://bmjopen.bmj.com/site/about/guidelines.xhtml

Supplementary File 3- Full details of search strategy and selection criteria

Full details are available in the published protocol[51] but are detailed briefly below.

Selection criteria

Participants

Individuals with a clinical or genetic diagnosis of heterozygous familial hypercholesterolaemia (FH). No restrictions were placed on age or history or cardiovascular disease (CVD). Individuals with homozygous FH were not included.

Phenomena of interest

The experiences and beliefs of individuals with FH, and their family members, regarding their condition, its long-term health consequences and treatment.

Types of studies

Only papers reporting primary qualitative data were included. Questionnaire studies were not included. Papers reporting both quantitative and qualitative data were included if the qualitative data could be independently extracted. Multiple papers reporting findings from the same sample of participants were included if they reported unique data.

Intervention/exposure

Treatment was defined as any behavioural action undertaken by an individual in an effort to manage their FH diagnosis.

Setting

No restrictions were placed on the country in which study was conduction, nor the location at which data was collected from individuals.

Search Strategy

MEDLINE, Embase, PsycINFO (via OVID), Cochrane library and CINAHL databases were searched from inception to 05/09/2018. We used a validated qualitative search filter[121] and population specific search terms. The search strategy that was used in MEDLINE is displayed in Appendix 1. The OpenGrey database and specialist websites (HEART UK, British Heart Foundation, The FH Foundation and The Simon Broome Register) were also searched up until 05/09/2018. The reference lists of the 50 papers taken to the full text screening stage were also hand searched. When only an abstract was available, the lead author was conducted in attempt to retrieve the full text. When contacting the lead authors of the included papers as part of the quality appraisal stage, enquiries were also made about any unpublished work.

For peer review only - http://bmjopen.bmj.com/site/about/guidelines.xhtml

Appendix 1: Search Strategy used in MEDLINE 05/09/2018

1. (familial adj1 hypercholesterolemia).ti,ab, kf.

2. (familial adj1 hypercholesterolaemia).ti,ab, kf.

3. (inherit* adj1 high adj1 cholesterol).ti,ab, kf.

4. *Hypercholesterolemia/ge [Genetics]

5. 1 or 2 or 3 or 4

6. interview*.ti,ab.

7. exp. Interviews/

8. experience*.tw.

9. qualitative.ti,ab.

10. 6 or 7 or 8 or 9

11. 5 and 10

DRAFT

For peer review only

For peer review only - http://bmjopen.bmj.com/site/about/guidelines.xhtml

Supplementary File 4- Quality appraisal methodology and results

Methodology

The CASP tool, endorsed by the Cochrane Collaboration[(53),] asks 10 questions relating to the rigour of the methodology used, quality of reporting and relevance of findings. To ensure comprehensive evaluation of methodological quality, these questions were answered with further consideration of 12 criteria produced by an expert panel.[122] As the purpose of the quality appraisal was to determine the methodological strengths and limitations of studies included in the synthesis, the lead authors of each paper were contacted to obtain further information in an attempt to overcome the recognised issued of poor reporting in qualitative research. Information from multiple papers involving the same sample was pooled when appropriate.[55, 57] Each author was given 1 month to respond. Two reviewers (AS, FK) independently appraised each study, assigning a rating of 0, 1 or 2 for each question which reflected the extent to which the obtained information from paper and author answered the criteria (0=not addressed, 1=partially addressed, 2=fully addressed). The reviewers then met to come to a consensus of individual and total scores, resolving differences through discussion. The reviewers then decided upon threshold for low, medium and high rated quality that they felt adequately captured the quality of the included papers.

Summary of results

Table 1 displays the CASP score breakdowns for each paper. Table 2 displays further details of the methodological limitations and transferability considerations of each included paper.

For peer review only - http://bmjopen.bmj.com/site/about/guidelines.xhtml

Table 1: CASP appraisal scores of included studies

Sample number	Reference	Was there a clear statement of research aims?	Is qualitative methodology appropriate?	Was the research design appropriate to address the aims of the research?	Was the recruitment strategy appropriate to the aims of the research?	Were the data collected in a way that addressed the research issue?	Has the relationship between researcher and participants been adequately considered?	Have ethical issues been taken into consideration?	Was the data analysis sufficiently rigorous?	Is there a clear statement of findings?	Is the research valuable?	Overall score (out of 20)	Did author provide further information?
**1**	(64)	2	2	2	2	2	0	1	0	1	1	**13**	**NO**
**2**	(65)	2	2	2	1	1	1	0	1	1	2	**13**	**NO**
**3**	(61)	2	2	2	2	2	2	2	2	2	2	**20**	**YES**
	(77)	2	2	2	2	2	2	2	2	1	2	**19**	**YES**
	(78)	2	2	2	1	2	2	2	2	2	1	**18**	**YES**
	(38)	2	2	2	2	2	2	2	2	2	2	**20**	**YES**
**4**	(66)	2	2	2	2	2	1	2	1	1	2	**17**	**YES**
	(68)	2	2	2	1	2	0	2	1	1	2	**15**	**YES**
	(67)	2	2	2	1	2	0	2	1	0	1	**13**	**YES**
**5**	(37)	2	2	2	2	2	1	2	1	2	2	**18**	**YES**
**6**	(69)	2	2	2	2	0	0	2	1	1	1	**13**	**NO**
**7**	(70)	2	2	2	2	2	1	2	2	2	2	**19**	**YES**
**8**	(71)	2	2	2	2	2	0	2	1	1	2	**16**	**YES**
**9**	(72)	2	2	2	2	2	1	2	2	1	1	**17**	**YES**
**10**	(73)	2	2	2	2	2	2	1	2	2	2	**19**	**NO**
	(39)	2	2	2	2	2	2	1	2	2	2	**19**	**NO**
**11**	(74)	2	2	2	2	2	1	2	2	2	2	**19**	**YES**
**12**	(75)	2	2	2	1	1	0	0	1	1	1	**11**	**NO**
**13**	(62)	2	2	2	2	2	2	2	2	2	2	**20**	**NO**
**14**	(63)	2	2	2	1	2	1	2	1	2	2	**18**	**YES**
	(40)	2	2	2	1	2	1	2	1	2	2	**17**	**YES**
	(79)	2	2	2	1	2	1	2	1	1	1	**16**	**YES**
	(80)	2	2	2	1	2	1	2	1	1	2	**16**	**YES**
**15**	(76)	2	2	2	2	1	0	0	1	1	1	**12**	**NO**

Scoring system: 0=No criteria fulfilled or can't tell; 1= some criteria fulfilled; 2= All criteria fulfilled. In reference to the criteria suggested for each question by CASP tool(53) and further criteria as described by Santiago-Delefosse et al.(122)

For peer review only - http://bmjopen.bmj.com/site/about/guidelines.xhtml

Table 2: Summary of methodological limitations and transferability considerations of the included papers

Sample number	Reference	CASP quality SCORE & rating	Methodological and reporting limitations	Transferability considerations of sample
**1**	(64)	13 Low	Lack of details provided about the rigour of the analysis process.<br>Authors self-selected the data from interviews to transcribe.<br>Data saturation not discussed.<br>Relationship between researcher and participants was not adequately considered.<br>Credibility of findings and the limitations of study design not addressed when reporting the findings.<br>No details of informed consent or if participants were told about data confidentiality or their right to withdraw.	No sampling strategy used but sample comprised of a good range of ages, genders, history of CVD events and age of diagnosis.<br>All recruited from one clinic.<br>All from Sweden.
**2**	(65)	13 Low	Ethical issues not addressed.<br>Group meetings may have resulted in lack of representative findings as certain individuals may have dominated the conversations or individuals may have felt unable to voice their own opinions.<br>Lack of disconfirming cases presented<br>No details of informed consent or if participants were told about data confidentiality or their right to withdraw.	All very motivated and engaged individuals to volunteer for this group.<br>Many receiving apheresis treatment.
**3**	(61)	20 high	Study limitations not addressed when reporting the findings.	All motivated to seek treatment as active attendees of lipid clinic.<br>Majority young (70% 10-39 years) and asymptomatic.<br>Large (40) sample size.<br>All from Norway.<br>All recruited from one lipid clinic.
	(77)	19 High	Lack of disconfirming cases presented and discussion against the findings.	
	(78)	18 High	Lack of disconfirming cases presented and discussion against the findings.	
	(38)	20 High	Study limitations not addressed when reporting the findings.	
**4**	(66)	17 Medium	Lack of details provided about the rigour of the analysis process<br>Relationship between researcher and participants was not adequately considered	All participants regularly attend lipid clinics and opted in for DNA testing.<br>Relatively well education (42% university education).<br>All participants from Scotland.<br>No sampling strategy used so likely not representative.<br>Half of patients from professional/skilled non-manual background.<br>Ethnicity not provided but authors state majority white British.<br>Recruited from two lipid clinics.
	(68)	15 Medium	Credibility of findings and the limitations of study design not addressed when reporting the findings<br>Relationship between researcher and participants was not adequately considered.	
	(67)	13 Low	Lack of details provided about the rigour of the analysis process.<br>Credibility of findings and the limitations of study design not addressed when reporting the findings.<br>Relationship between researcher and participants was not adequately considered.	
**5**	(37)	18 High	Analysis carried out by one individual only with no independent verification of themes conducted.<br>Relationship between researcher and participants was not adequately considered.	Sample not randomly selected.<br>Recruited from one clinic.<br>All live in metropolitan Perth, Australia.
**6**	(69)	13 Low	Lack of disconfirming cases presented and arguments against findings.<br>Data saturation not discussed.<br>Relationship between researcher and participants was not adequately considered.<br>Credibility of findings and the limitations of study design not addressed when reporting the findings.	Recruited across 11 lipid clinics.<br>All from the U.K.<br>All recently identified as being at risk of FH and during study received either clinical or DNA test results.<br>Sample included in analysis includes participates with DNA positive and Non-DNA positive diagnosis.<br>Majority (14/19) white British.
**7**	(70)	19 High	Relationship between researcher and participants was not adequately considered.	All Swedish.<br>All recruited from one lipid clinic.
**8**	(71)	16 Medium	Credibility of findings and the limitations of study design not addressed when reporting the findings.<br>Relationship between researcher and participants was not adequately considered.	All had consented to genetic testing.<br>All from Scotland.

For peer review only - http://bmjopen.bmj.com/site/about/guidelines.xhtml

			Analysis carried out by one individual.	All participants white, and majority highly educated.<br>Majority of participants asymptomatic.<br>Patients were self-selected from HCP who excluded participants if they felt they were too vulnerable, which included if had experienced a recent bereavement.<br>13 of parents had FH, 4 were spouses of those with FH
**9**	(72)	17 Medium	Lack of results to support conclusions drawn.<br>Credibility of findings and the limitations of study design not addressed when reporting the findings.	All asymptomatic.<br>Only 2 FH patients and 1 relative of FH patient.
**10**	(73)	19 High	No details of informed consent or if participants were told about data confidentiality or their right to withdraw.	Most participants had private medical insurance, were white and all actively engaged with the healthcare system.<br>All recruited from same healthcare system.<br>All patients from Massachusetts, U.S.A.
	(39)	19 High	No details of informed consent or if participants were told about data confidentiality or their right to withdraw.	
**11**	(74)	19 High	Relationship between researcher and participants was not adequately considered-three interviewers carried out the interviews and the potential bias this may incur was not addressed	All recruited from one health intuition.<br>All engaged with healthcare system and willing to talk about their condition.<br>11/16 were females.
**12**	(75)	11 Low	Lack of details provided about the study methodology or rigour of analysis process.<br>Data saturation not discussed.<br>Relationship between researcher and participants was not adequately considered.<br>Credibility of findings and the limitations of study design not addressed when reporting the findings.<br>Ethical issues not addressed.	Half participants were reaching treatment goals, half were not.<br>All recruited from one genetic centre.<br>All Danish.<br>Only 1 female in the group of patients reaching treatment targets
**13**	(62)	20 High	Coding and analysis of data was primarily independent, with the student's supervisors only overseeing it.	Sample comprised of non-attenders at clinic-not been seen for at least 2 years<br>Wide geographical spread, but all participants from Norway<br>Participants recruited from one clinic
**14**	(63)	18 High	Analysis by single researcher and potential bias not addressed<br>Data saturation not discussed.<br>Relationship between researcher and participants was not adequately considered.	Quota sampling used but all were white and majority (28/31) white British, 65% were ≥46 years old and 50% from professional occupations.<br>Participants recruited from one clinic.<br>Half self-reported experiencing some form of CHD.<br>All from North England, U.K.<br>All attended lipid clinic for at least 1 year, most for substantially longer.
	(40)	17 Medium	Analysis by single researcher and potential bias not addressed.<br>Data saturation not discussed.<br>Relationship between researcher and participants was not adequately considered.<br>Credibility of findings not addressed when reporting the findings.	
	(79)	16 Medium	Analysis by single researcher and potential bias not addressed.<br>Data saturation not discussed.<br>Relationship between researcher and participants was not adequately considered.<br>Credibility of findings not addressed when reporting the findings.	
	(80)	16 Medium	Analysis by single researcher and potential bias not addressed.<br>Data saturation not discussed.<br>Relationship between researcher and participants was not adequately considered.<br>Credibility of findings not addressed when reporting the findings.	
**15**	(76)	12 Low	Lack of details provided about the study methodology or rigour of analysis process.<br>Ethical issues not addressed.<br>Data saturation not discussed.<br>Credibility of findings or limitations of study methodology not addressed when reporting findings.<br>Relationship between researcher and participants was not adequately considered.	All motivated to participate in research as recruited from ongoing trial.<br>All lived in central London.<br>All clinical diagnosis, but 5 had DNA diagnosis confirmed and 2 had negative DNA test.

DRAFT
For peer review only

For peer review only - http://bmjopen.bmj.com/site/about/guidelines.xhtml

Supplementary File 5: Full details of thematic synthesis methodology

Stage 1: Line by line coding

In our protocol we originally planned to analyse the extracted data according to our review questions regarding factors influencing adherence to treatment. However, few studies directly addressed this question, therefore the authors put these review questions to one side for the data extraction process and revisited them for the coding stage.

Two reviewers (AS, FK) had previously read all papers independently for the critical appraisal stage. They were therefore familiar with the papers and had discussed them. At this stage they independently reread and coded, on paper, the extracted data from seven papers. The process involved line by line coding of the extracted data in which each line of text was assigned a free code according to its meaning and content. The codes were inductively created in response to the findings uncovered. The two reviewers then met to discuss and compare their findings before then deciding upon a preliminary coding frame which they then used when coding the extracted data from three further papers independently. In addition, new codes were created when necessary and the reviewers met again to discuss the findings, making revisions to the coding frame. One reviewer (FK) then independently coded the extracted data from each paper using Nvivo software. The coding frame was modified and added to throughout this process, with any changes made discussed with a second reviewer (AS). By this stage, no new codes were being identified, but some codes were consolidated into one code and others given more clarification about their meaning. A copy of the finalised coding frame is available to view (Appendix 1). A second reviewer (AS) performed secondary coding on 10% of the papers (three papers) before meeting with FK to compare findings and ensure consistency of interpretation.

This line by line coding facilitated the translation of concepts from one study to another- a key component of qualitative synthesis. Most sentences were categorised using more than one code as a result of having content which had more than one possible meaning e.g. ‘perceived risk’ and ‘relative risk’ or ‘perceived seriousness’ and ‘emotional impact’.

Stage 2: Development of descriptive themes

This stage involves the development of initial descriptive themes based upon the raw data that closely reflect the aggregative findings of the included studies.[54] The two reviewers who had carried out the coding (AS, FK) met with a third reviewer (EW) to discuss the findings of

For peer review only - http://bmjopen.bmj.com/site/about/guidelines.xhtml

the coding process. One reviewer (FK) produced summary reports of each of the 19 identified free codes which provided an overview of the findings across the papers including illustrative quotes and disconfirming cases. The summaries were descriptive in nature and avoided any interpretation. These summaries formed the basis of discussion between the three reviewers. At this second stage, the discussion was carried out in the context of the first research question- what are the experiences and beliefs of individuals' in relation to their condition, its associated morbidity and mortality risk and treatment?'. The discussion was exploratory in nature and no *priori* framework was imposed upon the findings at this stage. The aggregative findings of the studies, as consolidated in the code summaries, were deliberated, with examination of any similarities, differences and relationships between codes explored. From this discussion, 20 descriptive themes were identified. These descriptive themes were reflective of prevalent and persistent findings across the studies. Some of these themes were reflective of original codes used in the coding process, others were new themes created to capture more specific and detailed aspects of the original findings of coding process. For example, the findings captured using the code 'family influence' were further categorised into the descriptive themes 'parental influence upon treatment related behaviours' and 'FH and its treatment becomes normalised within families'.

One reviewer (FK) then produced a draft summary of these descriptive themes which was reviewed and discussed with AS and EW before a final version was agreed upon.

Stage 3: Development of analytical themes

The generated descriptive themes captured and aggregated the beliefs and experiences of individuals with FH in relation to their condition and its treatment. The third stage of thematic synthesis aims to go beyond the primary content of the original papers to generate additional concepts or understandings.[54] This is considered an essential component of any qualitative synthesis methodological approach.[55,56] In this review, this meant using the descriptive themes to answer our research questions regarding how these beliefs and experiences may influence an individuals' adherence to treatment and to identify any enablers and/or barriers to this.

This was achieved by first examining each descriptive theme individually in the context of treatment adherence through consideration of the relationship between the content captured in each descriptive theme and patients ability and/or inclination to adhere to treatment. Secondly, any relationships between the descriptive themes were explored to identify

For peer review only - http://bmjopen.bmj.com/site/about/guidelines.xhtml

common factors. Each reviewer (FK, AS, EW) carried this out independently before meeting as a group to discuss further. From these discussions, over-arching analytical themes were identified. These analytical themes were then deliberated in the context of identifying enablers and barriers to treatment adherence which could be used to inform clinical practice, policy development and research intervention design. The reviewers met on three occasions to discuss their findings collaboratively. It was an iterative process in which the analytical themes were modified until the reviewers felt they adequately explained all the initial descriptive themes and identified enablers and barriers to treatment.

For example, 3 of descriptive themes related to the involvement of other family members in an individuals' experiences of having FH and its treatment (FH and its treatment become normalised within families, parental influence upon treatment related behaviours and desire to protect children). From these descriptive themes, the reviewers identified the importance of the behaviours and beliefs of other family members upon an individuals ability and receptivity to adhering to treatment. This finding was captured in the analytical theme entitled 'family influence'. From this analytical theme, the reviewers identified two enablers to treatment adherence. These enablers were the delivery of care and treatment advice through family-based clinics and the commencement of treatment from a young age.

For peer review only - http://bmjopen.bmj.com/site/about/guidelines.xhtml

95 Appendix 1: Finalised coding frame

Code	Brief Description
Understanding/biological knowledge of FH	Account/description of what FH is, their understanding of its aetiology, its genetic transmission, its effect upon their body, any symptoms and any associated short and long term health implications
Perceived risk	The perceived risk of FH as a condition. Their thoughts/beliefs of short and long term health consequences of FH. Both genetic and behavioural associated risk.
Perceived seriousness	How serious/important FH and/or it's associated health consequences are believed to be
Family history	Account/description of family history of FH diagnosis, treatment and/or adverse outcomes such as death/serious illness
Life events	Significant milestones/occasions in life i.e. becoming parent, leaving school, getting married, ageing
Co-morbidities	Other illnesses/conditions that are not FH
Relative risk	Participant compares own risk to that of another person (family member, peer, abstract person) or to risk associated with another condition/illness
Management of condition	Account/description of the use/role of medicine or lifestyle in the treatment of FH.
Perceived efficacy of treatment	Perceptions/beliefs of the effectiveness of treating FH (medication, lifestyle and other)
Self-efficacy	The perception of an participant upon their own ability to follow treatment recommendations
Enablers and barriers for treatment	Any factors that help, enable, motivate OR Any factors that demotivate, stop or hinder a participant to seek and/or follow treatment advice
Ownership/personal responsibility	How a participant reflects/describes their perceived ownership of their condition and it's treatment. How much they perceive the condition to be their responsibility to manage/treat.
Emotional impact	Any emotion that FH diagnosis, management and/or associated health outcomes evokes in participants. Includes perceived stigma.
Impact on life	Any change participant has made to their life (everyday or longer term) as a result of their diagnosis of FH or its treatment
Professional support	Account/description of any involvement of healthcare professionals and/or medical procedures
Social support	Account/description of the role of family and/or friends in a patients' experience of their condition and it's management. practical or emotional support that individuals receive with regard to managing FH - i.e, treatment adherence.
Family influence	The influence of participants family upon their decision and ability to seek/adhere to treatment. Individuals' awareness of how others in their family network have dealt with screening and treatment and making decisions based on what other family members have done.
Information/help seeking	Accounts/descriptions of information or resources that participants would find useful
Parental views	Accounts/descriptions/thoughts/beliefs of parents in relation to their children.

96

For peer review only - http://bmjopen.bmj.com/site/about/guidelines.xhtml

BMJ Open

Enablers and barriers to treatment adherence in heterozygous familial hypercholesterolaemia: A qualitative evidence synthesis

Journal:	*BMJ Open*
Manuscript ID	bmjopen-2019-030290.R1
Article Type:	Research
Date Submitted by the Author:	01-May-2019
Complete List of Authors:	Kinnear, Fiona; University of Bristol, NIHR Bristol Biomedical Research Centre (Nutrition theme)<br>Wainwright, Elaine; Bath Spa University, Psychology department<br>Perry, Rachel; University of Bristol, NIHR Bristol Biomedical Research Centre (Nutrition Theme)<br>Lithander, Fiona; University of Bristol, NIHR Bristol Biomedical Research Centre (Nutrition Theme)<br>Bayly, Graham; University Hospitals Bristol NHS Foundation Trust, Department of Clinical Biochemistry<br>Huntley, Alyson; University of Bristol, Bristol Medical School<br>Cox, Jennifer; University of Bristol, NIHR Bristol Biomedical Research Centre (Nutrition Theme)<br>Shield, Julian; University of Bristol, NIHR Bristol Biomedical Research Centre (Nutrition Theme)<br>Searle, Aidan; University of Bristol, NIHR Bristol Biomedical Research Centre (Nutrition Theme)
**Primary Subject Heading**:	Qualitative research
Secondary Subject Heading:	Evidence based practice, Cardiovascular medicine, Health services research, Nutrition and metabolism, Patient-centred medicine
Keywords:	NUTRITION & DIETETICS, PREVENTIVE MEDICINE, QUALITATIVE RESEARCH, Coronary heart disease < CARDIOLOGY, Paediatric cardiology < CARDIOLOGY, Lipid disorders < DIABETES & ENDOCRINOLOGY

SCHOLARONE™
Manuscripts

Enablers and barriers to treatment adherence in heterozygous familial hypercholesterolaemia: A qualitative evidence synthesis

Fiona J Kinnear[1,2] Corresponding author, Elaine Wainwright[3], Rachel Perry[2], Fiona E Lithander[2], Graham Bayly[4], Alyson Huntley[5], Jennifer Cox[2], Julian P Hamilton-Shield[2], Aidan Searle[2].

1. Email Address: Fiona.kinnear@bristol.ac.uk; Telephone number: 0117 3421754
2. NIHR Bristol Biomedical Research Centre Nutrition Theme, University Hospitals Bristol NHS Foundation Trust and the University of Bristol, Bristol, UK
3. Psychology Department, Bath Spa University and Honorary Research Fellow, Department for Health, University of Bath, Bath, UK
4. Department of Clinical Biochemistry, University Hospitals Bristol NHS Foundation Trust, Bristol, UK

5. Population Health Science, Bristol Medical School, The University of Bristol, Bristol, UK

DRAFT For peer review only

Word count excluding title page, abstract, references, figures and tables: 5700

For peer review only - http://bmjopen.bmj.com/site/about/guidelines.xhtml

ABSTRACT

Objectives Individuals with heterozygous familial hypercholesterolemia (FH) are at high risk of developing cardiovascular disease (CVD). This risk can be substantially reduced with lifelong pharmacological and lifestyle treatment however research suggests adherence is poor. We synthesised the qualitative research to identify enablers and barriers to treatment adherence

Design Thematic synthesis of qualitative studies

Data sources MEDLINE, Embase, PsycINFO via OVID, Cochrane library and CINAHL databases and grey literature sources were searched through September 2018

Eligibility criteria We included studies conducted in individuals with FH, and their family members, which reported primary qualitative data regarding their experiences of and beliefs about their condition and its treatment.

Data extraction and synthesis Quality assessment was undertaken using the Critical Appraisal Skills Programme for qualitative studies. A thematic synthesis was conducted to uncover descriptive and generate analytical themes. These findings were then used to identify enablers and barriers to treatment adherence for application in clinical practice.

Results 24 papers reporting the findings of 15 population samples (246 individuals with FH and 13 of their family members) across eight countries were included. Data captured within 20 descriptive themes were considered in relation to treatment adherence and six analytical themes were generated: risk assessment; perceived personal control of health; disease identity; family influence; informed decision making; and incorporating treatment into daily life. These findings were used to identify seven enablers (e.g. 'commencement of treatment from a young age') and six barriers (e.g. 'incorrect and/or inadequate knowledge of treatment advice') to treatment adherence. There was insufficient data to explore if the findings differed between adults and children.

Conclusions The findings reveal several enablers and barriers to treatment adherence in individuals with FH. These could be utilised in clinical practice to facilitate optimal adherence to lifelong treatment thereby minimising the risk of CVD in this vulnerable population.

PROSPERO registration number CRD42018085946

For peer review only - http://bmjopen.bmj.com/site/about/guidelines.xhtml

Strengths and limitations of this study

- This is the first thematic synthesis of the qualitative literature exploring the beliefs and experiences of individuals with familial hypercholesterolaemia to identify enablers and barriers to treatment adherence that can be targeted in clinical practice
- Robust procedures for conducting a thematic synthesis were adopted, informed by the Cochrane Qualitative Research Methods Group guidelines and they were reported in line with the Enhancing Transparency in Reporting the Synthesis of Qualitative Research statement
- The barriers and enablers were identified from themes which were representative of all the included studies, increasing their validity
- While included studies were conducted across eight countries, all were within the developed world which could limit the generalisability of the findings

For peer review only - http://bmjopen.bmj.com/site/about/guidelines.xhtml

INTRODUCTION

Heterozygous familial hypercholesterolaemia (FH) is one of the most common inherited genetic disorders, estimated to affect as many as 1 in 250 individuals worldwide.[1 2] Left untreated the exposure to chronically elevated levels of low density lipoprotein cholesterol (LDL-C) from birth confers an increased risk of cardiovascular disease (CVD),[2 3] with approximately 50% and 85% of affected women and men respectively experiencing a coronary event before the age of 65.[4] While this risk can be significantly reduced with early detection and treatment, many affected individuals remain at higher risk of premature CVD morbidity and mortality.[5-9] The most beneficial effects of treatment are evident in primary prevention before the onset of CVD.[5 10] With diagnostic rates are as low as 1% in some countries,[11] current efforts are focussed on identifying individuals with FH via screening and genetic testing programs.[12 13] Treated as outpatients and asked to follow lifelong treatment, it is critical to ensure that this increasing patient group are able to self-manage their disease. With many patients not reaching treatment targets[14-16] it is an area that warrants further investigation.

To improve adherence to treatment recommendations, an understanding of the factors affecting adherence is required. The American Heart Association (AHA) has recognised the need to gain a deeper understanding of the experiences of individuals with FH before addressing the further identified research gaps.[17] Preliminary research has found the beliefs and attitudes of FH patients towards the recommended treatment exert a significant effect upon their intention to engage in these behaviours.[18 19] Qualitative research can provide further insight to how these beliefs and attitudes are developed and the nature by which they may influence subsequent behaviours.[20] Its exploratory nature also allows for the identification of other factors influencing an individuals ability and motivation to comply with treatment.[21 22]

Qualitative research conducted in FH patients has found illness knowledge[23], risk perception[24], a lack of symptoms[25] and family history of disease[26] to influence treatment adherence. However, the transferability of these findings beyond the sample they are conducted in is limited.[27] Qualitative syntheses, which bring together the findings from individual qualitative studies, can be used to gain a more in depth understanding of the issue and identify common themes which are applicable to a wider range of contexts.[28 29] It is recognised as an important source of evidence to inform healthcare interventions and policy development[30-32] including those targeting treatment adherence[33-35] and is advocated by the World Health Organisation and the Cochrane Collaboration Group.[28 36]

Objectives

1. Identify how the experiences and beliefs of individuals with FH influence their adherence to pharmacological and lifestyle treatment recommendations

2. Explore if these findings differ between children and adults

For peer review only - http://bmjopen.bmj.com/site/about/guidelines.xhtml

3. Use the findings to generate new understanding of the enablers and barriers to treatment adherence to inform clinical practice

For peer review only - http://bmjopen.bmj.com/site/about/guidelines.xhtml

MATERIALS AND METHODS

The methods used for this qualitative synthesis are briefly described below with full details available in the published protocol[37] and on the PROSPERO database (registration number CRD42018085946). Minor deviations to the protocol were made, outlined in supplementary file 1. The Enhancing Transparency of Reporting the synthesis of Qualitative research (ENTREQ) statement[38] has been followed and a checklist is available in supplementary file 2.

Search strategy

A comprehensive, systematic and pre-planned search was conducted to find all available qualitative evidence-full details are available in supplementary file 3.

Selection Criteria

Participants: Individuals with a clinical or genetic diagnosis of heterozygous familial hypercholesterolaemia (FH). No restrictions were placed on age or history or cardiovascular disease (CVD). Individuals with homozygous FH were not included.

Phenomena of interest: The experiences and beliefs of individuals with FH, and their family members, regarding their condition, its long-term health consequences and recommended pharmacological and lifestyle change treatment.

Types of studies: Only papers reporting primary qualitative data were included. Questionnaire studies were not included. Papers reporting both quantitative and qualitative data were included if the qualitative data could be independently extracted. Multiple papers reporting findings from the same sample of participants were included if they reported unique data.

Intervention/exposure: Treatment was defined as any behavioural action undertaken by an individual in an effort to manage their FH diagnosis.

Setting: No restrictions were placed on the country in which study was conduction, nor the location at which data were collected from individuals.

Quality appraisal

The methodological quality of the studies was assessed using the Critical Appraisal Skills Program (CASP) tool for reviewing qualitative research.[39] As the purpose of the quality appraisal was to determine the methodological strengths and limitations of studies included in the synthesis, the lead authors of each paper were contacted to obtain further information in an attempt to overcome the recognised issued of poor reporting in qualitative research. Full details of how this tool was used are available in supplementary file 4.

Data extraction

For peer review only - http://bmjopen.bmj.com/site/about/guidelines.xhtml

Methodological and contextual information from each paper were extracted into a table designed for this review by two reviewers independently (FJK, JC) after piloting in five papers. Two reviewers (FJK, AS) independently reviewed all text under the results, conclusions and discussion headings of all papers, as well as any supplementary files. Any data identified to be relevant to the research questions were extracted electronically using a tool designed for this review. In instances in which multiple papers reported the findings from a single study, data from the primary paper PhD theses were extracted first, before supplementary publications were reviewed for any additional, unique data. Results were compared and discussed until agreement was reached.

Data analysis

Thematic synthesis[40], a widely accepted and commonly used approach in qualitative syntheses, was used.[41 42] It involved three stages: line by line coding of the extracted data, generation of descriptive themes and development of analytical themes. Using NVivo software, two reviewers (FJK, AS) carried out the coding independently. The subsequent stages were carried out collaboratively between three reviewers (FJK, AS, EW). To enhance transparency, full details are available in supplementary file 5. The findings were discussed with three clinicians (JPHS, GB, PD) currently providing care to individuals with FH to help develop feasible and relevant recommendations for clinical practice.

Sensitivity analysis

To ensure the quality appraisal results were used in a meaningful way,[41 43] post-hoc sensitivity analysis was carried out by three reviewers (FJK,AS, EW) to examine the extent to which the synthesis results were affected by exclusion of poor quality papers, described in full elsewhere.[44] It involved examining if any themes were lost when each paper was removed from synthesis and evaluate if there was a significant impact upon the 'thickness' of findings reported within each theme. 'Thickness' refers to the depth, scope and context of findings which could influence the transferability and credibility of the results to the wider FH patient population.[45] This was carried out through discussion between three reviewers (FJK, AS, EW).

Patient and public involvement

Patients or members of the public were not involved in this study.

RESULTS

For peer review only - http://bmjopen.bmj.com/site/about/guidelines.xhtml

The titles and abstracts of 990 unique citations identified by the searches were screened, with 50 progressing to screening at the full-text level. Twenty-six papers were excluded at this stage due to: the full text not being available (n=1), no primary qualitative data being presented in the findings (n=6), the study population not having a clinical diagnosis of FH or inability to selectively extract data from those with a diagnosis in a mixed population (n=16) and data not being relevant to the aims of this review (n=3). Multiple papers reporting findings from the same sample of individuals and three PhD papers[46-48], two of which had supplementary papers published in addition to the originally reported theses, were included. Each paper was considered to be a separate primary paper and referenced separately. In total, 24 papers were included in the synthesis, comprising of 18 original[23 25 46-61] and six supplementary papers[24 26 62-65] reporting the findings of 15 population samples (Figure 1).

Characteristics of studies and participants

In total, 264 individuals with FH and 13 family members were involved, aged 8-69 years. Seven papers[24 25 46 58 59 62 63] reported findings from three samples which included individuals under 18 years. Four papers reported parental views of having children with FH.[25 56 58 59] Full characteristics of the included papers and samples are presented in Table 1.

For peer review only - http://bmjopen.bmj.com/site/about/guidelines.xhtml

Table 1: Characteristics of included studies

Sample number	Author & date of paper	CASP quality rating a, b	Research aim	Country	Recruitment setting	Sample Size c	Sample characteristics	Data collection methods
1	Agard et al, 2005[49]	Low	To explore the extent to which FH influences the life of the patients affected	Sweden	Outpatients treated at lipid clinic	23	10 M & 13 F; Mean age 48yrs (range: 31-67yrs); 4 with or had Hx of CVD	Face to face SSI
2	DeAngelis et al, 2017[50]	Low	To determine individual and group patient ideas and priorities regarding ways to enhance their own health	U.S.A.	Patients & family from patient centred outcomes research institute and outpatient clinic	7	6 FH patients, 1 family member	15 group meetings
3	Frich, 2007[46]	High*	To explore how individuals with FH perceive and manage their condition	Norway	Specialist clinic for metabolic lipid disorders	40	20 M & 20 F; Mean age 31yrs (range 14-57yrs); 7 had CVD symptoms; 19 had children	Face to face SSI
	Frich et al, 2006[62]	High*	To explore how patients with diagnosis of FH understand and perceive their vulnerability to CHD					
	Frich et al, 2007[63]	High*	To explore how patients at risk of CHD portray candidates for CHD					
	Frich et al, 2007[24]	High*	To explore patients' experiences of guilt and shame with regard to how they manage FH					
4	Hallowell et al, 2017[51]	High*	To investigate index patients' experiences of undergoing DNA testing as part of screening programme	Scotland	Two lipid clinics	38	17 M & 21 F; Mean age 52.6yrs (range 18-67yrs); 31 had children; 16 educated to university level	Face to face in depth interviews, (1 online)
	Jenkins et al, 2013[53]	Medium*	To explore patient's interpretations of their DNA results for FH					
		Low *	To explore the concept of inter-embodiment and its potential for					

	Jenkins et al, 2013[52]		advancing sociological research into illness biography and genetic identity					
5	Hardcastle et al, 2015[23]	High*	To investigate the perceptions and experiences of patients with a genetic diagnosis of FH involved in a cascade screening programme. To explore how these patients conceptualise FH and how such beliefs affect treatment compliance and lifestyle changes	Australia	Lipid disorders clinic	18	10 M & 8 F; Mean age 50.2 yrs (range 25-74 yrs); 2 had CVD symptoms	Face to face SSI
6	Hollands et al, 2012[54]	Low	Examine the impact of disease risk assessments based on both genetic and non-genetic information, or solely non-genetic information	U.K.	Lipid clinics at 11 hospitals	20	12 M & 8 F; Mean age 30.9yrs for DNA diagnosed & 40.7yrs for non-DNA; 17 white, 1 white Asian, 2 black Caribbean	3 telephone interviews
7	Hollman et al, 2004[55]	High*	To describe the QOL and to understand the underlying meaning of the concept of QOL in patients with FH	Sweden	Outpatient clinic	12	6 M & 6 F; 20-69yrs; 7 had children; 3 university level education; no Hx of CHV	Face to face SSI
8	Keenan et al, 2018[56]	Medium*	To explore parent's views and experiences of genetic testing and early treatment of children with FH in Scotland, experiences of their children's care pathway and to identify any barriers or facilitators in testing and treatment uptake	Scotland	Clinical genetic services and lipid clinics from 3 sites	17	6 M & 11 F; 20-69yrs; all white; 12 had post-secondary qualifications; 3 symptoms or Hx of CVD	SSI (15 face to face, 2 over phone)
9	Kirkegaard et al, 2014[57]	Medium*	Explore how cholesterol reducing medication and risk of CVD are interpreted by asymptomatic patients with high cholesterol	Denmark	5 GP centres.	3	1 M & 2 F; 24-62yrs; no CVD symptoms	Face to face SSI
10	Mackie et al, 2015[58]	High	Explore how family medical history, family narratives of medical experiences and AYA medical experiences together function as 'experiential evidence' and influence screening and treatment decisions	U.S.A	Paediatric preventative cardiology practice	24	12 AYAs with FH and 12 parents of AYAs with FH (4 dyads)<br><br>AYAs:6 M &, 6 F; Mean age 18.4yrs; 9 white, 1 black and 1 Asian<br>Parents: 2 M & 10 F; Mean age	Face to face SSI with AYA and parent separately
	Sliwinski et al, 2017[25]	High	To examine challenges transitioning to adult care for young adults with FH, and their parents, in the context of 2 developmental tasks: transitioning from childhood to early adulthood and summing					

For peer review only - http://bmjopen.bmj.com/site/about/guidelines.xhtml

			responsibility for self-management of a chronic disease				49.3yrs; 1 Asian, 9 white	
11	Meulenkamp et al, 2008[59]	High*	To study the experiences of children identified by family screening who were found to be a mutation carrier for a genetic CVD	Netherlands	Paediatric lipid clinic	16 children from 10 families	5 M & 11 F; 8-17yrs Number & age of parents not given	Face to face SSI (children and parents separately)
12	Mortensen et al, 2008[60]	Low	Comparative study to examine the QOL impact of FH in patients who had and had not reached the target of treatment	Denmark	Centre of inherited CVD	10	6 M & 4 F; 20-72yrs; no CVD Hx	Focus groups
13	Urke, 2016[47]	High	Explore how young adults, who stopped attending lipid clinic for medical and nutritional consultations, managed challenges related to living with FH and to the lifelong treatment	Norway	Outpatient clinic	11	6 M & 5 F; Median age 29yrs (range 26-35 yrs); 8 educated to university levels	SSI (9 face to face 2 over phone)
14	Weiner, 2006[48]	High *	How much and in which way patients with FH and professionals involved with the condition construct FH and CHD as genetic conditions	England	Lipid clinic	31	17 M & 14 F; Mean age 52 yrs (range 24-69 yrs); 31 white; 15 with current CVD	Face to face SSI
	Weiner and Durrington, 2008[26]	Medium*	To explore patients' understanding and experiences of FH and the significance of the hereditary aspect of the condition					
	Weiner, 2009[64]	Medium*	Consider how people with FH construct FH, high cholesterol and CHD					
	Weiner, 2011[65]	Medium*	Explore the notion of genetic responsibility, focussing particularly on responsibilities to family and kin					
15	Senior et al, 2002[61]	Low	Investigate perceptions of having an inherited predisposition to heart disease in people diagnosed with, and receiving treatment for FH	England	2 lipid clinics	7	5 M & 2 F; 39-58 yrs	Face to face SSI

SSI= semi structured interview; M= male; F= female; CHD = coronary heart disease; CVD = cardiovascular disease; QOL = Quality of life; AYA= adolescent and young adult; Hx = history

[a] CASP score: high=18-20; medium=14-17; low quality=<14.

[b] Papers for which lead author provided requested further information are marked with *

[c] The sample size and characteristics describe only those in sample with clinically diagnosed heterozygous FH and their family members.

For peer review only - http://bmjopen.bmj.com/site/about/guidelines.xhtml

Quality Appraisal and Sensitivity Analysis

Appraisal scores of papers ranged from 11-20 out of 20, with eleven rated high, seven medium and six low. (Table 1) The most common methodological limitations uncovered were relating to ethical issues, researcher reflexivity and rigour of data analysis. Consideration of a researchers' potential influence and bias upon data collection and analysis was critically examined fully in seven papers,[24 25 46 47 58 62 63] partially in 10[23 26 48 50 51 55 57 59 64 65] and not addressed in seven.[49 52-54 56 60 61] Ethical approval was obtained, or reasons given for exemption, in all but two papers,[60 61] however participants were not provided adequate information about withdrawal and anonymisation of data processes in a further 4 papers.[25 49 50 58] The data analysis was carried out by one researcher only in seven papers[23 26 47 48 56 64 65] and it was unclear if more than one person was involved at each stage of analysis in four papers.[51 52 60 61]

Eight lead authors responded to our request for further information, providing information for 16 of the 24 papers. Five of the six papers rated as low-quality were papers for which the author did not respond. This reflects our belief that low ratings may be reflective of poor reporting rather than poor methodology, supporting to our decision not to exclude papers. The sensitivity analysis carried out found that the removal of the five poor quality papers had no significant effect upon the synthesis findings- in both the descriptive and analytical themes uncovered and the depth of the findings. More detailed information of methodological and transferability issues is available in supplementary file 4.

Data analysis

Six analytical themes were derived from the findings captured by 20 identified descriptive themes, as displayed in Table 2 alongside illustrative quotes. Table 3 shows the occurrence of the descriptive themes within the extracted data from the 24 papers. While each analytical theme has a direct influence upon treatment adherence, they are not exclusive in nature and inter-theme relationships are evident as displayed in the thematic schema in Figure 2. Additionally, some themes by their integrative nature, had a greater influence upon treatment adherence as indicated by the shaded boxes. There were insufficient data regarding children and young people to explore whether the findings differed from adults.

Seven enablers and six barriers to treatment adherence (Table 4) were uncovered during the analysis of these themes and are described alongside the analytical themes below. In this section 'treatment' refers to both lifestyle and medication behaviours, unless otherwise specified.

For peer review only - http://bmjopen.bmj.com/site/about/guidelines.xhtml

Analytical themes

Risk assessment

Individuals lived experience of their disease, coupled with their beliefs concerning its known risks, increased or decreased their sense of vulnerability to its long-term health consequences. Knowledge of how FH had affected family members was the most prevalent factor considered by individuals when assessing their risk. Individuals with lived experience of a family member being ill or dying prematurely due to FH, had a heightened sense of risk.[46 48 49 52 55 56 58-62] Individuals unaware of FH in their families or with family members living a life unaffected by its consequences, perceived themselves at lower risk:[46 52 56 58 61 62] 'My dad's now in his 70s…it's not something I feel particularly threatened about having.'[56]

As FH does not 'make you feel ill',[52] individuals found having FH 'easy to forget, and easy not to take seriously.'[47] This was salient amongst younger individuals without existing CVD symptoms[23 25 47 48 58 59 65] for whom '…cholesterol always comes last. It will never be a focus until something happens to me.'[47] Older individuals who had lived through, or were currently experiencing CVD, perceived themselves at higher risk.[23 56 61 62] Others framed their perception of risk in the context of the risk they believed other diseases presented, concluding that FH health consequences were not as serious:[23 47 48 51 53 54 61] 'I didn't think it was life threatening, like being told you've got cancer.'[23]

For the majority of individuals, their risk assessment led to a perception that FH did not present a great risk to their current or long-term health.[23 47-49 51 56 59-61] This mismatch between the perceived and actual risk has been identified as a barrier to treatment adherence.

Perceived personal control of health

Individuals acknowledged the threat that FH posed to their health, but there was a widely held belief that they had the ability to modify their own personal risk.[24 47 49 51 53-62] They recognised that this required active engagement with treatment[23-25 47 49-51 53-56 58 61 62] and held themselves accountable for managing their disease[23-25 47-51 53-58 60-62] experiencing a 'bad conscience'[49] and 'guilt'[63] when they did not meet the expectations they had set themselves. Treatment was perceived to be effective[24 47 49 51 53-62] with individuals viewing FH as 'treatable'[48] and 'controllable'.[23] In particular, medication was regarded by individuals to be a mandatory and effective component of treatment.[24 47 49 51 53-62] They believed FH could be 'solved'[59] with medication and lead to achievement of cholesterol levels 'like most people'.[23] While individuals spoke of their efforts to change their lifestyle behaviours,[24 25 47 49 51 53-62]however many believed their cholesterol levels would not be 'radically changed'[61] by doing so[47 48 58 60] as 'doesn't matter what I eat or how much exercise I'm still going to have high cholesterol without tablets'.[23]

For peer review only - http://bmjopen.bmj.com/site/about/guidelines.xhtml

This confidence in the ability to successfully self-manage their condition was identified as an enabler to treatment adherence. The perceived effectiveness of medication led to a devaluing of the importance of following lifestyle treatment[23 47 48 57 58 60] and this prioritisation of medication was identified as a barrier to adhering to lifestyle treatment.

Disease identity

Individuals placed great importance, especially in social situations, to emphasis that they were 'not to blame'[60] for their high cholesterol.[24 26 48 50 51 53 54 57 60 61 63] High cholesterol was associated with unhealthy lifestyles and individuals wished to distance themselves from this negative connotation.[24 48 54 57 60 61 63] A positive genetic test provided 'a definitive'[51], rather than a possible, explanation for their high cholesterol.[50 53 54] and positively influenced individuals perceptions and behaviours.[24 50 51 53 54]If individuals had been following treatment of their volition before the diagnosis, it helped 'reaffirm their commitment' 'reaffirm their commitment'[53] to treatment.[51 54]If they had been previously unaware of their condition it prompted them to seek treatment:[53 56] 'I know now and can take preventative measures'.[54] Therefore, receiving a formal diagnosis was identified as an enabler to treatment adherence as being given a medical explanation empowered individuals to take control of their condition through engaging with treatment.

Family influence

Parents expressed a high level of concern about the well-being of their affected children[25 48 50 51 53 56 58 59] and this parental responsibility to care for children was identified as another enabler of treatment adherence. They assumed responsibility to ensure their children adhered to medical and lifestyle treatment,[25 48 50 51 53 56 58 59] taking action to 'bring them up with healthy eating habits'[51] and 'make sure that they take their medication'.[48] This involvement was reflected in the finding of individuals attributing their current treatment knowledge and behaviours to their parents:[47-49]: 'everything I've learned from home'.[47] Parents also made treatment-related decisions on their behalf [25 48 50 53 58 59]until they were 'old enough to decide.'[56] As such, the early adulthood years presented a challenge for treatment adherence as the young adults transitioned from being under the care of their parents to assuming responsibility for their behaviours.[25 47]

Growing up surrounded by family members following treatment recommendations and establishing healthy behaviours from a young age was found to instil lifelong habits in individuals.[25 47 48 56 58 59] Those who had grown up from a young age alongside diagnosed family members spoke of their condition and its treatment as something that had become 'normalised'[47] as it was all they had ever known.[25 48 56 58 59] Those who had parents who had bad experiences of medication were apprehensive about taking tablets,[58] but for many it led to the view that taking medication was ordinary[56] and not a 'big deal'.[58]

Two enablers to treatment adherence were identified from these findings: commencement of treatment from a young age and having other family members following similar treatment regimes.

For peer review only - http://bmjopen.bmj.com/site/about/guidelines.xhtml

Informed decision making

Individuals lacked an in-depth understanding of their disease and its treatment,[23-25 47-51 56-59 61] with many having ‘unanswered questions’[49] and requesting more information.[25 49-51] Misconceptions and false information regarding the role of treatment for FH were prevalent:[24 25 47-49 51 56-59 61]: ‘you can actually eat a lot of fat and the medicine takes care of it.’[23] Individuals were worried about the longer-term impact of statin therapy on their, and their children’s, health[49 58] as ‘it is a recent drug, and you don’t know what the long term effect could be.’[56] Lived experience of side effects were reported by some individuals[49 58 60] and many more were fearful of developing them in the future[55 56 58] as ‘many others have severe side effects from what I’m taking’.[60] This incorrect and/or inadequate knowledge of treatment advice and concerns over the short- and long-term use of lipid lowering medication were identified as barriers to treatment adherence.

Individuals frequently mentioned their encounters with healthcare professionals HCPs,[23 24 46-48 50 52 53 56 57 59 60] viewing them as playing a ‘big role’[25] in their ‘team approach’[58] to the management of their FH. Regardless of whether individuals recalled these encounters in a positive[24 25 47 48 50 56 58]or negative[24 46 47 56 60] light, these interactions and relationships with HCPs influenced their understanding of FH and its treatment.

Integrating treatment into daily life

Individuals did not feel they had to make many changes to their everyday life as a result of their diagnosis.[23 47-49 51 54 61]Their disease did not them from ‘living the life they wanted’[47] or require consideration when making life decisions[23 47 49 54 61] such as having children.[48 51]However, when faced with other commitments, such as family and career obligations, individuals found it more difficult.[23 25 47 49 54 60 62] During these periods individuals tended to be less focused on managing their disease viewing it as something they could pick up again when they had more time and energy.[23 25 47 56 62]This prioritisation of other life events over the self-management of condition was identified as a barrier to treatment adherence.

The treatment recommendations were perceived to be simple to follow and to have little impact on their QOL.[23 47-49 51 53-56 61] However, this perception is in stark contrast to the actual lived experiences of following treatment- especially the lifestyle recommendations. Dietary advice was perceived to be restrictive and interpreted by individuals to mean they could not eat their favourite foods[24 25 47 48 57 59] or enjoy social occasions:[24 25 54 57 59 60] ‘I won’t bother eating food I don’t like, just to follow a certain diet’.[47] Additionally, individuals were concerned about the opinions of their peers in social situations in which they felt they had to make certain dietary choices.[25 47 48 59 60] These findings were prominent amongst younger individuals.[25 47 59]As a result, the dietary advice was the ‘most difficult aspect’[49] of treatment, with many reporting they struggled to follow them at all times.[23-25 47 48 57 59 60] This finding of dietary advice being perceived as difficult to follow was identified as a barrier to adherence.

For peer review only - http://bmjopen.bmj.com/site/about/guidelines.xhtml

Reflective of the difficulties faced when trying to follow treatment guidelines, individuals expressed a need for additional information [23 49 50 56] and 'guidelines in order to help you start that change'.[25] Some sought additional information from their HCPs[23 25 49 50 56], while others called for practical advice and educational resources[25 49 50 56], as 'everyone knows the theory, but putting it to practice is quite hard'.[23] From this, practical resources and support for following lifestyle treatment advice was identified as an enabler to treatment adherence.

DRAFT
For peer review only

Table 2: Analytical themes and their composite descriptive themes with illustrative quotes

Analytical theme	Descriptive themes	Illustrative quotes from participants (1st order)	Illustrative interpretations from authors (2nd order)
Risk assessment	FH is a silent disease	'not a condition that has any symptoms, that makes you feel ill or anything.'[52]	'The majority of interviewees did not look upon the condition as a disease…If they were not affected by a cardiac disease…they regarded themselves as healthy.'[49]
	Family history modifies perception of FH related threat to health	'I'm not going to get past sixty. Dad never got past sixty.'[53]	'To them, reaching the age of death of a parent with FH was anticipated with fear of having a heart attack themselves.'[60]
	FH is not as threatening to health as other conditions	'Its not that bad….Its not like having something like Huntington's or something like that.'[51]	They mentioned conditions with more drastic consequences such as allergies, epilepsy or diabetes.'[47]
Perceived personal control of health	FH is a manageable condition	'well it's treatable isn't it by diet and drugs. It's not something that's incurable.'[48]	'FH carrier children demonstrated high feelings of control over their condition.'[59]
	Individuals feel personally responsible for managing their FH	'it means you could be in danger of like what could possibly happen like in the future if you don't change anything.'[58]	'FH patients have a strong desire to empower themselves in order to improve their own health.'[50]
	FH medication is effective	'I believe that as I am taking the pills that my risk of heart attack is no greater than anyone else of my age or weight.'[61]	'Preventative medical treatment built confidence in the potential for living a long life.'[55]
	FH lifestyle treatment viewed as less important than medication	'I could never get that down no matter 'ow much dieting or exercise I do…so it can only be reduced through medication.'[48]	'Many tended to devalue the importance of lifestyle changes in controlling FH and place their hope in medication.'[23]
Disease identity	Importance of establishing that high cholesterol levels are not self-inflicted	'It enables me to emphasise that it is not my fault, that it's something inherited.'[62]	'they always described FH as a hereditary condition to underline that their cholesterol issues were not due to unhealthy lifestyle.'[60]
	Receiving genetic diagnosis provides certainty	'I guess it is a relief in a funny way because I had an answer to what was quite a surprising medical condition that I had…so at least I know now and can take preventative measures.'[54]	it provided an aetiological explanation and diagnostic label, confirmed current risk management practices…'[24]
The influence of family	Desire to protect children	'we want to help him…[so] we have decided to give him statins until he is 16…we've covered him until he's old enough to decide for himself.'[56]	'In fact, the main concern for the affected parents appeared to be the well-being of their children…'[49]
	Parental influence upon treatment related behaviours	'my parents, specifically my mom, were really integral in teaching us types of food to eat..'[25]	'AYAs expressed how their perceptions of their parents experience have influenced their perceptions of the respective treatment options.'[58]
	FH and its treatment become normalised within families	'Since I grew up with FH and had a relatively good diet and good habits and routines, it makes it easier.'[47]	'FH carrier children typically reported it had become habit to maintain a healthy, non-fat diet. Commonly the whole family, including the non-carriers, kept to the same diet restrictions.'[59]
Informed decision making	HCP interactions	'My daughter. I don't think she really understood what [high cholesterol] really meant until she came here and talked with doctor.'[58]	'The doctors presentation of FH, however, influenced all patients perceptions of the risk and severity of the diagnosis.'[60]
	Inadequate and/or incorrect knowledge about FH & treatment	'in the newspapers, the stories that you cut out butter, red meat, etc., and you'll be okay.'[61]	'Many informants still had unanswered questions or were felt to lack relevant knowledge.'[49]
	Concerns about side effects of FH medication	'would I be able to have children at all after taking all these medicines for years?'[49]	'Parents reported having strong concerns about statin treatment in children, not only because of their long-term safety but also potential side effects.'[56]
Inco rpor	FH and it's treatment does not have big impact upon life	'You don't have to plan your life around it. You don't have to wonder, can you have children or not.'[51]	'FH was not viewed as a significant burden, but more of a lifestyle adjustment, involving a healthy diet, exercise, and statin treatment from an early age.'[56]

For peer review only - http://bmjopen.bmj.com/site/about/guidelines.xhtml

ating treatmen t into daily life	Balancing FH treatment with other competing priorities	'Our two children, who were often ill….My husband…travelled all the time, so I almost had more than I could put up with at that moment.'[62]	'Young adults also articulated challenges maintaining diet and exercise regimes while adjusting to a new routine and environment at college or in workforce.'[25]
	Lifestyle advice treatment is restrictive and difficult to follow	'I've changed my diet as much as I can… don't want to bother too much and speculate, live an unworthy life and diet at the age of seventy. I'd rather be happy and die when I'm fifty.'[24]	'Making dietary changes had been the worst aspect of their condition, and this included people who already had CHD.'[62]
	Social implications of following FH treatment	'Some people comment on the things I eat. And then I'm like 'well actually I have to eat this because I've got FH and I have to watch my diet.'[54]	'10 young adults articulated how concern over peers' opinions or overt peer pressure-restricted social activities centered around eating.'[25]
	Desire for further support and guidance	I think having the resources [would make it easy to adhere to lifestyle treatment]…like seeing a nutritionist that can give you options….'[25]	'..expressed a desire to be able to access educational resources in one place and for a way to reach out to others who could provide solidarity, comfort and aid with management of FH.'[50]

AYA= adolescent and young adult; HCP = healthcare professional; CHD = cardiovascular heart disease

DRAFT
For peer review only

For peer review only - http://bmjopen.bmj.com/site/about/guidelines.xhtml

Table 3: Occurrence of descriptive themes across the included papers and samples [a]

Sample number	Paper	Descriptive themes																			
		FH is a silent disease	Family history modifies perception of FH related threat to health	FH is not as threatening to health as other conditions	FH is a manageable condition	Individuals feel personally responsible for managing their FH	FH medication is effective	FH lifestyle treatment viewed as less important that medication	Importance of establishing that high cholesterol levels are not self-inflicted	Receiving genetic diagnosis provides certainty	Desire to protect children	Parental influence upon treatment related behaviours	FH and its treatment become normalised within families	HCP relationships	Inadequate and/or incorrect knowledge about FH and its treatment	Concerns about side effects of FH medication	FH and its treatment does not have big impact upon life	Balancing FH treatment with other competing priorities	Lifestyle advice treatment is restrictive and difficult to follow	Social implications of following FH treatment	Desire for further support and guidance
1	Agard et al, 2005[49]	✓	✓		✓	✓	✓				✓	✓		✓	✓	✓	✓	✓	✓	✓	✓
2	DeAngelis et al, 2017[50]					✓				✓	✓			✓	✓						✓
3	Frich, 2007[46]		✓											✓	✓						
	Frich et al, 2006[62]		✓		✓	✓	✓											✓			
	Frich et al, 2007[63]								✓						✓						
	Frich et al, 2007[24]				✓	✓			✓	✓				✓	✓				✓	✓	
4	Hallowell et al, 2017[51]			✓	✓	✓	✓		✓	✓	✓	✓			✓		✓				✓
	Jenkins et al, 2013[53]			✓	✓	✓	✓		✓	✓	✓			✓			✓				
	Jenkins et al, 2013[52]	✓	✓											✓							
5	Hardcastle et al, 2015[23]	✓	✓	✓	✓	✓	✓	✓			✓			✓	✓		✓	✓	✓		✓
6	Hollands et al, 2012[54]					✓			✓	✓							✓			✓	
7	Hollman et al, 2004[55]		✓		✓	✓	✓									✓					
8	Keenan et al, 2018[56]		✓		✓	✓	✓				✓	✓		✓	✓	✓		✓	✓		✓
9	Kirkegaard et al, 2014[57]		✓			✓	✓	✓	✓					✓	✓				✓	✓	
10	Mackie et al, 2015[58]	✓	✓		✓	✓	✓	✓			✓	✓	✓	✓	✓	✓					
	Sliwinski et al, 2017[25]	✓				✓					✓	✓	✓	✓	✓			✓	✓	✓	✓
11	Meulenkamp et al, 2008[59]	✓	✓		✓	✓	✓				✓	✓	✓	✓	✓		✓		✓	✓	
12	Mortensen et al, 2008[60]				✓	✓	✓	✓	✓		✓			✓		✓		✓		✓	
13	Urke, 2016[47]	✓		✓	✓	✓	✓	✓				✓	✓	✓	✓		✓	✓	✓	✓	
14	Weiner, 2006[48]	✓	✓	✓	✓	✓	✓	✓	✓		✓	✓	✓	✓	✓		✓	✓	✓		
	Weiner and Durrington, 2008[26]								✓												
	Weiner, 2009[64]								✓												
	Weiner, 2011[65]	✓																			

For peer review only - http://bmjopen.bmj.com/site/about/guidelines.xhtml

15	Senior et al, 2002[61]		✓	✓	✓	✓	✓		✓					✓		✓				

HCP = healthcare professional

[a] Themes identified within supplementary papers were only documented if they were evident in extracted data not reported in the primary paper and vice versa.

DRAFT
For peer review only

For peer review only - http://bmjopen.bmj.com/site/about/guidelines.xhtml

Table 4: Identified enablers and barriers to treatment adherence

Enablers	Barriers
Other family members following treatment regime	Mismatch between perceived and actual risk
Commencement of treatment from a young age	Concerns over the use of lipid lowering medication
Parental responsibility to care for children	Prioritisation of medication over lifestyle treatment
Confidence in ability to successfully self-manage their condition	Lifestyle treatment is difficult to comply with
Receiving formal diagnosis of FH	Prioritisation of other life events
Practical resources & support for following lifestyle treatment	Inadequate and/or incorrect knowledge of treatment advice
A positive relationship with healthcare professional	

DRAFT
For peer review only

For peer review only - http://bmjopen.bmj.com/site/about/guidelines.xhtml

Discussion

This synthesis has produced new insights into the factors influencing treatment adherence in FH which have implications for clinical practice and future research.

We found that individuals did not perceive FH as a threat to their health except in those who had experienced symptoms of CVD or had a family history of FH related CVD, as previously reported by others.[66-69] This low perception of risk may be the result of the disease being relatively symptomless and the adverse consequences too far in the future to comprehend, This idea is reinforced by studies reporting heightened perceived risk amongst older individuals[70] and young adults perceiving their health to be average or above that of the general population.[16] The minimal threat to health may explain the findings that being diagnosed with FH does not increase psychosocial dysfunction in children[71 72], nor negatively impact upon self-reported quality of life (QOL) or rates of depression and anxiety in adults.[73-76] While these findings are positive, individuals who do not view their disease as a serious threat may be less motivated to adhere to treatment which may explain the findings of higher self-reported medication adherence in older individuals[77 78] and high non-adherence rates in individuals under 36 years.[79] These findings are concerning as individuals who do not adhere fully to treatment have been found to have higher levels of LDL-C.[77 79 80] Furthermore, while treatment has substantially reduced the risk of CVD individuals still remain at a higher risk than the general population.[9 81 82] This may be a consequence of LDL-C targets not being met by large numbers of treated adults[15 16 79 80 83] and children[84 85] and/or the presence of other risk factors independently associated with CVD.[86 87]

Our findings suggest this low risk perception may be mediated by beliefs that the risks are avoidable through effective treatment, in line with previous research.[16 66 72 88] These beliefs have been found to positively influence attitudes towards medication, increasing self-reported intentions to comply with medication [19] and rates of adherence.[89] However, individuals attitudes toward treatment behaviours may have a greater influence upon their intention to engage in treatment than their beliefs.[18] Our findings of negative attitudes toward certain aspects of treatment are therefore important to explore. We found individuals to perceive dietary recommendations as restrictive and impacting upon their QOL, as have others.[72 90] Some also believed they were unnecessary if taking medication, likely explaining low uptakes of lifestyle treatment compared to medication.[66 91] We also found negative attitudes towards medication due to side effects and anxieties about long-term safety, similar to others.[16 83 92] In contrast to these studies, we found anxiety about the development of side effects and complications of long-term use to be more prevalent than lived experience of side-effects. These negative attitudes are surprising as the dietary recommendations do not differ substantially from those for the general population and the safety and tolerability of statins have been demonstrated in adults[93] and children.[94-96]

For peer review only

DRAFT

Our finding of widespread inadequate knowledge of the treatment recommendations may explain the negative attitudes. It has been reported previously that awareness of the role of PA in treatment is low[97] and while individuals are mindful of the need for dietary treatment little is known about the depth of this knowledge.[72 90 97] This finding may be the result of the inconsistency in treatment advice provided with many not receiving the recommended lifestyle advice[91 98 99] or medication treatment[83 85 91 98 100 101] and for those that do, it is often not provided by HCPs with specialist FH knowledge.[91 99] As a result, we found many individuals are left wanting more information about treatment, in line with previous research,[91 97] This is concerning as many report using the internet to search for such information[91] which cannot be easily regulated and may be fuelling our further finding of a high prevalence of incorrect knowledge. Furthermore, individuals may be falsely interpreting negative media coverage of statin medication[102] to be relevant to their condition. This may be negatively influencing adherence to treatment as concerns about general medication overuse have been found to be heavily influential in shaping attitudes toward FH medication[19] Ensuring individuals have a comprehensive and factually correct understanding of the treatment recommendations is therefore essential to optimise adherence.

As this synthesis highlighted that parents take responsibility for their childs' treatment, it is important to ensure they are knowledgeable about the recommendations to help their children develop healthy habits from a young age. Previous research has found that children who follow dietary guidelines from a young age have more positive attitudes towards this aspect of treatment[71] and have improved dietary intakes in childhood[103-105] which are maintained into young adulthood.[106] Furthermore, forgetfulness is frequently reported as a reason for medication non-adherence[16 72 77 78 80 92] and starting treatment at a young age may help overcome this by instilling a routine, as found by others.[107] It is also important to ensure that when individuals reach an age where they become responsible for their own care, they themselves are equipped with the relevant knowledge to continue to make informed decisions. While there was insufficient data to draw conclusions about best practice for this age group, it appears that transitioning from living at home, adjusting to new routines and prioritising other things in life are common barriers to be targeted.[25 47]

Our findings also highlight the importance of receiving a genetic confirmation of FH. Receiving a medical diagnosis empowered individuals to take control of their condition, providing motivation to continue or commence medication and lifestyle treatments. The positive influence of diagnosis upon medication efficacy beliefs and adherence have been reported in previous research.[67 68 108 109] However, in contrast to our findings it has been reported that positive genetic results have either no effect[68] or weaken beliefs[108] regarding the efficacy of lifestyle treatment. However, in both cases the changes in beliefs did not have a negative impact upon their actual behaviours. Given our further finding that individuals find medical diagnosis useful in social situations, a common identified barrier to adhering to dietary recommendations, it

For peer review only - http://bmjopen.bmj.com/site/about/guidelines.xhtml

may be that genetic diagnosis exerts positive effect upon adherence beyond its influence of illness and treatment beliefs.

Strengths and limitations

Our thematic synthesis adhered to ENTREQ guidelines and used transparent and robust methodology. The comprehensive search strategy, involvement of more than one researcher at each stage of analysis, input from clinicians to corroborate the interpretation of the results and detailed appraisal of the included studies strengthen our findings. The analytical themes generated were produced from descriptive themes that were each evident across a large number of the included papers. The synthesis included data from 264 individuals with FH and 13 family members across eight countries, encompassing a wide range of ages, duration of diagnoses, primary and secondary CVD prevention and regional differences in healthcare provision. However, all individuals were from developed countries, the majority had high education levels and there were few from ethnic minority groups. This may limit the generalisability of the findings to all individuals with FH. Furthermore as the majority were recruited from lipid clinics and their beliefs may not reflect those opting out of treatment for their condition. Lastly, there were insufficient papers to explore if the factors influencing treatment adherence differ between adults and children with FH and care should be taken when extrapolating results to younger individuals.

Implications for clinical practice

We have identified seven enablers and six barriers to treatment adherence (Table 4) to be considered by any HCP delivering advice to individuals with FH and have produced the following 12 suggestions for clinical practice:

1. Ensure individuals are aware of the risk to their health, without instilling fear through emphasising the effectiveness of medical and lifestyle treatment

2. Where possible, ensure all individuals receive genetic confirmation of their condition

3. Communicate that despite the asymptomatic nature of the condition, adhering to treatment from a young age will deliver the greatest benefits to health

4. Discuss medication within an FH context, emphasising its necessity and distinguishing it from the use of medication in treatment of other causes of high cholesterol

5. Provide reassurance that medication is safe and side effects uncommon, with reference to relevant clinical guidelines indicating their safety for use by children highlighted to parents

6. Inform patients that side effects are specific to each type of medication and encourage discussion of any problems so alterative medications can be offered

For peer review only - http://bmjopen.bmj.com/site/about/guidelines.xhtml

7. Communicate dietary advice as being a lifestyle change rather than a restrictive diet with advice tailored to the individual needs and preferences of each individual

8. Ensure individuals have a factually correct understanding of the dietary recommendations and provide credible resources individuals can access if they require further support or guidance

9. The benefits of adhering to lifestyle treatment for management of their disease and their overall well-being, should be revisited at each clinic appointment

10. Treatment should begin early, with parents advised that prior to medication, dietary recommendations can be followed from the age of five. Non-affected family members can also be encouraged to follow guidelines, facilitating a family-based approach to aid adherence.

11. Treatment advice to be provided in family-based clinics if possible, or ensure adult and paediatric services are closely linked

12. Adolescent patients to be offered opportunity to transition to an adult clinic between the ages of 16-18 to take responsibility for their own treatment before they leave home

Some of our findings and clinical implications may be relevant to other chronic diseases which are asymptomatic in the early stages such as hypertension and Type II diabetes, for which treatment adherence rates are also low.[110 111]

Future research

With treatment most effective when started at a young age,[6 10 85] and our findings of a positive effect upon later life adherence, further qualitative research exploring the perspectives of children is required to allow HCPs to tailor advice to support maximal adherence during this crucial period. The findings of widespread inadequate and/or incorrect knowledge of the treatment recommendations warrants investigation into what advice is being given, and by whom. As individuals who have self-selected to receive treatment have concerns about medication, it is likely that there are many individuals opting not to receive treatment for themselves or their child due to these concerns. Future research is needed to explore their perceptions to develop effective interventions that could encourage them to seek treatment.

Conclusions

This qualitative evidence synthesis has systematically reviewed and synthesised the available evidence concerning the experiences and beliefs of individuals with FH regarding their condition and its treatment. It has uncovered several enablers and barriers that are to be utilised in clinical practice to facilitate optimal treatment adherence in this high-risk clinical population group. It has also highlighted significant research

For peer review only - http://bmjopen.bmj.com/site/about/guidelines.xhtml

gaps which need to be addressed to gain a more comprehensive understanding of how these individuals can be supported to adhere to lifelong treatment.

Acknowledgements

We would like to thank Catherine Borwick (Research Engagement Librarian) and Dr. Alison Gregory (Research Fellow) at The University of Bristol for their expertise and assistance in the development of this evidence synthesis. We would also like to thank Dr. Paul Downie (Consultant at University Hospitals Bristol and Royal United Hospitals Bath NHS Foundation Trusts) for his input into translating the findings into clinical recommendations.

Author contributions: FJK and RP devised and carried out the search strategy. FJK, RP, FEL and JPHS carried out the study screening and selection stage. FJK and JC carried out the study characteristic extraction stage. FJK and AS carried out the results data extraction, quality appraisal, data analysis and interpretation. EW also carried out the data analysis and interpretation stages. AH contributed to the development and presentation of the qualitative methodology and results. FJK, JPHS and GB translated findings into clinical implications. FJK prepared the manuscript. All authors reviewed the manuscript and approved the final version.

Funding: This evidence synthesis is funded by the National Institute for Health Research NIHR Bristol Biomedical Research Centre (Nutrition theme) at University Hospitals Bristol NHS Foundation Trust and The University of Bristol.

Disclaimer: The views expressed in this publication are those of the authors and not necessarily those of the NHS, the NIHR or the Department of Health and Social Care.

Competing interests: None declared.

Data availability statement

Data are available upon reasonable request. Data extracted from each of the 24 publications have been stored in word document files and are available by emailing the first author.

Abbreviations:

FH: Familial Hypercholesteroleamia

LDL-C: Low density lipoprotein cholesterol

CVD: Cardiovascular disease

CASP: Critical apprisal skills programme

PA: Physical activity

DRAFT

For peer review only

AHA: The American Heart Association

ENTREQ: Enhancing transparency in reporting the synthesis of qualitative research

CHD: Coronary heart disease

SSI: Semi-structured interview

AYA: Adult and young adolescent

HCPs: Healthcare professionals

QOL: Quality of life

Figure Legends

Figure 1: PRISMA Flow diagram

Figure 2: Thematic schema illustrating influence of analytical themes upon treatment adherence

References

1. Akioyamen LE, Genest J, Shan SD, et al. Estimating the prevalence of heterozygous familial hypercholesterolaemia: a systematic review and meta-analysis. *BMJ Open* 2017;7(9)
2. Benn M, Watts GF, Tybjærg-Hansen A, et al. Mutations causative of familial hypercholesterolaemia: screening of 98 098 individuals from the Copenhagen General Population Study estimated a prevalence of 1 in 217. *European heart journal* 2016;37(17):1384-94. doi: 10.1093/eurheartj/ehw028
3. Wong B, Kruse G, Kutikova L, et al. Cardiovascular Disease Risk Associated With Familial Hypercholesterolemia: A Systematic Review of the Literature. *Clinical therapeutics* 2016;38(7):1696-709. doi: 10.1016/j.clinthera.2016.05.006 [published Online First: 2016/06/05]
4. Civeira F. Guidelines for the diagnosis and management of heterozygous familial hypercholesterolemia. *Atherosclerosis* 2004;173(1):55-68. doi: https://doi.org/10.1016/j.atherosclerosis.2003.11.010
5. Humphries SE, Cooper JA, Seed M, et al. Coronary heart disease mortality in treated familial hypercholesterolaemia: Update of the UK Simon Broome FH register. *Atherosclerosis* 2018;274:41-46. doi: https://doi.org/10.1016/j.atherosclerosis.2018.04.040
6. Mohrschladt MF, Westendorp RG, Gevers Leuven JA, et al. Cardiovascular disease and mortality in statin-treated patients with familial hypercholesterolemia. *Atherosclerosis* 2004;172(2):329-35. doi: 10.1016/j.atherosclerosis.2003.11.007 [published Online First: 2004/03/17]
7. Versmissen J, Oosterveer DM, Yazdanpanah M, et al. Efficacy of statins in familial hypercholesterolaemia: a long term cohort study. *BMJ* 2008;337
8. Besseling J, Hovingh GK, Huijgen R, et al. Statins in Familial Hypercholesterolemia: Consequences for Coronary Artery Disease and All-Cause Mortality. *Journal of the American College of Cardiology* 2016;68(3):252-60. doi: 10.1016/j.jacc.2016.04.054 [published Online First: 2016/07/16]
9. Mundal LJ, Igland J, Veierod MB, et al. Impact of age on excess risk of coronary heart disease in patients with familial hypercholesterolaemia. *Heart (British Cardiac Society)* 2018 doi: 10.1136/heartjnl-2017-312706 [published Online First: 2018/04/07]
10. Neil A, Cooper J, Betteridge J, et al. Reductions in all-cause, cancer, and coronary mortality in statin-treated patients with heterozygous familial hypercholesterolaemia: a prospective registry study. *European heart journal* 2008;29(21):2625-33. doi: 10.1093/eurheartj/ehn422 [published Online First: 2008/10/09]
11. Nordestgaard BG, Chapman MJ, Humphries SE, et al. Familial hypercholesterolaemia is underdiagnosed and undertreated in the general population: guidance for clinicians to prevent coronary heart disease : Consensus Statement of the European Atherosclerosis Society. *European heart journal* 2013;34(45):3478-90. doi: 10.1093/eurheartj/eht273
12. Kerr M, Pears R, Miedzybrodzka Z, et al. Cost effectiveness of cascade testing for familial hypercholesterolaemia, based on data from familial hypercholesterolaemia services in the UK. *European heart journal* 2017;38(23):1832-39. doi: 10.1093/eurheartj/ehx111 [published Online First: 2017/04/08]

For peer review only - http://bmjopen.bmj.com/site/about/guidelines.xhtml

13. Louter L, Defesche J, Roeters van Lennep J. Cascade screening for familial hypercholesterolemia: Practical consequences. *Atherosclerosis Supplements* 2017;30:77-85. doi: 10.1016/j.atherosclerosissup.2017.05.019 [published Online First: 2017/11/04]

14. Hartgers ML, Besseling J, Stroes ES, et al. Achieved LDL cholesterol levels in patients with heterozygous familial hypercholesterolemia: A model that explores the efficacy of conventional and novel lipid-lowering therapy. *J Clin Lipidol* 2018 doi: 10.1016/j.jacl.2018.04.002 [published Online First: 2018/06/24]

15. Pijlman AH, Huijgen R, Verhagen SN, et al. Evaluation of cholesterol lowering treatment of patients with familial hypercholesterolemia: a large cross-sectional study in The Netherlands. *Atherosclerosis* 2010;209(1):189-94. doi: https://doi.org/10.1016/j.atherosclerosis.2009.09.014

16. Langslet G, Bogsrud MP, Halvorsen I, et al. Long-term follow-up of young adults with familial hypercholesterolemia after participation in clinical trials during childhood. *Journal of Clinical Lipidology* 2015;9(6):778-85. doi: https://doi.org/10.1016/j.jacl.2015.08.008

17. Gidding SS, Champagne MA, de Ferranti SD, et al. The Agenda for Familial Hypercholesterolemia: A Scientific Statement From the American Heart Association. *Circulation* 2015;132(22):2167-92. doi: 10.1161/cir.0000000000000297 [published Online First: 2015/10/30]

18. Hagger MS, Hardcastle SJ, Hingley C, et al. Predicting Self-Management Behaviors in Familial Hypercholesterolemia Using an Integrated Theoretical Model: the Impact of Beliefs About Illnesses and Beliefs About Behaviors. *International Journal of Behavioral Medicine* 2016;23(3):282-94. doi: 10.1007/s12529-015-9531-x

19. Hagger MS, Hardcastle SJ, Hu M, et al. Effects of medication, treatment, and behavioral beliefs on intentions to take medication in patients with familial hypercholesterolemia. *Atherosclerosis* 2018;277:493-501. doi: https://doi.org/10.1016/j.atherosclerosis.2018.06.010

20. Green J, Britten N. Qualitative research and evidence based medicine. *BMJ : British Medical Journal* 1998;316(7139):1230-32.

21. George M, Freedman TG, Norfleet AL, et al. Qualitative research-enhanced understanding of patients' beliefs: Results of focus groups with low-income, urban, African American adults with asthma. *Journal of Allergy and Clinical Immunology* 2003;111(5):967-73. doi: https://doi.org/10.1067/mai.2003.1459

22. Remien RH, Hirky AE, Johnson MO, et al. Adherence to Medication Treatment: A Qualitative Study of Facilitators and Barriers Among a Diverse Sample of HIV+ Men and Women in Four U.S. Cities. *AIDS and Behavior* 2003;7(1):61-72. doi: 10.1023/A:1022513507669

23. Hardcastle SJ, Legge E, Laundy CS, et al. Patients' Perceptions and Experiences of Familial Hypercholesterolemia, Cascade Genetic Screening and Treatment. *International Journal of Behavioral Medicine* 2015;22(1):92-100. doi: 10.1007/s12529-014-9402-x

24. Frich JC, Malterud K, Fugelli, et al. Experiences of guilt and shame in patients with familial hypercholesterolemia: a qualitative interview study. *Patient Education & Counseling* 2007;69(1-3):108-13.

25. Sliwinski SK, Gooding H, de Ferranti S, et al. Transitioning from pediatric to adult health care with familial hypercholesterolemia: Listening to young adult and parent voices. *Journal of Clinical Lipidology* 2017;11(1):147-59.

26. Weiner K, Durrington PN. Patients' understandings and experiences of familial hypercholesterolemia. *Community genetics* 2008;11(5):273-82. doi: 10.1159/000121398 [published Online First: 2008/05/22]

27. Finfgeld-Connett D. Generalizability and transferability of meta-synthesis research findings. *Journal of Advanced Nursing* 2010;66(2):246-54. doi: 10.1111/j.1365-2648.2009.05250.x

28. Harris JL, Booth A, Cargo M, et al. Cochrane Qualitative and Implementation Methods Group guidance series—paper 2: methods for question formulation, searching, and protocol development for qualitative evidence synthesis. *Journal of Clinical Epidemiology* 2018;97:39-48. doi: https://doi.org/10.1016/j.jclinepi.2017.10.023

29. Sheldon TA. Making evidence synthesis more useful for management and policy-making. *Journal of health services research & policy* 2005;10(1_suppl):1-5. doi: 10.1258/1355819054308521

30. Ring NA, Ritchie K, Mandava L, et al. A guide to synthesising qualitative research for researchers undertaking health technology assessments and systematic reviews, 2011.

31. Wells M, Williams B, Firnigl D, et al. Supporting 'work-related goals' rather than 'return to work' after cancer? A systematic review and meta-synthesis of 25 qualitative studies. *Psycho-oncology* 2013;22(6):1208-19. doi: 10.1002/pon.3148 [published Online First: 2012/08/14]

32. Thomas J, Sutcliffe K, Harden A, et al. Children and Healthy Eating: A Systematic Review of Barriers and Facilitators2003.

For peer review only - http://bmjopen.bmj.com/site/about/guidelines.xhtml

33. Mechta Nielsen T, Frojk Juhl M, Feldt-Rasmussen B, et al. Adherence to medication in patients with chronic kidney disease: a systematic review of qualitative research. *Clinical kidney journal* 2018;11(4):513-27. doi: 10.1093/ckj/sfx140 [published Online First: 2018/08/11]

34. Ma Q, Tso LS, Rich ZC, et al. Barriers and facilitators of interventions for improving antiretroviral therapy adherence: a systematic review of global qualitative evidence. *Journal of the International AIDS Society* 2016;19(1):21166. doi: 10.7448/ias.19.1.21166 [published Online First: 2016/10/21]

35. Munro SA, Lewin SA, Smith HJ, et al. Patient adherence to tuberculosis treatment: a systematic review of qualitative research. *PLoS medicine* 2007;4(7):e238. doi: 10.1371/journal.pmed.0040238 [published Online First: 2007/08/07]

36. Langlois EV, Tuncalp, O., Norris, S.L., Askew, I., and Ghaffar, A. . Qualitative evidence to improve guidelines and health decision-making. *Bulletin of the World Health Organization* 2018 96(79-79A) doi: http://dx.doi.org/10.2471/BLT.17.206540

37. Kinnear FJ, Perry R, Searle A, et al. How do the experiences and beliefs of adults and children with heterozygous familial hypercholesterolaemia influence their adherence to treatment? A systematic review of qualitative evidence protocol. *Syst Rev* 2018;7(1):120. doi: 10.1186/s13643-018-0793-7 [published Online First: 2018/08/17]

38. Tong A, Flemming K, McInnes E, et al. Enhancing transparency in reporting the synthesis of qualitative research: ENTREQ. *BMC medical research methodology* 2012;12(1):181. doi: 10.1186/1471-2288-12-181

39. Programme CAS. CASP Qualitative Research Checklist, 2017.

40. Thomas J, Harden A. Methods for the thematic synthesis of qualitative research in systematic reviews. *BMC medical research methodology* 2008;8(1):45. doi: 10.1186/1471-2288-8-45

41. Noyes J, Booth A, Flemming K, et al. Cochrane Qualitative and Implementation Methods Group guidance series-paper 3: methods for assessing methodological limitations, data extraction and synthesis, and confidence in synthesized qualitative findings. *J Clin Epidemiol* 2018;97:49-58. doi: 10.1016/j.jclinepi.2017.06.020 [published Online First: 2017/12/17]

42. Barnett-Page E, Thomas J. Methods for the synthesis of qualitative research: a critical review. *BMC medical research methodology* 2009;9:59. doi: 10.1186/1471-2288-9-59 [published Online First: 2009/08/13]

43. Carroll C, Booth A. Quality assessment of qualitative evidence for systematic review and synthesis: Is it meaningful, and if so, how should it be performed? *Research Synthesis Methods* 2014;6(2):149-54. doi: 10.1002/jrsm.1128

44. Carroll C, Booth A, Lloyd-Jones M. Should we exclude inadequately reported studies from qualitative systematic reviews? An evaluation of sensitivity analyses in two case study reviews. *Qualitative health research* 2012;22(10):1425-34. doi: 10.1177/1049732312452937 [published Online First: 2012/08/07]

45. Craig JC, Tong A, Strippoli GFM, et al. A guide to reading and using systematic reviews of qualitative research. *Nephrology Dialysis Transplantation* 2014;31(6):897-903. doi: 10.1093/ndt/gfu354 %J Nephrology Dialysis Transplantation

46. Frich JC. Living with genetic risk of coronary heart disease: A qualitative interview study of patients with familial hypercholesterolemia. *Thesis* 2007

47. Urke EB. Young adults with familial hypercholesterolemia and their thoughts on lifelong treatment. A qualitative interview study. . *Master Thesis* 2016

48. Weiner K. Patient and Professional COnstructions of Familial Hypercholesterolaemia and Heart Disease: Testing the Limits of the Geneticisation Thesis. . *PhD Thesis* 2006

49. Agard A, Bolmsjo IA, Hermeren G, et al. Familial hypercholesterolemia: ethical, practical and psychological problems from the perspective of patients. *Patient Educ Couns* 2005;57(2):162-7. doi: 10.1016/j.pec.2004.05.010 [published Online First: 2005/05/25]

50. DeAngelis EJ, McIntosh S, Ahmed CD, et al. Familial hypercholesterolaemia patient–determined themes for community-engaged research. *Health Education Journal* 2017;77(3):293-302. doi: 10.1177/0017896917745567

51. Hallowell N, Jenkins N, Douglas M, et al. A qualitative study of patients' perceptions of the value of molecular diagnosis for familial hypercholesterolemia (FH). *Journal of Community Genetics* 2017;8(1):45-52. doi: 10.1007/s12687-016-0286-0

52. Jenkins N, Lawton J, Douglas M, et al. Inter-embodiment and the experience of genetic testing for familial hypercholesterolaemia. *Sociology of Health and Illness* 2013;35(4):529-43.

For peer review only - http://bmjopen.bmj.com/site/about/guidelines.xhtml

53. Jenkins N, Lawton J, Douglas M, et al. How do index patients participating in genetic screening programmes for familial hypercholesterolemia (FH) interpret their DNA results? A UK-based qualitative interview study. *Patient Education and Counseling* 2013;90(3):372-77. doi: https://doi.org/10.1016/j.pec.2011.09.002
54. Hollands GJ, Armstrong D, Macfarlane A, et al. Patient accounts of diagnostic testing for familial hypercholesterolaemia: comparing responses to genetic and non-genetic testing methods. *BMC Medical Genetics* 2012;13:87-87. doi: 10.1186/1471-2350-13-87
55. Hollman G, Ek A, Olsson AG, et al. The meaning of quality of life among patients with familial hypercholesterolemia. *Journal of Cardiovascular Nursing* 2004;19(4):243-50.
56. Keenan KF, Finnie RM, Simpson WG, et al. Parents' views of genetic testing and treatment of familial hypercholesterolemia in children: a qualitative study. *Journal of Community Genetics* 2018 doi: 10.1007/s12687-018-0373-5
57. Kirkegaard P, Edwards A, Risor MB, et al. Risk of cardiovascular disease? A qualitative study of risk interpretation among patients with high cholesterol. *BMC Fam Pract* 2013;14:137. doi: 10.1186/1471-2296-14-137 [published Online First: 2013/09/18]
58. Mackie TI, Tse LL, de Ferranti SD, et al. Treatment decision making for adolescents with familial hypercholesterolemia: Role of family history and past experiences. *Journal of Clinical Lipidology* 2015;9(4):583-93.e3. doi: https://doi.org/10.1016/j.jacl.2015.04.008
59. Meulenkamp TM, Tibben A, Mollema ED, et al. Predictive genetic testing for cardiovascular diseases: Impact on carrier children. *American Journal of Medical Genetics, Part A* 2008;146(24):3136-46.
60. Mortensen GL, Madsen IB, Kruse C, et al. Familial hypercholesterolaemia reduces the quality of life of patients not reaching treatment targets. *Danish medical journal* 2016;63(5) [published Online First: 2016/04/30]
61. Senior V, Smith JA, Michie S, et al. Making sense of risk: An interpretive phenomenological analysis of vulnerability to heart disease. *Special Issue: Interpretive phenomenological analysis and the new genetics* 2002;7(2):157-68. doi: http://dx.doi.org/10.1177/1359105302007002455
62. Frich JC, Ose L, Malterud K, et al. Perceived vulnerability to heart disease in patients with familial hypercholesterolemia: a qualitative interview study. *Annals of family medicine* 2006;4(3):198-204. doi: 10.1370/afm.529 [published Online First: 2006/06/01]
63. Frich JC, Malterud K, Fugelli P. How do patients at risk portray candidates for coronary heart disease? A qualitative interview study. *Scandinavian Journal of Primary Health Care* 2007;25(2):112-16.
64. Weiner K. The tenacity of the coronary candidate: how people with familial hypercholesterolaemia construct raised cholesterol and coronary heart disease. *Health (London, England : 1997)* 2009;13(4):407-27. doi: 10.1177/1363459309103915 [published Online First: 2009/06/06]
65. Weiner K. Exploring genetic responsibility for the self, family and kin in the case of hereditary raised cholesterol. *Social Science & Medicine* 2011;72(11):1760-67. doi: 10.1016/j.socscimed.2010.03.053
66. Claassen L, Henneman L, Kindt I, et al. Perceived Risk and Representations of Cardiovascular Disease and Preventive Behaviour in People Diagnosed with Familial Hypercholesterolemia: A Cross-sectional Questionnaire Study. *Journal of Health Psychology* 2010;15(1):33-43. doi: 10.1177/1359105309345170
67. van Maarle MC, Stouthard ME, Bonsel GJ. Risk perception of participants in a family-based genetic screening program on familial hypercholesterolemia. *American journal of medical genetics Part A* 2003;116a(2):136-43. doi: 10.1002/ajmg.a.10061 [published Online First: 2002/12/21]
68. Claassen L, Henneman L, van der Weijden T, et al. Being at risk for cardiovascular disease: Perceptions and preventive behavior in people with and without a known genetic predisposition. *Psychology, Health & Medicine* 2012;17(5):511-21. doi: 10.1080/13548506.2011.644246
69. Muir LA, George PM, Whitehead L. Using the experiences of people with familial hypercholesterolaemia to help reduce the risk of cardiovascular disease: a qualitative systematic review. *Journal of Advanced Nursing* 2012;68(9):1920-32. doi: 10.1111/j.1365-2648.2012.05957.x
70. Andersen LK, Jensen HK, Juul S, et al. Patients' attitudes toward detection of heterozygous familial hypercholesterolemia. *Archives of internal medicine* 1997;157(5):553-60. [published Online First: 1997/03/10]
71. Tonstad S, Novik TS, Vandvik IH. Psychosocial function during treatment for familial hypercholesterolemia. *Pediatrics* 1996;98(2 Pt 1):249-55. [published Online First: 1996/08/01]
72. de Jongh S, Kerckhoffs MC, Grootenhuis MA, et al. Quality of life, anxiety and concerns among statin-treated children with familial hypercholesterolaemia and their parents. *Acta Pædiatrica* 2003;92(9):1096-101. doi: 10.1111/j.1651-2227.2003.tb02584.x

For peer review only - http://bmjopen.bmj.com/site/about/guidelines.xhtml

73. van Maarle MC, Stouthard ME, Bonsel GJ. Quality of life in a family based genetic cascade screening programme for familial hypercholesterolaemia: a longitudinal study among participants. *Journal of medical genetics* 2003;40(1):e3. [published Online First: 2003/01/15]
74. van Maarle MC, Stouthard ME, Marang-van de Mheen PJ, et al. How disturbing is it to be approached for a genetic cascade screening programme for familial hypercholesterolaemia? Psychological impact and screenees' views. *Community genetics* 2001;4(4):244-52. doi: 10.1159/000064200 [published Online First: 2003/05/20]
75. Hyttinen L, Kekäläinen P, Vuorio AF, et al. Health-related quality of life in elderly patients with familial hypercholesterolemia. *International Journal of Technology Assessment in Health Care* 2008;24(2):228-34. doi: 10.1017/S0266462308080318
76. Akioyamen LE, Genest J, Shan SD, et al. Anxiety, depression, and health-related quality of life in heterozygous familial hypercholesterolemia: A systematic review and meta-analysis. *Journal of psychosomatic research* 2018;109:32-43. doi: 10.1016/j.jpsychores.2018.03.170 [published Online First: 2018/05/19]
77. Senior V, Marteau TM, Weinman J. Self-Reported Adherence to Cholesterol-Lowering Medication in Patients with Familial Hypercholesterolaemia: The Role of Illness Perceptions. *Cardiovascular Drugs and Therapy* 2004;18(6):475-81. doi: 10.1007/s10557-004-6225-z
78. Galema-Boers A, Langendonk JG, Roeters Van Lennep J, et al. Developing a prediction model to identify noncompliance in a nurse-led familial hypercholesterolemia clinic. *European Heart Journal* 2011;1):66.
79. Galema-Boers JM, Lenzen MJ, van Domburg RT, et al. Predicting non-adherence in patients with familial hypercholesterolemia. *European journal of clinical pharmacology* 2014;70(4):391-7. doi: 10.1007/s00228-013-1640-3 [published Online First: 2014/01/23]
80. Braamskamp MJAM, Kusters DM, Avis HJ, et al. Long-Term Statin Treatment in Children with Familial Hypercholesterolemia: More Insight into Tolerability and Adherence. *Paediatric Drugs* 2015;17(2):159-66. doi: 10.1007/s40272-014-0116-y
81. Retterstol K, Mundal L, Igland J, et al. Incidence of various types of atherosclerotic disease in patients with genotyped familial hypercholesterolemia. *Atherosclerosis* 2017;263:e26. doi: https://doi.org/10.1016/j.atherosclerosis.2017.06.106
82. Hopkins PN. Putting Into Perspective the Hazards of Untreated Familial Hypercholesterolemia. *Journal of the American Heart Association* 2017;6(6)
83. deGoma EM, Ahmad ZS, O'Brien EC, et al. Treatment Gaps in Adults With Heterozygous Familial Hypercholesterolemia in the United States: Data From the CASCADE-FH Registry. *Circulation Cardiovascular genetics* 2016;9(3):240-9. doi: 10.1161/circgenetics.116.001381 [published Online First: 2016/03/26]
84. Saltijeral A, Perez de Isla L, Alonso R, et al. Attainment of LDL Cholesterol Treatment Goals in Children and Adolescents With Familial Hypercholesterolemia. The SAFEHEART Follow-up Registry. *Revista espanola de cardiologia (English ed)* 2017;70(6):444-50. doi: 10.1016/j.rec.2016.10.010 [published Online First: 2016/12/04]
85. Humphries SE, Cooper J, Dale P, et al. The UK Paediatric Familial Hypercholesterolaemia Register: Statin-related safety and 1-year growth data. *J Clin Lipidol* 2018;12(1):25-32. doi: 10.1016/j.jacl.2017.11.005 [published Online First: 2017/12/07]
86. Galema-Boers AM, Lenzen MJ, Engelkes SR, et al. Cardiovascular risk in patients with familial hypercholesterolemia using optimal lipid-lowering therapy. *Journal of Clinical Lipidology* 2018;12(2):409-16. doi: https://doi.org/10.1016/j.jacl.2017.12.014
87. Pérez de Isla L, Alonso R, Mata N, et al. Predicting Cardiovascular Events in Familial Hypercholesterolemia. *Circulation* 2017;135(22):2133.
88. Senior V, Marteau T, Weinman J. Perceptions of control over heart disease in people with an inherited predisposition to raised cholesterol. *Psychology, Health & Medicine* 2005;10(1):16-30. doi: 10.1080/13548500512331315334
89. Senior V, Marteau TM. Causal attributions for raised cholesterol and perceptions of effective risk-reduction: Self-regulation strategies for an increased risk of coronary heart disease. *Psychology and Health* 2007;22(6):699-717. doi: 10.1080/14768320601020253
90. Tonstad S. Familial hypercholesterolaemia: a pilot study of parents' and children's concerns. *Acta paediatrica (Oslo, Norway : 1992)* 1996;85(11):1307-13. [published Online First: 1996/11/01]

For peer review only - http://bmjopen.bmj.com/site/about/guidelines.xhtml

91. Avis HJ, Kusters DM, Vissers MN, et al. Follow-Up of Children Diagnosed with Familial Hypercholesterolemia in a National Genetic Screening Program. *The Journal of Pediatrics* 2012;161(1):99-103. doi: https://doi.org/10.1016/j.jpeds.2011.12.037
92. Benson G, Witt DR, VanWormer JJ, et al. Medication adherence, cascade screening, and lifestyle patterns among women with hypercholesterolemia: Results from the WomenHeart survey. *J Clin Lipidol* 2016;10(4):937-43. doi: 10.1016/j.jacl.2016.03.012 [published Online First: 2016/09/01]
93. Collins R, Reith C, Emberson J, et al. Interpretation of the evidence for the efficacy and safety of statin therapy. *The Lancet* 2016;388(10059):2532-61. doi: 10.1016/S0140-6736(16)31357-5
94. Vuorio A, Kuoppala J, Kovanen PT, et al. Statins for children with familial hypercholesterolemia. *Cochrane Database Syst Rev* 2017;7:Cd006401. doi: 10.1002/14651858.CD006401.pub4 [published Online First: 2017/07/08]
95. Lozano P, Henrikson NB, Morrison CC, et al. Lipid Screening in Childhood and Adolescence for Detection of Multifactorial Dyslipidemia: Evidence Report and Systematic Review for the US Preventive Services Task Force. *Jama* 2016;316(6):634-44. doi: 10.1001/jama.2016.6423 [published Online First: 2016/08/18]
96. Radaelli G, Sausen G, Cesa CC, et al. Statin Treatments And Dosages In Children With Familial Hypercholesterolemia: Meta-Analysis. *Arquivos brasileiros de cardiologia* 2018 doi: 10.5935/abc.20180180 [published Online First: 2018/10/27]
97. Hollman G, Olsson AG, Ek AC. Disease knowledge and adherence to treatment in patients with familial hypercholesterolemia. *The Journal of cardiovascular nursing* 2006;21(2):103-8. [published Online First: 2006/04/08]
98. van Maarle MC, Stouthard MEA, de Mheen PJM-v, et al. Follow up after a family based genetic screening programme for familial hypercholesterolaemia: is screening alone enough? *BMJ : British Medical Journal* 2002;324(7350):1367-68.
99. Kinnear FJ, Lithander FE, Hamilton-Shield JP, et al. What dietetic support is available to patients in outpatient lipid clinics in the uk? *Atherosclerosis Supplements* 2018;34:e5. doi: 10.1016/j.atherosclerosissup.2018.07.029
100. Umans-Eckenhausen MA, Defesche JC, van Dam MJ, et al. Long-term compliance with lipid-lowering medication after genetic screening for familial hypercholesterolemia. *Archives of internal medicine* 2003;163(1):65-8. [published Online First: 2003/01/14]
101. Jones LK, Kulchak Rahm A, Manickam K, et al. Healthcare Utilization and Patients' Perspectives After Receiving a Positive Genetic Test for Familial Hypercholesterolemia. *Circulation Genomic and precision medicine* 2018;11(8):e002146. doi: 10.1161/circgen.118.002146 [published Online First: 2018/10/26]
102. Chisnell J, Marshall T, Hyde C, et al. A content analysis of the representation of statins in the British newsprint media. *BMJ Open* 2017;7(8)
103. Tonstad S, Sivertsen M. Food dislikes and dietary intake in children with familial hypercholesterolemia and controls. *Scandinavian Journal of Nutrition/Naringsforskning* 1996;40(4):146-50.
104. Stender S, Skovby F, Haraldsdottir J, et al. Cholesterol-lowering diets may increase the food costs for Danish children. A cross-sectional study of food costs for Danish children with and without familial hypercholesterolaemia. *European Journal of Clinical Nutrition* 1993;47(11):776-86.
105. Dixon LB, Tershakovec AM, McKenzie J, et al. Diet quality of young children who received nutrition education promoting lower dietary fat. *Public health nutrition* 2000;3(4):411-6. [published Online First: 2001/01/03]
106. Molven I, Retterstol K, Andersen LF, et al. Children and young adults with familial hypercholesterolaemia (FH) have healthier food choices particularly with respect to dietary fat sources compared with non-FH children. *Journal of nutritional science* 2013;2:e32. doi: https://dx.doi.org/10.1017/jns.2013.27
107. West RJ. FAMILIAL HYPERCHOLESTEROLAEMIA. FACTORS INFLUENCING DRUG COMPLIANCE. *Pediatric Research* 1980;14:1423. doi: 10.1203/00006450-198012000-00089
108. Marteau T, Senior V, Humphries SE, et al. Psychological impact of genetic testing for familial hypercholesterolemia within a previously aware population: a randomized controlled trial. *American journal of medical genetics Part A* 2004;128a(3):285-93. doi: 10.1002/ajmg.a.30102 [published Online First: 2004/06/25]
109. Lee S, Akioyamen LE, Aljenedil S, et al. Genetic testing for familial hypercholesterolemia: Impact on diagnosis, treatment and cardiovascular risk. *Eur J Prev Cardiol* 2019:2047487319829746. doi: 10.1177/2047487319829746 [published Online First: 2019/02/14]

For peer review only - http://bmjopen.bmj.com/site/about/guidelines.xhtml

110. Ramli A, Ahmad NS, Paraidathathu T. Medication adherence among hypertensive patients of primary health clinics in Malaysia. *Patient preference and adherence* 2012;6:613-22. doi: 10.2147/PPA.S34704
111. Polonsky WH, Henry RR. Poor medication adherence in type 2 diabetes: recognizing the scope of the problem and its key contributors. *Patient preference and adherence* 2016;10:1299-307. doi: 10.2147/PPA.S106821

For peer review only
DRAFT

Figure 1: PRISMA Flow diagram

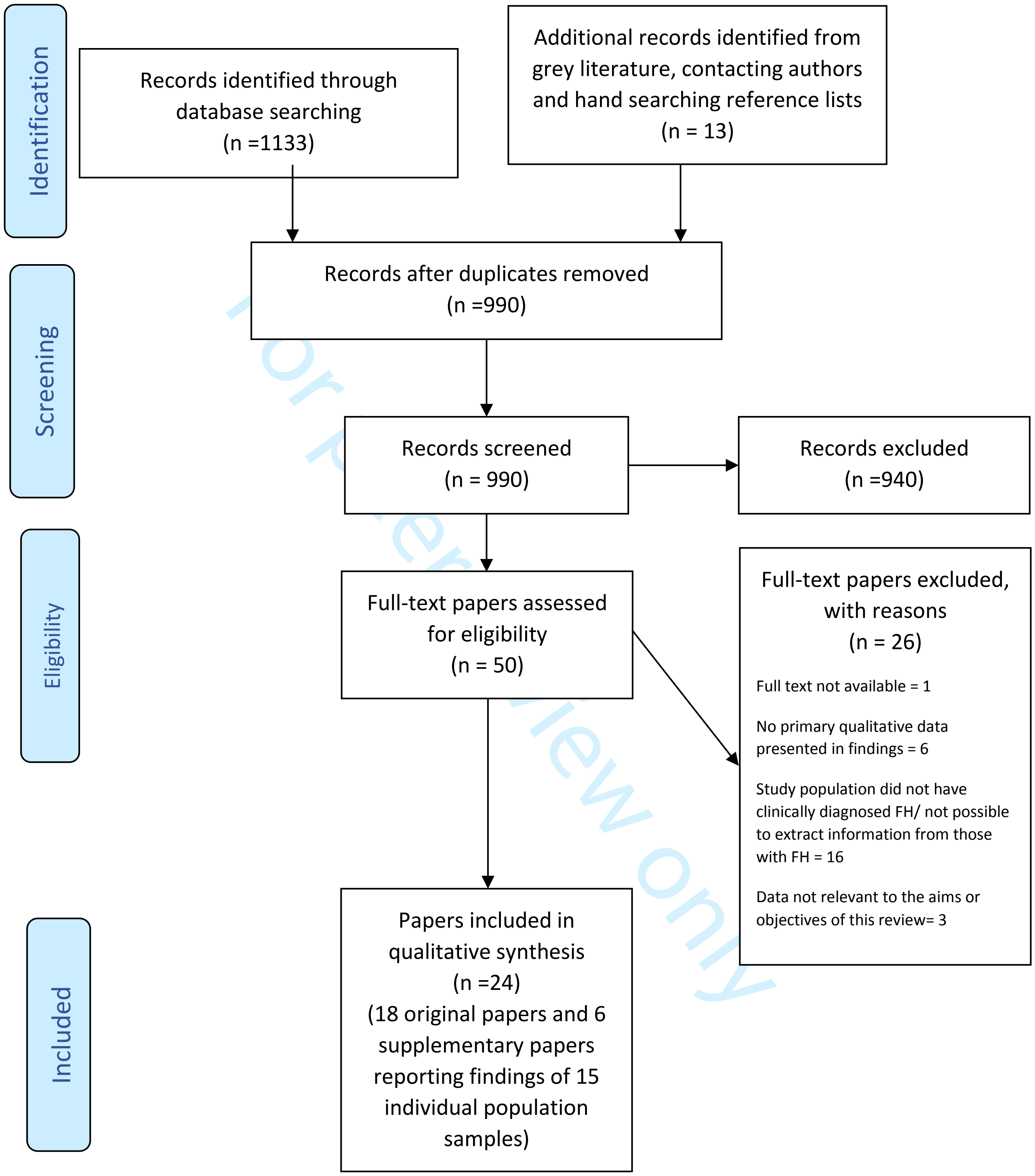

For peer review only

Figure 2: Thematic schema illustrating influence of analytical themes upon treatment adherence

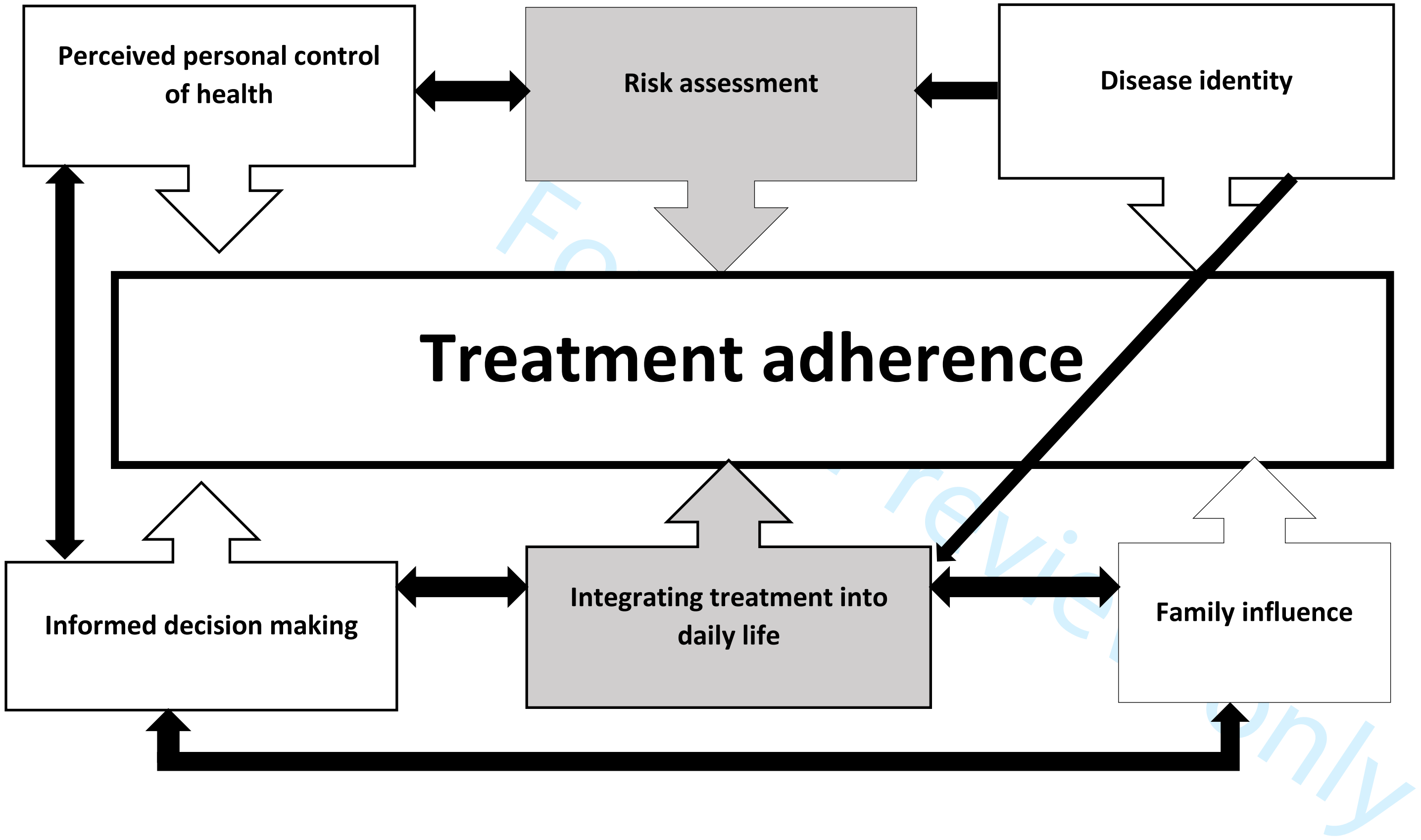

Supplementary File 1: Deviations from protocol

Stated in protocol	What we did	Rationale for deviation
'Only studies in which the full text is available in English will be eligible for inclusion'	We did not place any limits upon language of included papers	We aimed to overcome the recognised restrictions of individual qualitative study findings, by gathering and examining a wide range of patient perceptions and experiences. After an initial scope of the available evidence base in this population group, it was apparent that the number of potential papers to be retrieved would be manageable by the research team. Therefore, the decision was made to remove this exclusion criteria, in order to identify all relevant evidence in line with the comprehensive searching approach to be taken in this review. This is in line with available guidance which advises that language filter decisions should be made in reference to the aims of the review.[28]
'The participants include individuals aged ≥ 10 years'	We did not place any limits upon age of included participants.	The database searching retrieved a paper reporting findings from a sample which included children aged 8 years. As it was not possible to extract the data from only participants aged 10 years and older, using the original inclusion criteria the paper would have to be excluded from the synthesis. This paper was one of only 3 papers retrieved that reported findings from samples including children, therefore the findings were perceived to be very valuable to the synthesis. Children are often diagnosed with FH before the age of 10, and U.K. and international guidance advise treatment with lifestyle advice, with lipid lowering therapy to be implemented when they reach a suitable age.[11] Furthermore, it is stated in the NICE guidelines that lipid lowering drug treatment should be commenced by the age of 10 and statin therapy can be considered at 8-10 years of age.
'Both stages of data extraction will be carried out independently by two reviewers (AS, FJK)…'	First stage of data extraction (study details) was carried out by two reviewers (JC, FJK) and second stage (study findings) by two reviewers (AS, FJK).	This was to split work between review members.
'The two reviewers (FJK, AS) will then work in collaboration to develop initial descriptive themes and categories based upon the raw data…'	Three reviewers (FJK, AS, EW) worked in collaboration for the second two stages of thematic synthesis.	EW joined review team after publication of protocol. We felt having a further expert opinion from a health psychologist would improve the synthesis output.
'The findings are intended to be used in the development of future intervention or guidelines….'	The findings are presented with a focus on informing clinical practice	The findings were interpreted to be of particular importance to clinical practice. While the findings are still useful to intervention and guideline development, this paper will focus upon their application in a clinical setting,

For peer review only - http://bmjopen.bmj.com/site/about/guidelines.xhtml

Supplementary File 2- ENTREQ reporting guidelines checklist

ENTREQ: Enhancing transparency in reporting the synthesis of qualitative research [38]

No	Item	Guide and description	Reported on page #
**1**	Aim	State the research question the synthesis addresses.	Page 4 & 5
**2**	Synthesis methodology	Identify the synthesis methodology or theoretical framework which underpins the synthesis, and describe the rationale for choice of methodology *(e.g. meta-ethnography, thematic synthesis, critical interpretive synthesis, grounded theory synthesis, realist synthesis, meta-aggregation, meta-study, framework synthesis).*	Protocol and page 7
**3**	Approach to searching	Indicate whether the search was pre-planned (*comprehensive search strategies to seek all available studies)* or iterative (*to seek all available concepts until they theoretical saturation is achieved).*	Protocol, page 6 and supplementary file 3
**4**	Inclusion criteria	Specify the inclusion/exclusion criteria *(e.g. in terms of population, language, year limits, type of publication, study type).*	Protocol and page 6
**5**	Data sources	Describe the information sources used (e.g. *electronic databases (MEDLINE, EMBASE, CINAHL, psycINFO, Econlit), grey literature databases (digital thesis, policy reports), relevant organisational websites, experts, information specialists, generic web searches (Google Scholar) hand searching, reference lists)* and when the searches conducted; provide the rationale for using the data sources.	Protocol, page 6 and supplementary file 3
**6**	Electronic Search strategy	Describe the literature search *(e.g. provide electronic search strategies with population terms, clinical or health topic terms, experiential or social phenomena related terms, filters for qualitative research, and search limits).*	Protocol, page 6 and supplementary file 3
**7**	Study screening methods	Describe the process of study screening and sifting *(e.g. title, abstract and full text review,*	Protocol and page 7

No	Item	Guide and description	Reported on page #
		number of independent reviewers who screened studies).	
**8**	Study characteristics	Present the characteristics of the included studies *(e.g. year of publication, country, population, number of participants, data collection, methodology, analysis, research questions).*	Page 8, table 1 and supplementary file 4
**9**	Study selection results	Identify the number of studies screened and provide reasons for study exclusion *(e,g, for comprehensive searching, provide numbers of studies screened and reasons for exclusion indicated in a figure/flowchart; for iterative searching describe reasons for study exclusion and inclusion based on modifications t the research question and/or contribution to theory development).*	Figure 1 and page 8
**10**	Rationale for appraisal	Describe the rationale and approach used to appraise the included studies or selected findings *(e.g. assessment of conduct (validity and robustness), assessment of reporting (transparency), assessment of content and utility of the findings).*	Protocol, pages 6-7 and supplementary file 4
**11**	Appraisal items	State the tools, frameworks and criteria used to appraise the studies or selected findings *(e.g. Existing tools: CASP, QARI, COREQ, Mays and Pope*[25]*; reviewer developed tools; describe the domains assessed: research team, study design, data analysis and interpretations, reporting).*	Protocol, pages 6 and supplementary file 4
**12**	Appraisal process	Indicate whether the appraisal was conducted independently by more than one reviewer and if consensus was required.	Protocol, pages 6-7, supplementary file 4
**13**	Appraisal results	Present results of the quality assessment and indicate which articles, if any, were weighted/excluded based on the assessment and give the rationale.	Page 12, Table 1 & supplementary file 4

For peer review only - http://bmjopen.bmj.com/site/about/guidelines.xhtml

No	Item	Guide and description	Reported on page #
**14**	Data extraction	Indicate which sections of the primary studies were analysed and how were the data extracted from the primary studies? *(e.g. all text under the headings "results /conclusions" were extracted electronically and entered into a computer software).*	Protocol and page 7
**15**	Software	State the computer software used, if any.	Protocol and page 7
**16**	Number of reviewers	Identify who was involved in coding and analysis.	Page 7 and supplementary file 5
**17**	Coding	Describe the process for coding of data *(e.g. line by line coding to search for concepts).*	Page 7 and supplementary file 5
**18**	Study comparison	Describe how were comparisons made within and across studies *(e.g. subsequent studies were coded into pre-existing concepts, and new concepts were created when deemed necessary).*	Supplementary file 5
**19**	Derivation of themes	Explain whether the process of deriving the themes or constructs was inductive or deductive.	Supplementary file 5
**20**	Quotations	Provide quotations from the primary studies to illustrate themes/constructs, and identify whether the quotations were participant quotations of the author's interpretation.	Table 2 and pages 13-16
**21**	Synthesis output	Present rich, compelling and useful results that go beyond a summary of the primary studies (e.g. *new interpretation, models of evidence, conceptual models, analytical framework, development of a new theory or construct).*	Table 4 and pages 22-25

3

For peer review only - http://bmjopen.bmj.com/site/about/guidelines.xhtml

Supplementary File 3- Full details of search strategy

Full details are available in the published protocol[37] but are detailed briefly below.

Search Strategy

MEDLINE, Embase, PsycINFO (via OVID), Cochrane library and CINAHL databases were searched from inception to 05/09/2018. We used a validated qualitative search filter[(121)] and population specific search terms. The search strategy that was used in MEDLINE is displayed in Appendix 1. The OpenGrey database and specialist websites (HEART UK, British Heart Foundation, The FH Foundation and The Simon Broome Register) were also searched up until 05/09/2018. The reference lists of the 50 papers taken to the full text screening stage were also hand searched. When only an abstract was available, the lead author was conducted in attempt to retrieve the full text. When contacting the lead authors of the included papers as part of the quality appraisal stage, enquiries were also made about any unpublished work.

Appendix 1: Search Strategy used in MEDLINE 05/09/2018

1. (familial adj1 hypercholesterolemia).ti,ab, kf.

2. (familial adj1 hypercholesterolaemia).ti,ab, kf.

3. (inherit* adj1 high adj1 cholesterol).ti,ab, kf.

4. *Hypercholesterolemia/ge [Genetics]

5. 1 or 2 or 3 or 4

6. interview*.ti,ab.

7. exp. Interviews/

8. experience*.tw.

9. qualitative.ti,ab.

10. 6 or 7 or 8 or 9

11. 5 and 10

For peer review only - http://bmjopen.bmj.com/site/about/guidelines.xhtml

Supplementary File 4- Quality appraisal methodology and results

Methodology

The CASP tool, endorsed by the Cochrane Collaboration,[41] asks 10 questions relating to the rigour of the methodology used, quality of reporting and relevance of findings. To ensure comprehensive evaluation of methodological quality, these questions were answered with further consideration of 12 criteria produced by an expert panel.[112] As the purpose of the quality appraisal was to determine the methodological strengths and limitations of studies included in the synthesis, the lead authors of each paper were contacted to obtain further information in an attempt to overcome the recognised issued of poor reporting in qualitative research. Information from multiple papers involving the same sample was pooled when appropriate. Each author was given 1 month to respond. Two reviewers (AS, FK) independently appraised each study, assigning a rating of 0, 1 or 2 for each question which reflected the extent to which the obtained information from paper and author answered the criteria (0=not addressed, 1=partially addressed, 2=fully addressed). The reviewers then met to come to a consensus of individual and total scores, resolving differences through discussion. The reviewers then decided upon threshold for low, medium and high rated quality that they felt adequately captured the quality of the included papers.

Summary of results

Table 1 displays the CASP score breakdowns for each paper. Table 2 displays further details of the methodological limitations and transferability considerations of each included paper.

For peer review only - http://bmjopen.bmj.com/site/about/guidelines.xhtml

Table 1: CASP appraisal scores of included studies

Sample number	Reference	Was there a clear statement of research aims?	Is qualitative methodology appropriate?	Was the research design appropriate to address the aims of the research?	Was the recruitment strategy appropriate to the aims of the research?	Were the data collected in a way that addressed the research issue?	Has the relationship between researcher and participants been adequately considered?	Have ethical issues been taken into consideration?	Was the data analysis sufficiently rigorous?	Is there a clear statement of findings?	Is the research valuable?	Overall score (out of 20)	Did author provide further information?
**1**	Agard et al, 2005[49]	2	2	2	2	2	0	1	0	1	1	**13**	**NO**
**2**	DeAngelis et al, 2017[50]	2	2	2	1	1	1	0	1	1	2	**13**	**NO**
**3**	Frich, 2007[46]	2	2	2	2	2	2	2	2	2	2	**20**	**YES**
	Frich et al, 2006[62]	2	2	2	2	2	2	2	2	1	2	**19**	**YES**
	Frich et al, 2007[63]	2	2	2	1	2	2	2	2	2	1	**18**	**YES**
	Frich et al, 2007[24]	2	2	2	2	2	2	2	2	2	2	**20**	**YES**
**4**	Hallowell et al, 2017[51]	2	2	2	2	2	1	2	1	1	2	**17**	**YES**
	Jenkins et al, 2013[53]	2	2	2	1	2	0	2	1	1	2	**15**	**YES**
	Jenkins et al, 2013[52]	2	2	2	1	2	0	2	1	0	1	**13**	**YES**
**5**	Hardcastle et al, 2015[23]	2	2	2	2	2	1	2	1	2	2	**18**	**YES**
**6**	Hollands et al, 2012[54]	2	2	2	2	0	0	2	1	1	1	**13**	**NO**
**7**	Hollman et al, 2004[55]	2	2	2	2	2	1	2	2	2	2	**19**	**YES**
**8**	Keenan et al, 2018[56]	2	2	2	2	2	0	2	1	1	2	**16**	**YES**
**9**	Kirkegaard et al, 2014[57]	2	2	2	2	2	1	2	2	1	1	**17**	**YES**

For peer review only - http://bmjopen.bmj.com/site/about/guidelines.xhtml

**10**	Mackie et al, 2015[58]	2	2	2	2	2	2	1	2	2	2	**19**	**NO**
	Sliwinski et al, 2017[25]	2	2	2	2	2	2	1	2	2	2	**19**	**NO**
**11**	Meulenkamp et al, 2008[59]	2	2	2	2	2	1	2	2	2	2	**19**	**YES**
**12**	Mortensen et al, 2008[60]	2	2	2	1	1	0	0	1	1	1	**11**	**NO**
**13**	Urke, 2016[47]	2	2	2	2	2	2	2	2	2	2	**20**	**NO**
**14**	Weiner, 2006[48]	2	2	2	1	2	1	2	1	2	2	**18**	**YES**
	Weiner and Durrington, 2008[26]	2	2	2	1	2	1	2	1	2	2	**17**	**YES**
	Weiner, 2009[64]	2	2	2	1	2	1	2	1	1	1	**16**	**YES**
	Weiner, 2011[65]	2	2	2	1	2	1	2	1	1	2	**16**	**YES**
**15**	Senior et al, 2002[61]	2	2	2	2	1	0	0	1	1	1	**12**	**NO**

Scoring system: 0=No criteria fulfilled or can't tell; 1= some criteria fulfilled; 2= All criteria fulfilled. In reference to the criteria suggested for each question by CASP tool(53) and further criteria as described by Santiago-Delefosse et al.(122)

For peer review only - http://bmjopen.bmj.com/site/about/guidelines.xhtml

Table 2: Summary of methodological limitations and transferability considerations of the included papers

Sample number	Reference	CASP quality SCORE & rating	Methodological and reporting limitations	Transferability considerations of sample
**1**	Agard et al, 2005[49]	13 Low	Lack of details provided about the rigour of the analysis process.<br>Authors self-selected the data from interviews to transcribe.<br>Data saturation not discussed.<br>Relationship between researcher and participants was not adequately considered.<br>Credibility of findings and the limitations of study design not addressed when reporting the findings.<br>No details of informed consent or if participants were told about data confidentiality or their right to withdraw.	No sampling strategy used but sample comprised of a good range of ages, genders, history of CVD events and age of diagnosis.<br>All recruited from one clinic.<br>All from Sweden.
**2**	DeAngelis et al, 2017[50]	13 Low	Ethical issues not addressed.<br>Group meetings may have resulted in lack of representative findings as certain individuals may have dominated the conversations or individuals may have felt unable to voice their own opinions.<br>Lack of disconfirming cases presented<br>No details of informed consent or if participants were told about data confidentiality or their right to withdraw.	All very motivated and engaged individuals to volunteer for this group.<br>Many receiving apheresis treatment.
**3**	Frich, 2007[46]	20 high	Study limitations not addressed when reporting the findings.	All motivated to seek treatment as active attendees of lipid clinic.<br>Majority young (70% 10-39 years) and asymptomatic.<br>Large (40) sample size.<br>All from Norway.<br>All recruited from one lipid clinic.
	Frich et al, 2006[62]	19 High	Lack of disconfirming cases presented and discussion against the findings.	
	Frich et al, 2007[63]	18 High	Lack of disconfirming cases presented and discussion against the findings.	
	Frich et al, 2007[24]	20 High	Study limitations not addressed when reporting the findings.	
**4**	Hallowell et al, 2017[51]	17 Medium	Lack of details provided about the rigour of the analysis process<br>Relationship between researcher and participants was not adequately considered	All participants regularly attend lipid clinics and opted in for DNA testing.<br>Relatively well education (42% university education).<br>All participants from Scotland.<br>No sampling strategy used so likely not representative.<br>Half of patients from professional/skilled non-manual background.<br>Ethnicity not provided but authors state majority white British.<br>Recruited from two lipid clinics.
	Jenkins et al, 2013[53]	15 Medium	Credibility of findings and the limitations of study design not addressed when reporting the findings<br>Relationship between researcher and participants was not adequately considered.	
	Jenkins et al, 2013[52]	13 Low	Lack of details provided about the rigour of the analysis process.<br>Credibility of findings and the limitations of study design not addressed when reporting the findings.<br>Relationship between researcher and participants was not adequately considered.	
**5**	Hardcastle et al, 2015[23]	18 High	Analysis carried out by one individual only with no independent verification of themes conducted.<br>Relationship between researcher and participants was not adequately considered.	Sample not randomly selected.<br>Recruited from one clinic.<br>All live in metropolitan Perth, Australia.
**6**	Hollands et al, 2012[54]	13 Low	Lack of disconfirming cases presented and arguments against findings.<br>Data saturation not discussed.<br>Relationship between researcher and participants was not adequately considered.<br>Credibility of findings and the limitations of study design not addressed when reporting the findings.	Recruited across 11 lipid clinics.<br>All from the U.K.<br>All recently identified as being at risk of FH and during study received either clinical or DNA test results.

For peer review only - http://bmjopen.bmj.com/site/about/guidelines.xhtml

				Sample included in analysis includes participates with DNA positive and Non-DNA positive diagnosis.<br>Majority (14/19) white British.
**7**	Hollman et al, 2004[55]	19 High	Relationship between researcher and participants was not adequately considered.	All Swedish.<br>All recruited from one lipid clinic.
**8**	Keenan et al, 2018[56]	16 Medium	Credibility of findings and the limitations of study design not addressed when reporting the findings.<br>Relationship between researcher and participants was not adequately considered.<br>Analysis carried out by one individual.	All had consented to genetic testing.<br>All from Scotland.<br>All participants white, and majority highly educated.<br>Majority of participants asymptomatic.<br>Patients were self-selected from HCP who excluded participants if they felt they were too vulnerable, which included if had experienced a recent bereavement.<br>13 of parents had FH, 4 were spouses of those with FH
**9**	Kirkegaard et al, 2014[57]	17 Medium	Lack of results to support conclusions drawn.<br>Credibility of findings and the limitations of study design not addressed when reporting the findings.	All asymptomatic.<br>Only 2 FH patients and 1 relative of FH patient.
**10**	Mackie et al, 2015[58]	19 High	No details of informed consent or if participants were told about data confidentiality or their right to withdraw.	Most participants had private medical insurance, were white and all actively engaged with the healthcare system.<br>All recruited from same healthcare system.<br>All patients from Massachusetts, U.S.A.
	Sliwinski et al, 2017[25]	19 High	No details of informed consent or if participants were told about data confidentiality or their right to withdraw.	
**11**	Meulenkamp et al, 2008[59]	19 High	Relationship between researcher and participants was not adequately considered-three interviewers carried out the interviews and the potential bias this may incur was not addressed	All recruited from one health intuition.<br>All engaged with healthcare system and willing to talk about their condition.<br>11/16 were females.
**12**	Mortensen et al, 2008[60]	11 Low	Lack of details provided about the study methodology or rigour of analysis process.<br>Data saturation not discussed.<br>Relationship between researcher and participants was not adequately considered.<br>Credibility of findings and the limitations of study design not addressed when reporting the findings.<br>Ethical issues not addressed.	Half participants were reaching treatment goals, half were not.<br>All recruited from one genetic centre.<br>All Danish.<br>Only 1 female in the group of patients reaching treatment targets
**13**	Urke, 2016[47]	20 High	Coding and analysis of data was primarily independent, with the student's supervisors only overseeing it.	Sample comprised of non-attenders at clinic-not been seen for at least 2 years<br>Wide geographical spread, but all participants from Norway<br>Participants recruited from one clinic
**14**	Weiner, 2006[48]	18 High	Analysis by single researcher and potential bias not addressed<br>Data saturation not discussed.<br>Relationship between researcher and participants was not adequately considered.	Quota sampling used but all were white and majority (28/31) white British, 65% were ≥46 years old and 50% from professional occupations.<br>Participants recruited from one clinic.<br>Half self-reported experiencing some form of CHD.<br>All from North England, U.K.<br>All attended lipid clinic for at least 1 year, most for substantially longer.
	Weiner and Durrington, 2008[26]	17 Medium	Analysis by single researcher and potential bias not addressed.<br>Data saturation not discussed.<br>Relationship between researcher and participants was not adequately considered.<br>Credibility of findings not addressed when reporting the findings.	
	Weiner, 2009[64]	16 Medium	Analysis by single researcher and potential bias not addressed.<br>Data saturation not discussed.<br>Relationship between researcher and participants was not adequately considered.<br>Credibility of findings not addressed when reporting the findings.	
	Weiner, 2011[65]	16 Medium	Analysis by single researcher and potential bias not addressed.<br>Data saturation not discussed.<br>Relationship between researcher and participants was not adequately considered.<br>Credibility of findings not addressed when reporting the findings.	

For peer review only - http://bmjopen.bmj.com/site/about/guidelines.xhtml

**15**	Senior et al, 2002[61]	12 Low	Lack of details provided about the study methodology or rigour of analysis process.<br>Ethical issues not addressed.<br>Data saturation not discussed.<br>Credibility of findings or limitations of study methodology not addressed when reporting findings.<br>Relationship between researcher and participants was not adequately considered.	All motivated to participate in research as recruited from ongoing trial.<br>All lived in central London.<br>All clinical diagnosis, but 5 had DNA diagnosis confirmed and 2 had negative DNA test.

Additional references (To those listed in main manuscript)

112. Santiago-Delefosse M, Gavin A, Bruchez C, Roux P, Stephen SL. Quality of qualitative research in the health sciences: Analysis of the common criteria present in 58 assessment guidelines by expert users. Social Science & Medicine. 2016;148:142-5

DRAFT
For peer review only

For peer review only - http://bmjopen.bmj.com/site/about/guidelines.xhtml

Supplementary File 5: Full details of thematic synthesis methodology

Stage 1: Line by line coding

In our protocol we originally planned to analyse the extracted data according to our review questions regarding factors influencing adherence to treatment. However, few studies directly addressed this question, therefore the authors put these review questions to one side for the data extraction process and revisited them for the coding stage.

Two reviewers (AS, FJK) had previously read all papers independently for the critical appraisal stage. They were therefore familiar with the papers and had discussed them. At this stage they independently reread and coded, on paper, the extracted data from seven papers. The process involved line by line coding of the extracted data in which each line of text was assigned a free code according to its meaning and content. The codes were inductively created in response to the findings uncovered. The two reviewers then met to discuss and compare their findings before then deciding upon a preliminary coding frame which they then used when coding the extracted data from three further papers independently. In addition, new codes were created when necessary and the reviewers met again to discuss the findings, making revisions to the coding frame. One reviewer (FJK) then independently coded the extracted data from each paper using NVivo software. The coding frame was modified and added to throughout this process, with any changes made discussed with a second reviewer (AS). By this stage, no new codes were being identified, but some codes were consolidated into one code and others given more clarification about their meaning. A copy of the finalised coding frame is available to view (Appendix 1). A second reviewer (AS) performed secondary coding on 10% of the papers (three papers) before meeting with FJK to compare findings and ensure consistency of interpretation.

This line by line coding facilitated the translation of concepts from one study to another- a key component of qualitative synthesis. Most sentences were categorised using more than one code as a result of having content which had more than one possible meaning e.g. ‘perceived risk’ and ‘relative risk’ or ‘perceived seriousness’ and ‘emotional impact’.

Stage 2: Development of descriptive themes

This stage involves the development of initial descriptive themes based upon the raw data that closely reflect the aggregative findings of the included studies.[40] The two reviewers who had carried out the coding (AS, FJK) met with a third reviewer (EW) to discuss the findings

For peer review only - http://bmjopen.bmj.com/site/about/guidelines.xhtml

of the coding process. One reviewer (FJK) produced summary reports of each of the 19 identified free codes which provided an overview of the findings across the papers including illustrative quotes and disconfirming cases. The summaries were descriptive in nature and avoided any interpretation. These summaries formed the basis of discussion between the three reviewers. At this second stage, the discussion was carried out in the context of the first research question- what are the experiences and beliefs of individuals' in relation to their condition, its associated morbidity and mortality risk and treatment?'. The discussion was exploratory in nature and no *priori* framework was imposed upon the findings at this stage. The aggregative findings of the studies, as consolidated in the code summaries, were deliberated, with examination of any similarities, differences and relationships between codes explored. From this discussion, 20 descriptive themes were identified. These descriptive themes were reflective of prevalent and persistent findings across the studies. Some of these themes were reflective of original codes used in the coding process, others were new themes created to capture more specific and detailed aspects of the original findings of coding process. For example, the findings captured using the code 'family influence' were further categorised into the descriptive themes 'parental influence upon treatment related behaviours' and 'FH and its treatment becomes normalised within families'.

One reviewer (FJK) then produced a draft summary of these descriptive themes which was reviewed and discussed with AS and EW before a final version was agreed upon.

Stage 3: Development of analytical themes

The generated descriptive themes captured and aggregated the beliefs and experiences of individuals with FH in relation to their condition and its treatment. The third stage of thematic synthesis aims to go beyond the primary content of the original papers to generate additional concepts or understandings.[40] This is considered an essential component of any qualitative synthesis methodological approach.[42] In this review, this meant using the descriptive themes to answer our research questions regarding how these beliefs and experiences may influence an individuals' adherence to treatment and to identify any enablers and/or barriers to this.

This was achieved by first examining each descriptive theme individually in the context of treatment adherence through consideration of the relationship between the content captured in each descriptive theme and individuals ability and/or inclination to adhere to treatment. Secondly, any relationships between the descriptive themes were explored to identify

For peer review only - http://bmjopen.bmj.com/site/about/guidelines.xhtml

common factors. Each reviewer (FJK, AS, EW) carried this out independently before meeting as a group to discuss further. From these discussions, over-arching analytical themes were identified. These analytical themes were then deliberated in the context of identifying enablers and barriers to treatment adherence which could be used to inform clinical practice, policy development and research intervention design. The reviewers met on three occasions to discuss their findings collaboratively. It was an iterative process in which the analytical themes were modified until the reviewers felt they adequately explained all the initial descriptive themes and identified enablers and barriers to treatment.

For example, three of the descriptive themes related to the involvement of other family members in an individuals' experiences of having FH and its treatment (FH and its treatment become normalised within families, parental influence upon treatment related behaviours and desire to protect children). From these descriptive themes, the reviewers identified the importance of the behaviours and beliefs of other family members upon an individuals ability and receptivity to adhering to treatment. This finding was captured in the analytical theme entitled 'family influence'. From this analytical theme, the reviewers identified two enablers to treatment adherence. These enablers were the delivery of care and treatment advice through family-based clinics and the commencement of treatment from a young age.

DRAFT

For peer review only - http://bmjopen.bmj.com/site/about/guidelines.xhtml

Appendix 1: Finalised coding frame

Code	Brief Description
Understanding/biological knowledge of FH	Account/description of what FH is, their understanding of its aetiology, its genetic transmission, its effect upon their body, any symptoms and any associated short and long term health implications
Perceived risk	The perceived risk of FH as a condition. Their thoughts/beliefs of short and long term health consequences of FH. Both genetic and behavioural associated risk.
Perceived seriousness	How serious/important FH and/or it's associated health consequences are believed to be
Family history	Account/description of family history of FH diagnosis, treatment and/or adverse outcomes such as death/serious illness
Life events	Significant milestones/occasions in life i.e. becoming parent, leaving school, getting married, ageing
Co-morbidities	Other illnesses/conditions that are not FH
Relative risk	Participant compares own risk to that of another person (family member, peer, abstract person) or to risk associated with another condition/illness
Management of condition	Account/description of the use/role of medicine or lifestyle in the treatment of FH.
Perceived efficacy of treatment	Perceptions/beliefs of the effectiveness of treating FH (medication, lifestyle and other)
Self-efficacy	The perception of an participant upon their own ability to follow treatment recommendations
Enablers and barriers for treatment	Any factors that help, enable, motivate OR Any factors that demotivate, stop or hinder a participant to seek and/or follow treatment advice
Ownership/personal responsibility	How a participant reflects/describes their perceived ownership of their condition and it's treatment. How much they perceive the condition to be their responsibility to manage/treat.
Emotional impact	Any emotion that FH diagnosis, management and/or associated health outcomes evokes in participants. Includes perceived stigma.
Impact on life	Any change participant has made to their life (everyday or longer term) as a result of their diagnosis of FH or its treatment
Professional support	Account/description of any involvement of healthcare professionals and/or medical procedures
Social support	Account/description of the role of family and/or friends in a patients' experience of their condition and it's management. practical or emotional support that individuals receive with regard to managing FH - i.e, treatment adherence.
Family influence	The influence of participants family upon their decision and ability to seek/adhere to treatment. Individuals' awareness of how others in their family network have dealt with screening and treatment and making decisions based on what other family members have done.
Information/help seeking	Accounts/descriptions of information or resources that participants would find useful
Parental views	Accounts/descriptions/thoughts/beliefs of parents in relation to their children.

For peer review only - http://bmjopen.bmj.com/site/about/guidelines.xhtml

BMJ Open

Enablers and barriers to treatment adherence in heterozygous familial hypercholesterolaemia: A qualitative evidence synthesis

Journal:	*BMJ Open*
Manuscript ID	bmjopen-2019-030290.R2
Article Type:	Research
Date Submitted by the Author:	19-Jun-2019
Complete List of Authors:	Kinnear, Fiona; University of Bristol, NIHR Bristol Biomedical Research Centre (Nutrition theme)<br>Wainwright, Elaine; Bath Spa University, Psychology department<br>Perry, Rachel; University of Bristol, NIHR Bristol Biomedical Research Centre (Nutrition Theme)<br>Lithander, Fiona; University of Bristol, NIHR Bristol Biomedical Research Centre (Nutrition Theme)<br>Bayly, Graham; University Hospitals Bristol NHS Foundation Trust, Department of Clinical Biochemistry<br>Huntley, Alyson; University of Bristol, Bristol Medical School<br>Cox, Jennifer; University of Bristol, NIHR Bristol Biomedical Research Centre (Nutrition Theme)<br>Shield, Julian; University of Bristol, NIHR Bristol Biomedical Research Centre (Nutrition Theme)<br>Searle, Aidan; University of Bristol, NIHR Bristol Biomedical Research Centre (Nutrition Theme)
**Primary Subject Heading**:	Qualitative research
Secondary Subject Heading:	Evidence based practice, Cardiovascular medicine, Health services research, Nutrition and metabolism, Patient-centred medicine
Keywords:	NUTRITION & DIETETICS, PREVENTIVE MEDICINE, QUALITATIVE RESEARCH, Coronary heart disease < CARDIOLOGY, Paediatric cardiology < CARDIOLOGY, Lipid disorders < DIABETES & ENDOCRINOLOGY

SCHOLARONE™
Manuscripts

Enablers and barriers to treatment adherence in heterozygous familial hypercholesterolaemia: A qualitative evidence synthesis

Fiona J Kinnear[1,2] Corresponding author, Elaine Wainwright[3], Rachel Perry[2], Fiona E Lithander[2], Graham Bayly[4], Alyson Huntley[5], Jennifer Cox[2], Julian P Hamilton-Shield[2], Aidan Searle[2].

1. Email Address: Fiona.kinnear@bristol.ac.uk; Telephone number: 0117 3421754
2. NIHR Bristol Biomedical Research Centre Nutrition Theme, University Hospitals Bristol NHS Foundation Trust and the University of Bristol, Bristol, UK
3. Psychology Department, Bath Spa University and Honorary Research Fellow, Department for Health, University of Bath, Bath, UK
4. Department of Clinical Biochemistry, University Hospitals Bristol NHS Foundation Trust, Bristol, UK

5. Population Health Science, Bristol Medical School, The University of Bristol, Bristol, UK

Word count excluding title page, abstract, references, figures and tables: 5700

For peer review only - http://bmjopen.bmj.com/site/about/guidelines.xhtml

ABSTRACT

Objectives Individuals with heterozygous familial hypercholesterolemia (FH) are at high risk of developing cardiovascular disease (CVD). This risk can be substantially reduced with lifelong pharmacological and lifestyle treatment however research suggests adherence is poor. We synthesised the qualitative research to identify enablers and barriers to treatment adherence

Design Thematic synthesis of qualitative studies

Data sources MEDLINE, Embase, PsycINFO via OVID, Cochrane library and CINAHL databases and grey literature sources were searched through September 2018

Eligibility criteria We included studies conducted in individuals with FH, and their family members, which reported primary qualitative data regarding their experiences of and beliefs about their condition and its treatment.

Data extraction and synthesis Quality assessment was undertaken using the Critical Appraisal Skills Programme for qualitative studies. A thematic synthesis was conducted to uncover descriptive and generate analytical themes. These findings were then used to identify enablers and barriers to treatment adherence for application in clinical practice.

Results 24 papers reporting the findings of 15 population samples (246 individuals with FH and 13 of their family members) across eight countries were included. Data captured within 20 descriptive themes were considered in relation to treatment adherence and six analytical themes were generated: risk assessment; perceived personal control of health; disease identity; family influence; informed decision making; and incorporating treatment into daily life. These findings were used to identify seven enablers (e.g. 'commencement of treatment from a young age') and six barriers (e.g. 'incorrect and/or inadequate knowledge of treatment advice') to treatment adherence. There was insufficient data to explore if the findings differed between adults and children.

Conclusions The findings reveal several enablers and barriers to treatment adherence in individuals with FH. These could be utilised in clinical practice to facilitate optimal adherence to lifelong treatment thereby minimising the risk of CVD in this vulnerable population.

PROSPERO registration number CRD42018085946

For peer review only - http://bmjopen.bmj.com/site/about/guidelines.xhtml

Strengths and limitations of this study

- This is the first thematic synthesis of the qualitative literature exploring the beliefs and experiences of individuals with familial hypercholesterolaemia to identify enablers and barriers to treatment adherence that can be targeted in clinical practice
- Robust procedures for conducting a thematic synthesis were adopted, informed by the Cochrane Qualitative Research Methods Group guidelines and they were reported in line with the Enhancing Transparency in Reporting the Synthesis of Qualitative Research statement
- The barriers and enablers were identified from themes which were representative of all the included studies, increasing their validity
- While included studies were conducted across eight countries, all were within the developed world which could limit the generalisability of the findings

For peer review only - http://bmjopen.bmj.com/site/about/guidelines.xhtml

INTRODUCTION

Heterozygous familial hypercholesterolaemia (FH) is one of the most common inherited genetic disorders, estimated to affect as many as 1 in 250 individuals worldwide.[1 2] Left untreated the exposure to chronically elevated levels of low density lipoprotein cholesterol (LDL-C) from birth confers an increased risk of cardiovascular disease (CVD),[2 3] with approximately 50% and 85% of affected women and men respectively experiencing a coronary event before the age of 65.[4] While this risk can be significantly reduced with early detection and treatment, many affected individuals remain at higher risk of premature CVD morbidity and mortality.[5-9] The most beneficial effects of treatment are evident in primary prevention before the onset of CVD.[5 10] With diagnostic rates are as low as 1% in some countries,[11] current efforts are focussed on identifying individuals with FH via screening and genetic testing programs.[12 13] Treated as outpatients and asked to follow lifelong treatment, it is critical to ensure that this increasing patient group are able to self-manage their disease. With many patients not reaching treatment targets[14-16] it is an area that warrants further investigation.

To improve adherence to treatment recommendations, an understanding of the factors affecting adherence is required. The American Heart Association (AHA) has recognised the need to gain a deeper understanding of the experiences of individuals with FH before addressing the further identified research gaps.[17] Preliminary research has found the beliefs and attitudes of FH patients towards the recommended treatment exert a significant effect upon their intention to engage in these behaviours.[18 19] Qualitative research can provide further insight to how these beliefs and attitudes are developed and the nature by which they may influence subsequent behaviours.[20] Its exploratory nature also allows for the identification of other factors influencing an individuals ability and motivation to comply with treatment.[21 22]

Qualitative research conducted in FH patients has found illness knowledge[23], risk perception[24], a lack of symptoms[25] and family history of disease[26] to influence treatment adherence. However, the transferability of these findings beyond the sample they are conducted in is limited.[27] Qualitative syntheses, which bring together the findings from individual qualitative studies, can be used to gain a more in depth understanding of the issue and identify common themes which are applicable to a wider range of contexts.[28 29] It is recognised as an important source of evidence to inform healthcare interventions and policy development[30-32] including those targeting treatment adherence[33-35] and is advocated by the World Health Organisation and the Cochrane Collaboration Group.[28 36] Given the limited literature concerning treatment adherence in FH, the results of this synthesis will also be compared to the results of research investigating treatment adherence in similar medical conditions.

For peer review only - http://bmjopen.bmj.com/site/about/guidelines.xhtml

Objectives

1. Identify how the experiences and beliefs of individuals with FH influence their adherence to pharmacological and lifestyle treatment recommendations

2. Explore if these findings differ between children and adults

3. Use the findings to generate new understanding of the enablers and barriers to treatment adherence to inform clinical practice

DRAFT For peer review only

For peer review only - http://bmjopen.bmj.com/site/about/guidelines.xhtml

MATERIALS AND METHODS

The methods used for this qualitative synthesis are briefly described below with full details available in the published protocol[37] and on the PROSPERO database (registration number CRD42018085946). Minor deviations to the protocol were made, outlined in supplementary file 1. The Enhancing Transparency of Reporting the synthesis of Qualitative research (ENTREQ) statement[38] has been followed and a checklist is available in supplementary file 2.

Search strategy

A comprehensive, systematic and pre-planned search was conducted to find all available qualitative evidence-full details are available in supplementary file 3.

Selection Criteria

Participants: Individuals with a clinical or genetic diagnosis of heterozygous familial hypercholesterolaemia (FH). No restrictions were placed on age or history or cardiovascular disease (CVD). Individuals with homozygous FH were not included.

Phenomena of interest: The experiences and beliefs of individuals with FH, and their family members, regarding their condition, its long-term health consequences and recommended pharmacological and lifestyle change treatment.

Types of studies: Only papers reporting primary qualitative data were included. Questionnaire studies were not included. Papers reporting both quantitative and qualitative data were included if the qualitative data could be independently extracted. Multiple papers reporting findings from the same sample of participants were included if they reported unique data.

Intervention/exposure: Treatment was defined as any behavioural action undertaken by an individual in an effort to manage their FH diagnosis.

Setting: No restrictions were placed on the country in which study was conduction, nor the location at which data were collected from individuals.

Quality appraisal

The methodological quality of the studies was assessed using the Critical Appraisal Skills Program (CASP) tool for reviewing qualitative research.[39] As the purpose of the quality appraisal was to determine the methodological strengths and limitations of studies included in the synthesis, the lead authors of each paper were contacted to obtain further information in an attempt to overcome the recognised issued of poor reporting in qualitative research. Full details of how this tool was used are available in supplementary file 4.

Data extraction

For peer review only - http://bmjopen.bmj.com/site/about/guidelines.xhtml

Methodological and contextual information from each paper were extracted into a table designed for this review by two reviewers independently (FJK, JC) after piloting in five papers. Two reviewers (FJK, AS) independently reviewed all text under the results, conclusions and discussion headings of all papers, as well as any supplementary files. Any data identified to be relevant to the research questions were extracted electronically using a tool designed for this review. In instances in which multiple papers reported the findings from a single study, data from the primary paper PhD theses were extracted first, before supplementary publications were reviewed for any additional, unique data. Results were compared and discussed until agreement was reached.

Data analysis

Thematic synthesis[40], a widely accepted and commonly used approach in qualitative syntheses, was used.[41 42] It involved three stages: line by line coding of the extracted data, generation of descriptive themes and development of analytical themes. Using NVivo software, two reviewers (FJK, AS) carried out the coding independently. The subsequent stages were carried out collaboratively between three reviewers (FJK, AS, EW). To enhance transparency, full details are available in supplementary file 5. The findings were discussed with three clinicians (JPHS, GB, PD) currently providing care to individuals with FH to help develop feasible and relevant recommendations for clinical practice.

Sensitivity analysis

To ensure the quality appraisal results were used in a meaningful way,[41 43] post-hoc sensitivity analysis was carried out by three reviewers (FJK,AS, EW) to examine the extent to which the synthesis results were affected by exclusion of poor quality papers, described in full elsewhere.[44] It involved examining if any themes were lost when each paper was removed from synthesis and evaluate if there was a significant impact upon the 'thickness' of findings reported within each theme. 'Thickness' refers to the depth, scope and context of findings which could influence the transferability and credibility of the results to the wider FH patient population.[45] This was carried out through discussion between three reviewers (FJK, AS, EW).

Patient and public involvement

Patients or members of the public were not involved in this study.

RESULTS

For peer review only - http://bmjopen.bmj.com/site/about/guidelines.xhtml

The titles and abstracts of 990 unique citations identified by the searches were screened, with 50 progressing to screening at the full-text level. Twenty-six papers were excluded at this stage due to: the full text not being available (n=1), no primary qualitative data being presented in the findings (n=6), the study population not having a clinical diagnosis of FH or inability to selectively extract data from those with a diagnosis in a mixed population (n=16) and data not being relevant to the aims of this review (n=3). Multiple papers reporting findings from the same sample of individuals and three PhD papers[46-48], two of which had supplementary papers published in addition to the originally reported theses, were included. Each paper was considered to be a separate primary paper and referenced separately. In total, 24 papers were included in the synthesis, comprising of 18 original[23 25 46-61] and six supplementary papers[24 26 62-65] reporting the findings of 15 population samples (Figure 1).

Characteristics of studies and participants

In total, 264 individuals with FH and 13 family members were involved, aged 8-69 years. Seven papers[24 25 46 58 59 62 63] reported findings from three samples which included individuals under 18 years. Four papers reported parental views of having children with FH.[25 56 58 59] Full characteristics of the included papers and samples are presented in Table 1.

For peer review only - http://bmjopen.bmj.com/site/about/guidelines.xhtml

Table 1: Characteristics of included studies

Sample number	Author & date of paper	CASP quality rating [a, b]	Research aim	Country	Recruitment setting	Sample Size [c]	Sample characteristics	Data collection methods
1	Agard et al, 2005[49]	Low	To explore the extent to which FH influences the life of the patients affected	Sweden	Outpatients treated at lipid clinic	23	10 M & 13 F; Mean age 48yrs (range: 31-67yrs); 4 with or had Hx of CVD	Face to face SSI
2	DeAngelis et al, 2017[50]	Low	To determine individual and group patient ideas and priorities regarding ways to enhance their own health	U.S.A.	Patients & family from patient centred outcomes research institute and outpatient clinic	7	6 FH patients, 1 family member	15 group meetings
3	Frich, 2007[46]	High*	To explore how individuals with FH perceive and manage their condition	Norway	Specialist clinic for metabolic lipid disorders	40	20 M & 20 F; Mean age 31yrs (range 14-57yrs); 7 had CVD symptoms; 19 had children	Face to face SSI
	Frich et al, 2006[62]	High*	To explore how patients with diagnosis of FH understand and perceive their vulnerability to CHD					
	Frich et al, 2007[63]	High*	To explore how patients at risk of CHD portray candidates for CHD					
	Frich et al, 2007[24]	High*	To explore patients' experiences of guilt and shame with regard to how they manage FH					
4	Hallowell et al, 2017[51]	High*	To investigate index patients' experiences of undergoing DNA testing as part of screening programme	Scotland	Two lipid clinics	38	17 M & 21 F; Mean age 52.6yrs (range 18-67yrs); 31 had children; 16 educated to university level	Face to face in depth interviews, (1 online)
	Jenkins et al, 2013[53]	Medium*	To explore patient's interpretations of their DNA results for FH					
		Low *	To explore the concept of inter-embodiment and its potential for					

For peer review only - http://bmjopen.bmj.com/site/about/guidelines.xhtml

	Jenkins et al, 2013[52]		advancing sociological research into illness biography and genetic identity					
5	Hardcastle et al, 2015[23]	High*	To investigate the perceptions and experiences of patients with a genetic diagnosis of FH involved in a cascade screening programme. To explore how these patients conceptualise FH and how such beliefs affect treatment compliance and lifestyle changes	Australia	Lipid disorders clinic	18	10 M & 8 F; Mean age 50.2 yrs (range 25-74 yrs); 2 had CVD symptoms	Face to face SSI
6	Hollands et al, 2012[54]	Low	Examine the impact of disease risk assessments based on both genetic and non-genetic information, or solely non-genetic information	U.K.	Lipid clinics at 11 hospitals	20	12 M & 8 F; Mean age 30.9yrs for DNA diagnosed & 40.7yrs for non-DNA; 17 white, 1 white Asian, 2 black Caribbean	3 telephone interviews
7	Hollman et al, 2004[55]	High*	To describe the QOL and to understand the underlying meaning of the concept of QOL in patients with FH	Sweden	Outpatient clinic	12	6 M & 6 F; 20-69yrs; 7 had children; 3 university level education; no Hx of CHV	Face to face SSI
8	Keenan et al, 2018[56]	Medium*	To explore parent's views and experiences of genetic testing and early treatment of children with FH in Scotland, experiences of their children's care pathway and to identify any barriers or facilitators in testing and treatment uptake	Scotland	Clinical genetic services and lipid clinics from 3 sites	17	6 M & 11 F; 20-69yrs; all white; 12 had post-secondary qualifications; 3 symptoms or Hx of CVD	SSI (15 face to face, 2 over phone)
9	Kirkegaard et al, 2014[57]	Medium*	Explore how cholesterol reducing medication and risk of CVD are interpreted by asymptomatic patients with high cholesterol	Denmark	5 GP centres.	3	1 M & 2 F; 24-62yrs; no CVD symptoms	Face to face SSI
10	Mackie et al, 2015[58]	High	Explore how family medical history, family narratives of medical experiences and AYA medical experiences together function as 'experiential evidence' and influence screening and treatment decisions	U.S.A	Paediatric preventative cardiology practice	24	12 AYAs with FH and 12 parents of AYAs with FH (4 dyads)<br><br>AYAs:6 M &, 6 F; Mean age 18.4yrs; 9 white, 1 black and 1 Asian<br>Parents: 2 M & 10 F; Mean age	Face to face SSI with AYA and parent separately
	Sliwinski et al, 2017[25]	High	To examine challenges transitioning to adult care for young adults with FH, and their parents, in the context of 2 developmental tasks: transitioning from childhood to early adulthood and summing					

For peer review only - http://bmjopen.bmj.com/site/about/guidelines.xhtml

			responsibility for self-management of a chronic disease				49.3yrs; 1 Asian, 9 white	
11	Meulenkamp et al, 2008[59]	High*	To study the experiences of children identified by family screening who were found to be a mutation carrier for a genetic CVD	Netherlands	Paediatric lipid clinic	16 children from 10 families	5 M & 11 F; 8-17yrs Number & age of parents not given	Face to face SSI (children and parents separately)
12	Mortensen et al, 2008[60]	Low	Comparative study to examine the QOL impact of FH in patients who had and had not reached the target of treatment	Denmark	Centre of inherited CVD	10	6 M & 4 F; 20-72yrs; no CVD Hx	Focus groups
13	Urke, 2016[47]	High	Explore how young adults, who stopped attending lipid clinic for medical and nutritional consultations, managed challenges related to living with FH and to the lifelong treatment	Norway	Outpatient clinic	11	6 M & 5 F; Median age 29yrs (range 26-35 yrs); 8 educated to university levels	SSI (9 face to face 2 over phone)
14	Weiner, 2006[48]	High *	How much and in which way patients with FH and professionals involved with the condition construct FH and CHD as genetic conditions	England	Lipid clinic	31	17 M & 14 F; Mean age 52 yrs (range 24-69 yrs); 31 white; 15 with current CVD	Face to face SSI
	Weiner and Durrington, 2008[26]	Medium*	To explore patients' understanding and experiences of FH and the significance of the hereditary aspect of the condition					
	Weiner, 2009[64]	Medium*	Consider how people with FH construct FH, high cholesterol and CHD					
	Weiner, 2011[65]	Medium*	Explore the notion of genetic responsibility, focussing particularly on responsibilities to family and kin					
15	Senior et al, 2002[61]	Low	Investigate perceptions of having an inherited predisposition to heart disease in people diagnosed with, and receiving treatment for FH	England	2 lipid clinics	7	5 M & 2 F; 39-58 yrs	Face to face SSI

SSI= semi structured interview; M= male; F= female; CHD = coronary heart disease; CVD = cardiovascular disease; QOL = Quality of life; AYA= adolescent and young adult; Hx = history

[a] CASP score: high=18-20; medium=14-17; low quality=<14.

[b] Papers for which lead author provided requested further information are marked with *

[c] The sample size and characteristics describe only those in sample with clinically diagnosed heterozygous FH and their family members.

Quality Appraisal and Sensitivity Analysis

Appraisal scores of papers ranged from 11-20 out of 20, with eleven rated high, seven medium and six low. (Table 1) The most common methodological limitations uncovered were relating to ethical issues, researcher reflexivity and rigour of data analysis. Consideration of a researchers' potential influence and bias upon data collection and analysis was critically examined fully in seven papers,[24 25 46 47 58 62 63] partially in 10[23 26 48 50 51 55 57 59 64 65] and not addressed in seven.[49 52-54 56 60 61] Ethical approval was obtained, or reasons given for exemption, in all but two papers,[60 61] however participants were not provided adequate information about withdrawal and anonymisation of data processes in a further 4 papers.[25 49 50 58] The data analysis was carried out by one researcher only in seven papers[23 26 47 48 56 64 65] and it was unclear if more than one person was involved at each stage of analysis in four papers.[51 52 60 61]

Eight lead authors responded to our request for further information, providing information for 16 of the 24 papers. Five of the six papers rated as low-quality were papers for which the author did not respond. This reflects our belief that low ratings may be reflective of poor reporting rather than poor methodology, supporting to our decision not to exclude papers. The sensitivity analysis carried out found that the removal of the five poor quality papers had no significant effect upon the synthesis findings- in both the descriptive and analytical themes uncovered and the depth of the findings. More detailed information of methodological and transferability issues is available in supplementary file 4.

Data analysis

Six analytical themes were derived from the findings captured by 20 identified descriptive themes, as displayed in Table 2 alongside illustrative quotes. Table 3 shows the occurrence of the descriptive themes within the extracted data from the 24 papers. While each analytical theme has a direct influence upon treatment adherence, they are not exclusive in nature and inter-theme relationships are evident as displayed in the thematic schema in Figure 2. Additionally, some themes by their integrative nature, had a greater influence upon treatment adherence as indicated by the shaded boxes. There were insufficient data regarding children and young people to explore whether the findings differed from adults.

Seven enablers and six barriers to treatment adherence (Table 4) were uncovered during the analysis of these themes and are described alongside the analytical themes below. In this section 'treatment' refers to both lifestyle and medication behaviours, unless otherwise specified.

For peer review only - http://bmjopen.bmj.com/site/about/guidelines.xhtml

Analytical themes

Risk assessment

Individuals lived experience of their disease, coupled with their beliefs concerning its known risks, increased or decreased their sense of vulnerability to its long-term health consequences. Knowledge of how FH had affected family members was the most prevalent factor considered by individuals when assessing their risk. Individuals with lived experience of a family member being ill or dying prematurely due to FH, had a heightened sense of risk.[46 48 49 52 55 56 58-62] Individuals unaware of FH in their families or with family members living a life unaffected by its consequences, perceived themselves at lower risk:[46 52 56 58 61 62] 'My dad's now in his 70s…it's not something I feel particularly threatened about having.'[56]

As FH does not 'make you feel ill',[52] individuals found having FH 'easy to forget, and easy not to take seriously.'[47] This was salient amongst younger individuals without existing CVD symptoms[23 25 47 48 58 59 65] for whom '…cholesterol always comes last. It will never be a focus until something happens to me.'[47] Older individuals who had lived through, or were currently experiencing CVD, perceived themselves at higher risk.[23 56 61 62] Others framed their perception of risk in the context of the risk they believed other diseases presented, concluding that FH health consequences were not as serious:[23 47 48 51 53 54 61] 'I didn't think it was life threatening, like being told you've got cancer.'[23]

For the majority of individuals, their risk assessment led to a perception that FH did not present a great risk to their current or long-term health.[23 47-49 51 56 59-61] This mismatch between the perceived and actual risk has been identified as a barrier to treatment adherence.

Perceived personal control of health

Individuals acknowledged the threat that FH posed to their health, but there was a widely held belief that they had the ability to modify their own personal risk.[24 47 49 51 53-62] They recognised that this required active engagement with treatment[23-25 47 49-51 53-56 58 61 62] and held themselves accountable for managing their disease[23-25 47-51 53-58 60-62] experiencing a 'bad conscience'[49] and 'guilt'[63] when they did not meet the expectations they had set themselves. Treatment was perceived to be effective[24 47 49 51 53-62] with individuals viewing FH as 'treatable'[48] and 'controllable'.[23] In particular, medication was regarded by individuals to be a mandatory and effective component of treatment.[24 47 49 51 53-62] They believed FH could be 'solved'[59] with medication and lead to achievement of cholesterol levels 'like most people'.[23] While individuals spoke of their efforts to change their lifestyle behaviours,[24 25 47 49 51 53-62]however many believed their cholesterol levels would not be 'radically changed'[61] by doing so[47 48 58 60] as 'doesn't matter what I eat or how much exercise I'm still going to have high cholesterol without tablets'.[23]

For peer review only - http://bmjopen.bmj.com/site/about/guidelines.xhtml

This confidence in the ability to successfully self-manage their condition was identified as an enabler to treatment adherence. The perceived effectiveness of medication led to a devaluing of the importance of following lifestyle treatment[23 47 48 57 58 60] and this prioritisation of medication was identified as a barrier to adhering to lifestyle treatment.

Disease identity

Individuals placed great importance, especially in social situations, to emphasis that they were ‘not to blame’[60] for their high cholesterol.[24 26 48 50 51 53 54 57 60 61 63] High cholesterol was associated with unhealthy lifestyles and individuals wished to distance themselves from this negative connotation.[24 48 54 57 60 61 63] A positive genetic test provided ‘a definitive’[51], rather than a possible, explanation for their high cholesterol.[50 53 54] and positively influenced individuals perceptions and behaviours.[24 50 51 53 54]If individuals had been following treatment of their volition before the diagnosis, it helped ‘reaffirm their commitment’ ‘reaffirm their commitment’[53] to treatment.[51 54]If they had been previously unaware of their condition it prompted them to seek treatment:[53 56] ‘I know now and can take preventative measures’.[54] Therefore, receiving a formal diagnosis was identified as an enabler to treatment adherence as being given a medical explanation empowered individuals to take control of their condition through engaging with treatment.

Family influence

Parents expressed a high level of concern about the well-being of their affected children[25 48 50 51 53 56 58 59] and this parental responsibility to care for children was identified as another enabler of treatment adherence. They assumed responsibility to ensure their children adhered to medical and lifestyle treatment,[25 48 50 51 53 56 58 59] taking action to ‘bring them up with healthy eating habits’[51] and ‘make sure that they take their medication’.[48] This involvement was reflected in the finding of individuals attributing their current treatment knowledge and behaviours to their parents:[47-49]: ‘everything I’ve learned from home’.[47] Parents also made treatment-related decisions on their behalf [25 48 50 53 58 59]until they were ‘old enough to decide.’[56] As such, the early adulthood years presented a challenge for treatment adherence as the young adults transitioned from being under the care of their parents to assuming responsibility for their behaviours.[25 47]

Growing up surrounded by family members following treatment recommendations and establishing healthy behaviours from a young age was found to instil lifelong habits in individuals.[25 47 48 56 58 59] Those who had grown up from a young age alongside diagnosed family members spoke of their condition and its treatment as something that had become ‘normalised’[47] as it was all they had ever known.[25 48 56 58 59] Those who had parents who had bad experiences of medication were apprehensive about taking tablets,[58] but for many it led to the view that taking medication was ordinary[56] and not a ‘big deal’.[58]

Two enablers to treatment adherence were identified from these findings: commencement of treatment from a young age and having other family members following similar treatment regimes.

For peer review only - http://bmjopen.bmj.com/site/about/guidelines.xhtml

Informed decision making

Individuals lacked an in-depth understanding of their disease and its treatment,[23-25 47-51 56-59 61] with many having 'unanswered questions'[49] and requesting more information.[25 49-51] Misconceptions and false information regarding the role of treatment for FH were prevalent:[24 25 47-49 51 56-59 61]: 'you can actually eat a lot of fat and the medicine takes care of it.'[23] Individuals were worried about the longer-term impact of statin therapy on their, and their children's, health[49 58] as 'it is a recent drug, and you don't know what the long term effect could be.'[56] Lived experience of side effects were reported by some individuals[49 58 60] and many more were fearful of developing them in the future[55 56 58] as 'many others have severe side effects from what I'm taking'.[60] This incorrect and/or inadequate knowledge of treatment advice and concerns over the short- and long-term use of lipid lowering medication were identified as barriers to treatment adherence.

Individuals frequently mentioned their encounters with healthcare professionals HCPs,[23 24 46-48 50 52 53 56 57 59 60] viewing them as playing a 'big role'[25] in their 'team approach'[58] to the management of their FH. Regardless of whether individuals recalled these encounters in a positive[24 25 47 48 50 56 58]or negative[24 46 47 56 60] light, these interactions and relationships with HCPs influenced their understanding of FH and its treatment.

Integrating treatment into daily life

Individuals did not feel they had to make many changes to their everyday life as a result of their diagnosis.[23 47-49 51 54 61]Their disease did not them from 'living the life they wanted'[47] or require consideration when making life decisions[23 47 49 54 61] such as having children.[48 51]However, when faced with other commitments, such as family and career obligations, individuals found it more difficult.[23 25 47 49 54 60 62] During these periods individuals tended to be less focused on managing their disease viewing it as something they could pick up again when they had more time and energy.[23 25 47 56 62]This prioritisation of other life events over the self-management of condition was identified as a barrier to treatment adherence.

The treatment recommendations were perceived to be simple to follow and to have little impact on their QOL.[23 47-49 51 53-56 61] However, this perception is in stark contrast to the actual lived experiences of following treatment- especially the lifestyle recommendations. Dietary advice was perceived to be restrictive and interpreted by individuals to mean they could not eat their favourite foods[24 25 47 48 57 59] or enjoy social occasions:[24 25 54 57 59 60] 'I won't bother eating food I don't like, just to follow a certain diet'.[47] Additionally, individuals were concerned about the opinions of their peers in social situations in which they felt they had to make certain dietary choices.[25 47 48 59 60] These findings were prominent amongst younger individuals.[25 47 59]As a result, the dietary advice was the 'most difficult aspect'[49] of treatment, with many reporting they struggled to follow them at all times.[23-25 47 48 57 59 60] This finding of dietary advice being perceived as difficult to follow was identified as a barrier to adherence.

For peer review only - http://bmjopen.bmj.com/site/about/guidelines.xhtml

Reflective of the difficulties faced when trying to follow treatment guidelines, individuals expressed a need for additional information [23 49 50 56] and 'guidelines in order to help you start that change'.[25] Some sought additional information from their HCPs[23 25 49 50 56], while others called for practical advice and educational resources[25 49 50 56], as 'everyone knows the theory, but putting it to practice is quite hard'.[23] From this, practical resources and support for following lifestyle treatment advice was identified as an enabler to treatment adherence.

DRAFT
For peer review only

Table 2: Analytical themes and their composite descriptive themes with illustrative quotes

Analytical theme	Descriptive themes	Illustrative quotes from participants (1st order)	Illustrative interpretations from authors (2nd order)
Risk assessment	FH is a silent disease	'not a condition that has any symptoms, that makes you feel ill or anything.'[52]	'The majority of interviewees did not look upon the condition as a disease…If they were not affected by a cardiac disease…they regarded themselves as healthy.'[49]
	Family history modifies perception of FH related threat to health	'I'm not going to get past sixty. Dad never got past sixty.'[53]	'To them, reaching the age of death of a parent with FH was anticipated with fear of having a heart attack themselves.'[60]
	FH is not as threatening to health as other conditions	'Its not that bad….Its not like having something like Huntington's or something like that.'[51]	They mentioned conditions with more drastic consequences such as allergies, epilepsy or diabetes.'[47]
Perceived personal control of health	FH is a manageable condition	'well it's treatable isn't it by diet and drugs. It's not something that's incurable.'[48]	'FH carrier children demonstrated high feelings of control over their condition.'[59]
	Individuals feel personally responsible for managing their FH	'it means you could be in danger of like what could possibly happen like in the future if you don't change anything.'[58]	'FH patients have a strong desire to empower themselves in order to improve their own health.'[50]
	FH medication is effective	'I believe that as I am taking the pills that my risk of heart attack is no greater than anyone else of my age or weight.'[61]	'Preventative medical treatment built confidence in the potential for living a long life.'[55]
	FH lifestyle treatment viewed as less important than medication	'I could never get that down no matter 'ow much dieting or exercise I do…so it can only be reduced through medication.'[48]	'Many tended to devalue the importance of lifestyle changes in controlling FH and place their hope in medication.'[23]
Disease identity	Importance of establishing that high cholesterol levels are not self-inflicted	'It enables me to emphasise that it is not my fault, that it's something inherited.'[62]	'they always described FH as a hereditary condition to underline that their cholesterol issues were not due to unhealthy lifestyle.'[60]
	Receiving genetic diagnosis provides certainty	'I guess it is a relief in a funny way because I had an answer to what was quite a surprising medical condition that I had…so at least I know now and can take preventative measures.'[54]	it provided an aetiological explanation and diagnostic label, confirmed current risk management practices…'[24]
The influence of family	Desire to protect children	'we want to help him…[so] we have decided to give him statins until he is 16…we've covered him until he's old enough to decide for himself.'[56]	'In fact, the main concern for the affected parents appeared to be the well-being of their children…'[49]
	Parental influence upon treatment related behaviours	'my parents, specifically my mom, were really integral in teaching us types of food to eat..'[25]	'AYAs expressed how their perceptions of their parents experience have influenced their perceptions of the respective treatment options.'[58]
	FH and its treatment become normalised within families	'Since I grew up with FH and had a relatively good diet and good habits and routines, it makes it easier.'[47]	'FH carrier children typically reported it had become habit to maintain a healthy, non-fat diet. Commonly the whole family, including the non-carriers, kept to the same diet restrictions.'[59]
Informed decision making	HCP interactions	'My daughter. I don't think she really understood what [high cholesterol] really meant until she came here and talked with doctor.'[58]	'The doctors presentation of FH, however, influenced all patients perceptions of the risk and severity of the diagnosis.'[60]
	Inadequate and/or incorrect knowledge about FH & treatment	'in the newspapers, the stories that you cut out butter, red meat, etc., and you'll be okay.'[61]	'Many informants still had unanswered questions or were felt to lack relevant knowledge.'[49]
	Concerns about side effects of FH medication	'would I be able to have children at all after taking all these medicines for years?'[49]	'Parents reported having strong concerns about statin treatment in children, not only because of their long-term safety but also potential side effects.'[56]
Inco rpor	FH and it's treatment does not have big impact upon life	'You don't have to plan your life around it. You don't have to wonder, can you have children or not.'[51]	'FH was not viewed as a significant burden, but more of a lifestyle adjustment, involving a healthy diet, exercise, and statin treatment from an early age.'[56]

For peer review only - http://bmjopen.bmj.com/site/about/guidelines.xhtml

ating treatmen t into daily life	Balancing FH treatment with other competing priorities	'Our two children, who were often ill….My husband…travelled all the time, so I almost had more than I could put up with at that moment.'[62]	'Young adults also articulated challenges maintaining diet and exercise regimes while adjusting to a new routine and environment at college or in workforce.'[25]
	Lifestyle advice treatment is restrictive and difficult to follow	'I've changed my diet as much as I can… don't want to bother too much and speculate, live an unworthy life and diet at the age of seventy. I'd rather be happy and die when I'm fifty.'[24]	'Making dietary changes had been the worst aspect of their condition, and this included people who already had CHD.'[62]
	Social implications of following FH treatment	'Some people comment on the things I eat. And then I'm like 'well actually I have to eat this because I've got FH and I have to watch my diet.'[54]	'10 young adults articulated how concern over peers' opinions or overt peer pressure-restricted social activities centered around eating.'[25]
	Desire for further support and guidance	I think having the resources [would make it easy to adhere to lifestyle treatment]…like seeing a nutritionist that can give you options….'[25]	'..expressed a desire to be able to access educational resources in one place and for a way to reach out to others who could provide solidarity, comfort and aid with management of FH.'[50]

AYA= adolescent and young adult; HCP = healthcare professional; CHD = cardiovascular heart disease

For peer review only - http://bmjopen.bmj.com/site/about/guidelines.xhtml

Table 3: Occurrence of descriptive themes across the included papers and samples [a]

		Descriptive themes																			
Sample number	Paper	FH is a silent disease	Family history modifies perception of FH related threat to health	FH is not as threatening to health as other conditions	FH is a manageable condition	Individuals feel personally responsible for managing their FH	FH medication is effective	FH lifestyle treatment viewed as less important that medication	Importance of establishing that high cholesterol levels are not self-inflicted	Receiving genetic diagnosis provides certainty	Desire to protect children	Parental influence upon treatment related behaviours	FH and its treatment become normalised within families	HCP relationships	Inadequate and/or incorrect knowledge about FH and its treatment	Concerns about side effects of FH medication	FH and its treatment does not have big impact upon life	Balancing FH treatment with other competing priorities	Lifestyle advice treatment is restrictive and difficult to follow	Social implications of following FH treatment	Desire for further support and guidance
1	Agard et al, 2005[49]	✓	✓		✓	✓	✓				✓	✓		✓	✓	✓	✓	✓	✓	✓	✓
2	DeAngelis et al, 2017[50]					✓				✓	✓			✓	✓						✓
3	Frich, 2007[46]		✓											✓	✓						
	Frich et al, 2006[62]		✓		✓	✓	✓											✓			
	Frich et al, 2007[63]								✓						✓						
	Frich et al, 2007[24]				✓	✓			✓	✓				✓	✓				✓	✓	
4	Hallowell et al, 2017[51]			✓	✓	✓	✓		✓	✓	✓	✓			✓		✓				✓
	Jenkins et al, 2013[53]			✓	✓	✓	✓		✓	✓	✓			✓			✓				
	Jenkins et al, 2013[52]	✓	✓											✓							
5	Hardcastle et al, 2015[23]	✓	✓	✓	✓	✓	✓	✓			✓			✓	✓		✓	✓	✓		✓
6	Hollands et al, 2012[54]					✓			✓	✓							✓			✓	
7	Hollman et al, 2004[55]		✓		✓	✓	✓									✓					
8	Keenan et al, 2018[56]		✓		✓	✓	✓				✓	✓		✓	✓	✓		✓	✓		✓
9	Kirkegaard et al, 2014[57]		✓			✓	✓	✓	✓					✓	✓				✓	✓	
10	Mackie et al, 2015[58]	✓	✓		✓	✓	✓	✓			✓	✓	✓	✓	✓	✓					
	Sliwinski et al, 2017[25]	✓				✓					✓	✓	✓	✓	✓			✓	✓	✓	✓
11	Meulenkamp et al, 2008[59]	✓	✓		✓	✓	✓				✓	✓	✓	✓	✓		✓		✓	✓	
12	Mortensen et al, 2008[60]				✓	✓	✓	✓	✓		✓			✓		✓		✓		✓	
13	Urke, 2016[47]	✓		✓	✓	✓	✓	✓				✓	✓	✓	✓		✓	✓	✓	✓	
14	Weiner, 2006[48]	✓	✓	✓	✓	✓	✓	✓	✓		✓	✓	✓	✓	✓		✓	✓	✓		
	Weiner and Durrington, 2008[26]								✓												
	Weiner, 2009[64]								✓												
	Weiner, 2011[65]	✓																			

15	Senior et al, 2002[61]		✓	✓	✓	✓	✓		✓						✓		✓				

HCP = healthcare professional

[a] Themes identified within supplementary papers were only documented if they were evident in extracted data not reported in the primary paper and vice versa.

DRAFT

For peer review only

Table 4: Identified enablers and barriers to treatment adherence

Enablers	Barriers
Other family members following treatment regime	Mismatch between perceived and actual risk
Commencement of treatment from a young age	Concerns over the use of lipid lowering medication
Parental responsibility to care for children	Prioritisation of medication over lifestyle treatment
Confidence in ability to successfully self-manage their condition	Lifestyle treatment is difficult to comply with
Receiving formal diagnosis of FH	Prioritisation of other life events
Practical resources & support for following lifestyle treatment	Inadequate and/or incorrect knowledge of treatment advice
A positive relationship with healthcare professional	

For peer review only - http://bmjopen.bmj.com/site/about/guidelines.xhtml

Discussion

This synthesis has produced new insights into the factors influencing treatment adherence in FH which have implications for clinical practice and future research.

We found that individuals did not perceive FH as a threat to their health except in those who had experienced symptoms of CVD or had a family history of FH related CVD, as previously reported by others.[66-69] This low perception of risk may be the result of the disease being relatively symptomless and the adverse consequences too far in the future to comprehend, This idea is reinforced by studies reporting heightened perceived risk amongst older individuals[70] and young adults perceiving their health to be average or above that of the general population.[16] The minimal threat to health may explain the findings that being diagnosed with FH does not increase psychosocial dysfunction in children[71 72], nor negatively impact upon self-reported quality of life (QOL) or rates of depression and anxiety in adults.[73-76] While these findings are positive, individuals who do not view their disease as a serious threat may be less motivated to adhere to treatment which may explain the findings of higher self-reported medication adherence in older individuals[77 78] and high non-adherence rates in individuals under 36 years.[79] These findings are concerning as individuals who do not adhere fully to treatment have been found to have higher levels of LDL-C.[77 79 80] Furthermore, while treatment has substantially reduced the risk of CVD individuals still remain at a higher risk than the general population.[9 81 82] This may be a consequence of LDL-C targets not being met by large numbers of treated adults[15 16 79 80 83] and children[84 85] and/or the presence of other risk factors independently associated with CVD.[86 87]

Our findings suggest this low risk perception may be mediated by beliefs that the risks are avoidable through effective treatment, in line with previous research.[16 66 72 88] These beliefs have been found to positively influence attitudes towards medication, increasing self-reported intentions to comply with medication [19] and rates of adherence.[89] However, individuals attitudes toward treatment behaviours may have a greater influence upon their intention to engage in treatment than their beliefs.[18] Our findings of negative attitudes toward certain aspects of treatment are therefore important to explore. We found individuals to perceive dietary recommendations as restrictive and impacting upon their QOL, as have others.[72 90] Some also believed they were unnecessary if taking medication, likely explaining low uptakes of lifestyle treatment compared to medication.[66 91] We also found negative attitudes towards medication due to side effects and anxieties about long-term safety, similar to others.[16 83 92] In contrast to these studies, we found anxiety about the development of side effects and complications of long-term use to be more prevalent than lived experience of side-effects. These negative attitudes are surprising as the dietary recommendations do not differ substantially from those for the general population and the safety and tolerability of statins have been demonstrated in adults[93] and children.[94-96]

For peer review only - http://bmjopen.bmj.com/site/about/guidelines.xhtml

Our finding of widespread inadequate knowledge of the treatment recommendations may explain the negative attitudes. It has been reported previously that awareness of the role of PA in treatment is low[97] and while individuals are mindful of the need for dietary treatment little is known about the depth of this knowledge.[72 90 97] This finding may be the result of the inconsistency in treatment advice provided with many not receiving the recommended lifestyle advice[91 98 99] or medication treatment[83 85 91 98 100 101] and for those that do, it is often not provided by HCPs with specialist FH knowledge.[91 99] As a result, we found many individuals are left wanting more information about treatment, in line with previous research,[91 97] This is concerning as many report using the internet to search for such information[91] which cannot be easily regulated and may be fuelling our further finding of a high prevalence of incorrect knowledge. Furthermore, individuals may be falsely interpreting negative media coverage of statin medication[102] to be relevant to their condition. This may be negatively influencing adherence to treatment as concerns about general medication overuse have been found to be heavily influential in shaping attitudes toward FH medication[19] Ensuring individuals have a comprehensive and factually correct understanding of the treatment recommendations is therefore essential to optimise adherence.

As this synthesis highlighted that parents take responsibility for their childs' treatment, it is important to ensure they are knowledgeable about the recommendations to help their children develop healthy habits from a young age. Previous research has found that children who follow dietary guidelines from a young age have more positive attitudes towards this aspect of treatment[71] and have improved dietary intakes in childhood[103-105] which are maintained into young adulthood.[106] Furthermore, forgetfulness is frequently reported as a reason for medication non-adherence[16 72 77 78 80 92] and starting treatment at a young age may help overcome this by instilling a routine, as found by others.[107] It is also important to ensure that when individuals reach an age where they become responsible for their own care, they themselves are equipped with the relevant knowledge to continue to make informed decisions. While there was insufficient data to draw conclusions about best practice for this age group, it appears that transitioning from living at home, adjusting to new routines and prioritising other things in life are common barriers to be targeted.[25 47]

Our findings also highlight the importance of receiving a genetic confirmation of FH. Receiving a medical diagnosis empowered individuals to take control of their condition, providing motivation to continue or commence medication and lifestyle treatments. The positive influence of diagnosis upon medication efficacy beliefs and adherence have been reported in previous research.[67 68 108 109] However, in contrast to our findings it has been reported that positive genetic results have either no effect[68] or weaken beliefs[108] regarding the efficacy of lifestyle treatment. However, in both cases the changes in beliefs did not have a negative impact upon their actual behaviours. Given our further finding that individuals find medical diagnosis useful in social situations, a common identified barrier to adhering to dietary recommendations, it

For peer review only - http://bmjopen.bmj.com/site/about/guidelines.xhtml

may be that genetic diagnosis exerts positive effect upon adherence beyond its influence of illness and treatment beliefs.

Strengths and limitations

Our thematic synthesis adhered to ENTREQ guidelines and used transparent and robust methodology. The comprehensive search strategy, involvement of more than one researcher at each stage of analysis, input from clinicians to corroborate the interpretation of the results and detailed appraisal of the included studies strengthen our findings. The analytical themes generated were produced from descriptive themes that were each evident across a large number of the included papers. The synthesis included data from 264 individuals with FH and 13 family members across eight countries, encompassing a wide range of ages, duration of diagnoses, primary and secondary CVD prevention and regional differences in healthcare provision. However, all individuals were from developed countries, the majority had high education levels and there were few from ethnic minority groups. This may limit the generalisability of the findings to all individuals with FH. Furthermore as the majority were recruited from lipid clinics and their beliefs may not reflect those opting out of treatment for their condition. Lastly, there were insufficient papers to explore if the factors influencing treatment adherence differ between adults and children with FH and care should be taken when extrapolating results to younger individuals.

Implications for clinical practice

We have identified seven enablers and six barriers to treatment adherence (Table 4) to be considered by any HCP delivering advice to individuals with FH and have produced the following 12 suggestions for clinical practice:

1. Ensure individuals are aware of the risk to their health, without instilling fear through emphasising the effectiveness of medical and lifestyle treatment

2. Where possible, ensure all individuals receive genetic confirmation of their condition

3. Communicate that despite the asymptomatic nature of the condition, adhering to treatment from a young age will deliver the greatest benefits to health

4. Discuss medication within an FH context, emphasising its necessity and distinguishing it from the use of medication in treatment of other causes of high cholesterol

5. Provide reassurance that medication is safe and side effects uncommon, with reference to relevant clinical guidelines indicating their safety for use by children highlighted to parents

6. Inform patients that side effects are specific to each type of medication and encourage discussion of any problems so alterative medications can be offered

For peer review only - http://bmjopen.bmj.com/site/about/guidelines.xhtml

7. Communicate dietary advice as being a lifestyle change rather than a restrictive diet with advice tailored to the individual needs and preferences of each individual

8. Ensure individuals have a factually correct understanding of the dietary recommendations and provide credible resources individuals can access if they require further support or guidance

9. The benefits of adhering to lifestyle treatment for management of their disease and their overall well-being, should be revisited at each clinic appointment

10. Treatment should begin early, with parents advised that prior to medication, dietary recommendations can be followed from the age of five. Non-affected family members can also be encouraged to follow guidelines, facilitating a family-based approach to aid adherence.

11. Treatment advice to be provided in family-based clinics if possible, or ensure adult and paediatric services are closely linked

12. Adolescent patients to be offered opportunity to transition to an adult clinic between the ages of 16-18 to take responsibility for their own treatment before they leave home

Comparison with treatment adherence in similar medical conditions

The limited literature regarding treatment adherence in FH makes comparison of findings with the present synthesis difficult. However, extensive research has been conducted into treatment adherence for other chronic conditions which are also asymptomatic in the early stages such as hypertension, high cholesterol from non-genetic conditions and type 2 diabetes mellitus, for which treatment adherence rates are also low.[110 111] While it is beyond the scope of this review to compare and contrast the findings in detail, overall the enablers and barriers were similar to those found to exist for individuals following treatment for these similar conditions. For example, negative perceptions of medication, beliefs that treatment is not necessary due to lack of symptoms, medication side effects and a lack of knowledge about treatment and/or disease were identified as barriers to adherence for those advised treatment to manage risk factors for the primary and secondary prevention of CVD.[112-114] Furthermore, similar findings have been reported in individuals with type 2 diabetes mellitus.[115-117] A unique finding of the present synthesis, however, was that starting treatment from a young age and being surrounded by other family members following treatment facilitates adherence. This is reflective of the genetic inheritance pattern in which an individual will always have one affected parent, which is uncommon in other chronic conditions. Although support from family members, and the involvement of parents, has been identified as an enabler to treatment adherence for individuals with type 2 diabetes mellitus,[115 118 119] the adherence behaviours that parents with FH model to family members is of particular importance in the treatment of FH.

For peer review only - http://bmjopen.bmj.com/site/about/guidelines.xhtml

Future research

With treatment most effective when started at a young age,[6 10 85] and our findings of a positive effect upon later life adherence, further qualitative research exploring the perspectives of children is required to allow HCPs to tailor advice to support maximal adherence during this crucial period. The findings of widespread inadequate and/or incorrect knowledge of the treatment recommendations warrants investigation into what advice is being given, and by whom. As individuals who have self-selected to receive treatment have concerns about medication, it is likely that there are many individuals opting not to receive treatment for themselves or their child due to these concerns. Future research is needed to explore their perceptions to develop effective interventions that could encourage them to seek treatment.

Conclusions

This qualitative evidence synthesis has systematically reviewed and synthesised the available evidence concerning the experiences and beliefs of individuals with FH regarding their condition and its treatment. It has uncovered several enablers and barriers that are to be utilised in clinical practice to facilitate optimal treatment adherence in this high-risk clinical population group. It has also highlighted significant research gaps which need to be addressed to gain a more comprehensive understanding of how these individuals can be supported to adhere to lifelong treatment.

Acknowledgements

We would like to thank Catherine Borwick (Research Engagement Librarian) and Dr. Alison Gregory (Research Fellow) at The University of Bristol for their expertise and assistance in the development of this evidence synthesis. We would also like to thank Dr. Paul Downie (Consultant at University Hospitals Bristol and Royal United Hospitals Bath NHS Foundation Trusts) for his input into translating the findings into clinical recommendations.

Author contributions: FJK and RP devised and carried out the search strategy. FJK, RP, FEL and JPHS carried out the study screening and selection stage. FJK and JC carried out the study characteristic extraction stage. FJK and AS carried out the results data extraction, quality appraisal, data analysis and interpretation. EW also carried out the data analysis and interpretation stages. AH contributed to the development and presentation of the qualitative methodology and results. FJK, JPHS and GB translated findings into clinical implications. FJK prepared the manuscript. All authors reviewed the manuscript and approved the final version.

For peer review only

Funding: This evidence synthesis is funded by the National Institute for Health Research NIHR Bristol Biomedical Research Centre (Nutrition theme) at University Hospitals Bristol NHS Foundation Trust and The University of Bristol.

Disclaimer: The views expressed in this publication are those of the authors and not necessarily those of the NHS, the NIHR or the Department of Health and Social Care.

Competing interests: None declared.

Data availability statement

Data are available upon reasonable request. Data extracted from each of the 24 publications have been stored in word document files and are available by emailing the first author.

Abbreviations:

FH: Familial Hypercholesteroleamia

LDL-C: Low density lipoprotein cholesterol

CVD: Cardiovascular disease

CASP: Critical apprisal skills programme

PA: Physical activity

AHA: The American Heart Association

ENTREQ: Enhancing transparency in reporting the synthesis of qualitative research

CHD: Coronary heart disease

SSI: Semi-structured interview

AYA: Adult and young adolescent

HCPs: Healthcare professionals

QOL: Quality of life

Figure Legends

Figure 1: PRISMA Flow diagram

Figure 2: Thematic schema illustrating influence of analytical themes upon treatment adherence

For peer review only - http://bmjopen.bmj.com/site/about/guidelines.xhtml

References

1. Akioyamen LE, Genest J, Shan SD, et al. Estimating the prevalence of heterozygous familial hypercholesterolaemia: a systematic review and meta-analysis. *BMJ Open* 2017;7(9)
2. Benn M, Watts GF, Tybjærg-Hansen A, et al. Mutations causative of familial hypercholesterolaemia: screening of 98 098 individuals from the Copenhagen General Population Study estimated a prevalence of 1 in 217. *European heart journal* 2016;37(17):1384-94. doi: 10.1093/eurheartj/ehw028
3. Wong B, Kruse G, Kutikova L, et al. Cardiovascular Disease Risk Associated With Familial Hypercholesterolemia: A Systematic Review of the Literature. *Clinical therapeutics* 2016;38(7):1696-709. doi: 10.1016/j.clinthera.2016.05.006 [published Online First: 2016/06/05]
4. Civeira F. Guidelines for the diagnosis and management of heterozygous familial hypercholesterolemia. *Atherosclerosis* 2004;173(1):55-68. doi: https://doi.org/10.1016/j.atherosclerosis.2003.11.010
5. Humphries SE, Cooper JA, Seed M, et al. Coronary heart disease mortality in treated familial hypercholesterolaemia: Update of the UK Simon Broome FH register. *Atherosclerosis* 2018;274:41-46. doi: https://doi.org/10.1016/j.atherosclerosis.2018.04.040
6. Mohrschladt MF, Westendorp RG, Gevers Leuven JA, et al. Cardiovascular disease and mortality in statin-treated patients with familial hypercholesterolemia. *Atherosclerosis* 2004;172(2):329-35. doi: 10.1016/j.atherosclerosis.2003.11.007 [published Online First: 2004/03/17]
7. Versmissen J, Oosterveer DM, Yazdanpanah M, et al. Efficacy of statins in familial hypercholesterolaemia: a long term cohort study. *BMJ* 2008;337
8. Besseling J, Hovingh GK, Huijgen R, et al. Statins in Familial Hypercholesterolemia: Consequences for Coronary Artery Disease and All-Cause Mortality. *Journal of the American College of Cardiology* 2016;68(3):252-60. doi: 10.1016/j.jacc.2016.04.054 [published Online First: 2016/07/16]
9. Mundal LJ, Igland J, Veierod MB, et al. Impact of age on excess risk of coronary heart disease in patients with familial hypercholesterolaemia. *Heart (British Cardiac Society)* 2018 doi: 10.1136/heartjnl-2017-312706 [published Online First: 2018/04/07]
10. Neil A, Cooper J, Betteridge J, et al. Reductions in all-cause, cancer, and coronary mortality in statin-treated patients with heterozygous familial hypercholesterolaemia: a prospective registry study. *European heart journal* 2008;29(21):2625-33. doi: 10.1093/eurheartj/ehn422 [published Online First: 2008/10/09]
11. Nordestgaard BG, Chapman MJ, Humphries SE, et al. Familial hypercholesterolaemia is underdiagnosed and undertreated in the general population: guidance for clinicians to prevent coronary heart disease : Consensus Statement of the European Atherosclerosis Society. *European heart journal* 2013;34(45):3478-90. doi: 10.1093/eurheartj/eht273
12. Kerr M, Pears R, Miedzybrodzka Z, et al. Cost effectiveness of cascade testing for familial hypercholesterolaemia, based on data from familial hypercholesterolaemia services in the UK. *European heart journal* 2017;38(23):1832-39. doi: 10.1093/eurheartj/ehx111 [published Online First: 2017/04/08]
13. Louter L, Defesche J, Roeters van Lennep J. Cascade screening for familial hypercholesterolemia: Practical consequences. *Atherosclerosis Supplements* 2017;30:77-85. doi: 10.1016/j.atherosclerosissup.2017.05.019 [published Online First: 2017/11/04]
14. Hartgers ML, Besseling J, Stroes ES, et al. Achieved LDL cholesterol levels in patients with heterozygous familial hypercholesterolemia: A model that explores the efficacy of conventional and novel lipid-lowering therapy. *J Clin Lipidol* 2018 doi: 10.1016/j.jacl.2018.04.002 [published Online First: 2018/06/24]
15. Pijlman AH, Huijgen R, Verhagen SN, et al. Evaluation of cholesterol lowering treatment of patients with familial hypercholesterolemia: a large cross-sectional study in The Netherlands. *Atherosclerosis* 2010;209(1):189-94. doi: https://doi.org/10.1016/j.atherosclerosis.2009.09.014
16. Langslet G, Bogsrud MP, Halvorsen I, et al. Long-term follow-up of young adults with familial hypercholesterolemia after participation in clinical trials during childhood. *Journal of Clinical Lipidology* 2015;9(6):778-85. doi: https://doi.org/10.1016/j.jacl.2015.08.008
17. Gidding SS, Champagne MA, de Ferranti SD, et al. The Agenda for Familial Hypercholesterolemia: A Scientific Statement From the American Heart Association. *Circulation* 2015;132(22):2167-92. doi: 10.1161/cir.0000000000000297 [published Online First: 2015/10/30]
18. Hagger MS, Hardcastle SJ, Hingley C, et al. Predicting Self-Management Behaviors in Familial Hypercholesterolemia Using an Integrated Theoretical Model: the Impact of Beliefs About Illnesses and

For peer review only - http://bmjopen.bmj.com/site/about/guidelines.xhtml

Beliefs About Behaviors. *International Journal of Behavioral Medicine* 2016;23(3):282-94. doi: 10.1007/s12529-015-9531-x

19. Hagger MS, Hardcastle SJ, Hu M, et al. Effects of medication, treatment, and behavioral beliefs on intentions to take medication in patients with familial hypercholesterolemia. *Atherosclerosis* 2018;277:493-501. doi: https://doi.org/10.1016/j.atherosclerosis.2018.06.010
20. Green J, Britten N. Qualitative research and evidence based medicine. *BMJ : British Medical Journal* 1998;316(7139):1230-32.
21. George M, Freedman TG, Norfleet AL, et al. Qualitative research-enhanced understanding of patients' beliefs: Results of focus groups with low-income, urban, African American adults with asthma. *Journal of Allergy and Clinical Immunology* 2003;111(5):967-73. doi: https://doi.org/10.1067/mai.2003.1459
22. Remien RH, Hirky AE, Johnson MO, et al. Adherence to Medication Treatment: A Qualitative Study of Facilitators and Barriers Among a Diverse Sample of HIV+ Men and Women in Four U.S. Cities. *AIDS and Behavior* 2003;7(1):61-72. doi: 10.1023/A:1022513507669
23. Hardcastle SJ, Legge E, Laundy CS, et al. Patients' Perceptions and Experiences of Familial Hypercholesterolemia, Cascade Genetic Screening and Treatment. *International Journal of Behavioral Medicine* 2015;22(1):92-100. doi: 10.1007/s12529-014-9402-x
24. Frich JC, Malterud K, Fugelli, et al. Experiences of guilt and shame in patients with familial hypercholesterolemia: a qualitative interview study. *Patient Education & Counseling* 2007;69(1-3):108-13.
25. Sliwinski SK, Gooding H, de Ferranti S, et al. Transitioning from pediatric to adult health care with familial hypercholesterolemia: Listening to young adult and parent voices. *Journal of Clinical Lipidology* 2017;11(1):147-59. doi: https://doi.org/10.1016/j.jacl.2016.11.001
26. Weiner K, Durrington PN. Patients' understandings and experiences of familial hypercholesterolemia. *Community genetics* 2008;11(5):273-82. doi: 10.1159/000121398 [published Online First: 2008/05/22]
27. Finfgeld-Connett D. Generalizability and transferability of meta-synthesis research findings. *Journal of Advanced Nursing* 2010;66(2):246-54. doi: 10.1111/j.1365-2648.2009.05250.x
28. Harris JL, Booth A, Cargo M, et al. Cochrane Qualitative and Implementation Methods Group guidance series—paper 2: methods for question formulation, searching, and protocol development for qualitative evidence synthesis. *Journal of Clinical Epidemiology* 2018;97:39-48. doi: https://doi.org/10.1016/j.jclinepi.2017.10.023
29. Sheldon TA. Making evidence synthesis more useful for management and policy-making. *Journal of health services research & policy* 2005;10(1_suppl):1-5. doi: 10.1258/1355819054308521
30. Ring NA, Ritchie K, Mandava L, et al. A guide to synthesising qualitative research for researchers undertaking health technology assessments and systematic reviews, 2011.
31. Wells M, Williams B, Firnigl D, et al. Supporting 'work-related goals' rather than 'return to work' after cancer? A systematic review and meta-synthesis of 25 qualitative studies. *Psycho-oncology* 2013;22(6):1208-19. doi: 10.1002/pon.3148 [published Online First: 2012/08/14]
32. Thomas J, Sutcliffe K, Harden A, et al. Children and Healthy Eating: A Systematic Review of Barriers and Facilitators2003.
33. Mechta Nielsen T, Frojk Juhl M, Feldt-Rasmussen B, et al. Adherence to medication in patients with chronic kidney disease: a systematic review of qualitative research. *Clinical kidney journal* 2018;11(4):513-27. doi: 10.1093/ckj/sfx140 [published Online First: 2018/08/11]
34. Ma Q, Tso LS, Rich ZC, et al. Barriers and facilitators of interventions for improving antiretroviral therapy adherence: a systematic review of global qualitative evidence. *Journal of the International AIDS Society* 2016;19(1):21166. doi: 10.7448/ias.19.1.21166 [published Online First: 2016/10/21]
35. Munro SA, Lewin SA, Smith HJ, et al. Patient adherence to tuberculosis treatment: a systematic review of qualitative research. *PLoS medicine* 2007;4(7):e238. doi: 10.1371/journal.pmed.0040238 [published Online First: 2007/08/07]
36. Langlois EV, Tuncalp, O., Norris, S.L., Askew, I., and Ghaffar, A. . Qualitative evidence to improve guidelines and health decision-making. *Bulletin of the World Health Organization* 2018 96(79-79A) doi: http://dx.doi.org/10.2471/BLT.17.206540
37. Kinnear FJ, Perry R, Searle A, et al. How do the experiences and beliefs of adults and children with heterozygous familial hypercholesterolaemia influence their adherence to treatment? A systematic review of qualitative evidence protocol. *Syst Rev* 2018;7(1):120. doi: 10.1186/s13643-018-0793-7 [published Online First: 2018/08/17]

For peer review only - http://bmjopen.bmj.com/site/about/guidelines.xhtml

38. Tong A, Flemming K, McInnes E, et al. Enhancing transparency in reporting the synthesis of qualitative research: ENTREQ. *BMC medical research methodology* 2012;12(1):181. doi: 10.1186/1471-2288-12-181
39. Programme CAS. CASP Qualitative Research Checklist, 2017.
40. Thomas J, Harden A. Methods for the thematic synthesis of qualitative research in systematic reviews. *BMC medical research methodology* 2008;8(1):45. doi: 10.1186/1471-2288-8-45
41. Noyes J, Booth A, Flemming K, et al. Cochrane Qualitative and Implementation Methods Group guidance series-paper 3: methods for assessing methodological limitations, data extraction and synthesis, and confidence in synthesized qualitative findings. *J Clin Epidemiol* 2018;97:49-58. doi: 10.1016/j.jclinepi.2017.06.020 [published Online First: 2017/12/17]
42. Barnett-Page E, Thomas J. Methods for the synthesis of qualitative research: a critical review. *BMC medical research methodology* 2009;9:59. doi: 10.1186/1471-2288-9-59 [published Online First: 2009/08/13]
43. Carroll C, Booth A. Quality assessment of qualitative evidence for systematic review and synthesis: Is it meaningful, and if so, how should it be performed? *Research Synthesis Methods* 2014;6(2):149-54. doi: 10.1002/jrsm.1128
44. Carroll C, Booth A, Lloyd-Jones M. Should we exclude inadequately reported studies from qualitative systematic reviews? An evaluation of sensitivity analyses in two case study reviews. *Qualitative health research* 2012;22(10):1425-34. doi: 10.1177/1049732312452937 [published Online First: 2012/08/07]
45. Craig JC, Tong A, Strippoli GFM, et al. A guide to reading and using systematic reviews of qualitative research. *Nephrology Dialysis Transplantation* 2014;31(6):897-903. doi: 10.1093/ndt/gfu354 %J Nephrology Dialysis Transplantation
46. Frich JC. Living with genetic risk of coronary heart disease: A qualitative interview study of patients with familial hypercholesterolemia. *Thesis* 2007
47. Urke EB. Young adults with familial hypercholesterolemia and their thoughts on lifelong treatment. A qualitative interview study. . *Master Thesis* 2016
48. Weiner K. Patient and Professional COnstructions of Familial Hypercholesterolaemia and Heart Disease: Testing the Limits of the Geneticisation Thesis. . *PhD Thesis* 2006
49. Agard A, Bolmsjo IA, Hermeren G, et al. Familial hypercholesterolemia: ethical, practical and psychological problems from the perspective of patients. *Patient Educ Couns* 2005;57(2):162-7. doi: 10.1016/j.pec.2004.05.010 [published Online First: 2005/05/25]
50. DeAngelis EJ, McIntosh S, Ahmed CD, et al. Familial hypercholesterolaemia patient–determined themes for community-engaged research. *Health Education Journal* 2017;77(3):293-302. doi: 10.1177/0017896917745567
51. Hallowell N, Jenkins N, Douglas M, et al. A qualitative study of patients' perceptions of the value of molecular diagnosis for familial hypercholesterolemia (FH). *Journal of Community Genetics* 2017;8(1):45-52. doi: 10.1007/s12687-016-0286-0
52. Jenkins N, Lawton J, Douglas M, et al. Inter-embodiment and the experience of genetic testing for familial hypercholesterolaemia. *Sociology of Health and Illness* 2013;35(4):529-43.
53. Jenkins N, Lawton J, Douglas M, et al. How do index patients participating in genetic screening programmes for familial hypercholesterolemia (FH) interpret their DNA results? A UK-based qualitative interview study. *Patient Education and Counseling* 2013;90(3):372-77. doi: https://doi.org/10.1016/j.pec.2011.09.002
54. Hollands GJ, Armstrong D, Macfarlane A, et al. Patient accounts of diagnostic testing for familial hypercholesterolaemia: comparing responses to genetic and non-genetic testing methods. *BMC Medical Genetics* 2012;13:87-87. doi: 10.1186/1471-2350-13-87
55. Hollman G, Ek A, Olsson AG, et al. The meaning of quality of life among patients with familial hypercholesterolemia. *Journal of Cardiovascular Nursing* 2004;19(4):243-50.
56. Keenan KF, Finnie RM, Simpson WG, et al. Parents' views of genetic testing and treatment of familial hypercholesterolemia in children: a qualitative study. *Journal of Community Genetics* 2018 doi: 10.1007/s12687-018-0373-5
57. Kirkegaard P, Edwards A, Risor MB, et al. Risk of cardiovascular disease? A qualitative study of risk interpretation among patients with high cholesterol. *BMC Fam Pract* 2013;14:137. doi: 10.1186/1471-2296-14-137 [published Online First: 2013/09/18]
58. Mackie TI, Tse LL, de Ferranti SD, et al. Treatment decision making for adolescents with familial hypercholesterolemia: Role of family history and past experiences. *Journal of Clinical Lipidology* 2015;9(4):583-93.e3. doi: https://doi.org/10.1016/j.jacl.2015.04.008

For peer review only - http://bmjopen.bmj.com/site/about/guidelines.xhtml

59. Meulenkamp TM, Tibben A, Mollema ED, et al. Predictive genetic testing for cardiovascular diseases: Impact on carrier children. *American Journal of Medical Genetics, Part A* 2008;146(24):3136-46.
60. Mortensen GL, Madsen IB, Kruse C, et al. Familial hypercholesterolaemia reduces the quality of life of patients not reaching treatment targets. *Danish medical journal* 2016;63(5) [published Online First: 2016/04/30]
61. Senior V, Smith JA, Michie S, et al. Making sense of risk: An interpretive phenomenological analysis of vulnerability to heart disease. *Special Issue: Interpretive phenomenological analysis and the new genetics* 2002;7(2):157-68. doi: http://dx.doi.org/10.1177/1359105302007002455
62. Frich JC, Ose L, Malterud K, et al. Perceived vulnerability to heart disease in patients with familial hypercholesterolemia: a qualitative interview study. *Annals of family medicine* 2006;4(3):198-204. doi: 10.1370/afm.529 [published Online First: 2006/06/01]
63. Frich JC, Malterud K, Fugelli P. How do patients at risk portray candidates for coronary heart disease? A qualitative interview study. *Scandinavian Journal of Primary Health Care* 2007;25(2):112-16.
64. Weiner K. The tenacity of the coronary candidate: how people with familial hypercholesterolaemia construct raised cholesterol and coronary heart disease. *Health (London, England : 1997)* 2009;13(4):407-27. doi: 10.1177/1363459309103915 [published Online First: 2009/06/06]
65. Weiner K. Exploring genetic responsibility for the self, family and kin in the case of hereditary raised cholesterol. *Social Science & Medicine* 2011;72(11):1760-67. doi: 10.1016/j.socscimed.2010.03.053
66. Claassen L, Henneman L, Kindt I, et al. Perceived Risk and Representations of Cardiovascular Disease and Preventive Behaviour in People Diagnosed with Familial Hypercholesterolemia: A Cross-sectional Questionnaire Study. *Journal of Health Psychology* 2010;15(1):33-43. doi: 10.1177/1359105309345170
67. van Maarle MC, Stouthard ME, Bonsel GJ. Risk perception of participants in a family-based genetic screening program on familial hypercholesterolemia. *American journal of medical genetics Part A* 2003;116a(2):136-43. doi: 10.1002/ajmg.a.10061 [published Online First: 2002/12/21]
68. Claassen L, Henneman L, van der Weijden T, et al. Being at risk for cardiovascular disease: Perceptions and preventive behavior in people with and without a known genetic predisposition. *Psychology, Health & Medicine* 2012;17(5):511-21. doi: 10.1080/13548506.2011.644246
69. Muir LA, George PM, Whitehead L. Using the experiences of people with familial hypercholesterolaemia to help reduce the risk of cardiovascular disease: a qualitative systematic review. *Journal of Advanced Nursing* 2012;68(9):1920-32. doi: 10.1111/j.1365-2648.2012.05957.x
70. Andersen LK, Jensen HK, Juul S, et al. Patients' attitudes toward detection of heterozygous familial hypercholesterolemia. *Archives of internal medicine* 1997;157(5):553-60. [published Online First: 1997/03/10]
71. Tonstad S, Novik TS, Vandvik IH. Psychosocial function during treatment for familial hypercholesterolemia. *Pediatrics* 1996;98(2 Pt 1):249-55. [published Online First: 1996/08/01]
72. de Jongh S, Kerckhoffs MC, Grootenhuis MA, et al. Quality of life, anxiety and concerns among statin-treated children with familial hypercholesterolaemia and their parents. *Acta Pædiatrica* 2003;92(9):1096-101. doi: 10.1111/j.1651-2227.2003.tb02584.x
73. van Maarle MC, Stouthard ME, Bonsel GJ. Quality of life in a family based genetic cascade screening programme for familial hypercholesterolaemia: a longitudinal study among participants. *Journal of medical genetics* 2003;40(1):e3. [published Online First: 2003/01/15]
74. van Maarle MC, Stouthard ME, Marang-van de Mheen PJ, et al. How disturbing is it to be approached for a genetic cascade screening programme for familial hypercholesterolaemia? Psychological impact and screenees' views. *Community genetics* 2001;4(4):244-52. doi: 10.1159/000064200 [published Online First: 2003/05/20]
75. Hyttinen L, Kekäläinen P, Vuorio AF, et al. Health-related quality of life in elderly patients with familial hypercholesterolemia. *International Journal of Technology Assessment in Health Care* 2008;24(2):228-34. doi: 10.1017/S0266462308080318
76. Akioyamen LE, Genest J, Shan SD, et al. Anxiety, depression, and health-related quality of life in heterozygous familial hypercholesterolemia: A systematic review and meta-analysis. *Journal of psychosomatic research* 2018;109:32-43. doi: 10.1016/j.jpsychores.2018.03.170 [published Online First: 2018/05/19]
77. Senior V, Marteau TM, Weinman J. Self-Reported Adherence to Cholesterol-Lowering Medication in Patients with Familial Hypercholesterolaemia: The Role of Illness Perceptions. *Cardiovascular Drugs and Therapy* 2004;18(6):475-81. doi: 10.1007/s10557-004-6225-z

For peer review only - http://bmjopen.bmj.com/site/about/guidelines.xhtml

78. Galema-Boers A, Langendonk JG, Roeters Van Lennep J, et al. Developing a prediction model to identify noncompliance in a nurse-led familial hypercholesterolemia clinic. *European Heart Journal* 2011;1):66.
79. Galema-Boers JM, Lenzen MJ, van Domburg RT, et al. Predicting non-adherence in patients with familial hypercholesterolemia. *European journal of clinical pharmacology* 2014;70(4):391-7. doi: 10.1007/s00228-013-1640-3 [published Online First: 2014/01/23]
80. Braamskamp MJAM, Kusters DM, Avis HJ, et al. Long-Term Statin Treatment in Children with Familial Hypercholesterolemia: More Insight into Tolerability and Adherence. *Paediatric Drugs* 2015;17(2):159-66. doi: 10.1007/s40272-014-0116-y
81. Retterstol K, Mundal L, Igland J, et al. Incidence of various types of atherosclerotic disease in patients with genotyped familial hypercholesterolemia. *Atherosclerosis* 2017;263:e26. doi: https://doi.org/10.1016/j.atherosclerosis.2017.06.106
82. Hopkins PN. Putting Into Perspective the Hazards of Untreated Familial Hypercholesterolemia. *Journal of the American Heart Association* 2017;6(6)
83. deGoma EM, Ahmad ZS, O'Brien EC, et al. Treatment Gaps in Adults With Heterozygous Familial Hypercholesterolemia in the United States: Data From the CASCADE-FH Registry. *Circulation Cardiovascular genetics* 2016;9(3):240-9. doi: 10.1161/circgenetics.116.001381 [published Online First: 2016/03/26]
84. Saltijeral A, Perez de Isla L, Alonso R, et al. Attainment of LDL Cholesterol Treatment Goals in Children and Adolescents With Familial Hypercholesterolemia. The SAFEHEART Follow-up Registry. *Revista espanola de cardiologia (English ed)* 2017;70(6):444-50. doi: 10.1016/j.rec.2016.10.010 [published Online First: 2016/12/04]
85. Humphries SE, Cooper J, Dale P, et al. The UK Paediatric Familial Hypercholesterolaemia Register: Statin-related safety and 1-year growth data. *J Clin Lipidol* 2018;12(1):25-32. doi: 10.1016/j.jacl.2017.11.005 [published Online First: 2017/12/07]
86. Galema-Boers AM, Lenzen MJ, Engelkes SR, et al. Cardiovascular risk in patients with familial hypercholesterolemia using optimal lipid-lowering therapy. *Journal of Clinical Lipidology* 2018;12(2):409-16. doi: https://doi.org/10.1016/j.jacl.2017.12.014
87. Pérez de Isla L, Alonso R, Mata N, et al. Predicting Cardiovascular Events in Familial Hypercholesterolemia. *Circulation* 2017;135(22):2133.
88. Senior V, Marteau T, Weinman J. Perceptions of control over heart disease in people with an inherited predisposition to raised cholesterol. *Psychology, Health & Medicine* 2005;10(1):16-30. doi: 10.1080/13548500512331315334
89. Senior V, Marteau TM. Causal attributions for raised cholesterol and perceptions of effective risk-reduction: Self-regulation strategies for an increased risk of coronary heart disease. *Psychology and Health* 2007;22(6):699-717. doi: 10.1080/14768320601020253
90. Tonstad S. Familial hypercholesterolaemia: a pilot study of parents' and children's concerns. *Acta paediatrica (Oslo, Norway : 1992)* 1996;85(11):1307-13. [published Online First: 1996/11/01]
91. Avis HJ, Kusters DM, Vissers MN, et al. Follow-Up of Children Diagnosed with Familial Hypercholesterolemia in a National Genetic Screening Program. *The Journal of Pediatrics* 2012;161(1):99-103. doi: https://doi.org/10.1016/j.jpeds.2011.12.037
92. Benson G, Witt DR, VanWormer JJ, et al. Medication adherence, cascade screening, and lifestyle patterns among women with hypercholesterolemia: Results from the WomenHeart survey. *J Clin Lipidol* 2016;10(4):937-43. doi: 10.1016/j.jacl.2016.03.012 [published Online First: 2016/09/01]
93. Collins R, Reith C, Emberson J, et al. Interpretation of the evidence for the efficacy and safety of statin therapy. *The Lancet* 2016;388(10059):2532-61. doi: 10.1016/S0140-6736(16)31357-5
94. Vuorio A, Kuoppala J, Kovanen PT, et al. Statins for children with familial hypercholesterolemia. *Cochrane Database Syst Rev* 2017;7:Cd006401. doi: 10.1002/14651858.CD006401.pub4 [published Online First: 2017/07/08]
95. Lozano P, Henrikson NB, Morrison CC, et al. Lipid Screening in Childhood and Adolescence for Detection of Multifactorial Dyslipidemia: Evidence Report and Systematic Review for the US Preventive Services Task Force. *Jama* 2016;316(6):634-44. doi: 10.1001/jama.2016.6423 [published Online First: 2016/08/18]
96. Radaelli G, Sausen G, Cesa CC, et al. Statin Treatments And Dosages In Children With Familial Hypercholesterolemia: Meta-Analysis. *Arquivos brasileiros de cardiologia* 2018 doi: 10.5935/abc.20180180 [published Online First: 2018/10/27]

For peer review only - http://bmjopen.bmj.com/site/about/guidelines.xhtml

97. Hollman G, Olsson AG, Ek AC. Disease knowledge and adherence to treatment in patients with familial hypercholesterolemia. *The Journal of cardiovascular nursing* 2006;21(2):103-8. [published Online First: 2006/04/08]
98. van Maarle MC, Stouthard MEA, de Mheen PJM-v, et al. Follow up after a family based genetic screening programme for familial hypercholesterolaemia: is screening alone enough? *BMJ : British Medical Journal* 2002;324(7350):1367-68.
99. Kinnear FJ, Lithander FE, Hamilton-Shield JP, et al. What dietetic support is available to patients in outpatient lipid clinics in the uk? *Atherosclerosis Supplements* 2018;34:e5. doi: 10.1016/j.atherosclerosissup.2018.07.029
100. Umans-Eckenhausen MA, Defesche JC, van Dam MJ, et al. Long-term compliance with lipid-lowering medication after genetic screening for familial hypercholesterolemia. *Archives of internal medicine* 2003;163(1):65-8. [published Online First: 2003/01/14]
101. Jones LK, Kulchak Rahm A, Manickam K, et al. Healthcare Utilization and Patients' Perspectives After Receiving a Positive Genetic Test for Familial Hypercholesterolemia. *Circulation Genomic and precision medicine* 2018;11(8):e002146. doi: 10.1161/circgen.118.002146 [published Online First: 2018/10/26]
102. Chisnell J, Marshall T, Hyde C, et al. A content analysis of the representation of statins in the British newsprint media. *BMJ Open* 2017;7(8)
103. Tonstad S, Sivertsen M. Food dislikes and dietary intake in children with familial hypercholesterolemia and controls. *Scandinavian Journal of Nutrition/Naringsforskning* 1996;40(4):146-50.
104. Stender S, Skovby F, Haraldsdottir J, et al. Cholesterol-lowering diets may increase the food costs for Danish children. A cross-sectional study of food costs for Danish children with and without familial hypercholesterolaemia. *European Journal of Clinical Nutrition* 1993;47(11):776-86.
105. Dixon LB, Tershakovec AM, McKenzie J, et al. Diet quality of young children who received nutrition education promoting lower dietary fat. *Public health nutrition* 2000;3(4):411-6. [published Online First: 2001/01/03]
106. Molven I, Retterstol K, Andersen LF, et al. Children and young adults with familial hypercholesterolaemia (FH) have healthier food choices particularly with respect to dietary fat sources compared with non-FH children. *Journal of nutritional science* 2013;2:e32. doi: https://dx.doi.org/10.1017/jns.2013.27
107. West RJ. FAMILIAL HYPERCHOLESTEROLAEMIA. FACTORS INFLUENCING DRUG COMPLIANCE. *Pediatric Research* 1980;14:1423. doi: 10.1203/00006450-198012000-00089
108. Marteau T, Senior V, Humphries SE, et al. Psychological impact of genetic testing for familial hypercholesterolemia within a previously aware population: a randomized controlled trial. *American journal of medical genetics Part A* 2004;128a(3):285-93. doi: 10.1002/ajmg.a.30102 [published Online First: 2004/06/25]
109. Lee S, Akioyamen LE, Aljenedil S, et al. Genetic testing for familial hypercholesterolemia: Impact on diagnosis, treatment and cardiovascular risk. *Eur J Prev Cardiol* 2019:2047487319829746. doi: 10.1177/2047487319829746 [published Online First: 2019/02/14]
110. Ramli A, Ahmad NS, Paraidathathu T. Medication adherence among hypertensive patients of primary health clinics in Malaysia. *Patient preference and adherence* 2012;6:613-22. doi: 10.2147/PPA.S34704
111. Polonsky WH, Henry RR. Poor medication adherence in type 2 diabetes: recognizing the scope of the problem and its key contributors. *Patient preference and adherence* 2016;10:1299-307. doi: 10.2147/PPA.S106821
112. Leslie KH, McCowan C, Pell JP. Adherence to cardiovascular medication: a review of systematic reviews. *Journal of Public Health* 2018;41(1):e84-e94. doi: 10.1093/pubmed/fdy088 %J Journal of Public Health
113. Sud A, Kline-Rogers EM, Eagle KA, et al. Adherence to Medications by Patients After Acute Coronary Syndromes. *Annals of Pharmacotherapy* 2005;39(11):1792-97. doi: 10.1345/aph.1G249
114. Alm-Roijer C, Stagmo M, Udén G, et al. Better Knowledge Improves Adherence to Lifestyle Changes and Medication in Patients with Coronary Heart Disease. *European Journal of Cardiovascular Nursing* 2004;3(4):321-30. doi: 10.1016/j.ejcnurse.2004.05.002
115. Tiv M, Viel JF, Mauny F, et al. Medication adherence in type 2 diabetes: the ENTRED study 2007, a French Population-Based Study. *PLoS One* 2012;7(3):e32412. doi: 10.1371/journal.pone.0032412 [published Online First: 2012/03/10]
116. Broadbent E, Donkin L, Stroh JC. Illness and treatment perceptions are associated with adherence to medications, diet, and exercise in diabetic patients. *Diabetes care* 2011;34(2):338-40. doi: 10.2337/dc10-1779 [published Online First: 2011/01/29]

For peer review only - http://bmjopen.bmj.com/site/about/guidelines.xhtml

117. Pollack MF, Purayidathil FW, Bolge SC, et al. Patient-reported tolerability issues with oral antidiabetic agents: Associations with adherence; treatment satisfaction and health-related quality of life. *Diabetes Research and Clinical Practice* 2010;87(2):204-10. doi: https://doi.org/10.1016/j.diabres.2009.11.023
118. Miller TA, Dimatteo MR. Importance of family/social support and impact on adherence to diabetic therapy. *Diabetes Metab Syndr Obes* 2013;6:421-26. doi: 10.2147/DMSO.S36368
119. Rintala T-M, Jaatinen P, Paavilainen E, et al. Interrelation Between Adult Persons With Diabetes and Their Family: A Systematic Review of the Literature. *Journal of Family Nursing* 2013;19(1):3-28. doi: 10.1177/1074840712471899

For peer review only

Figure 1: PRISMA Flow diagram

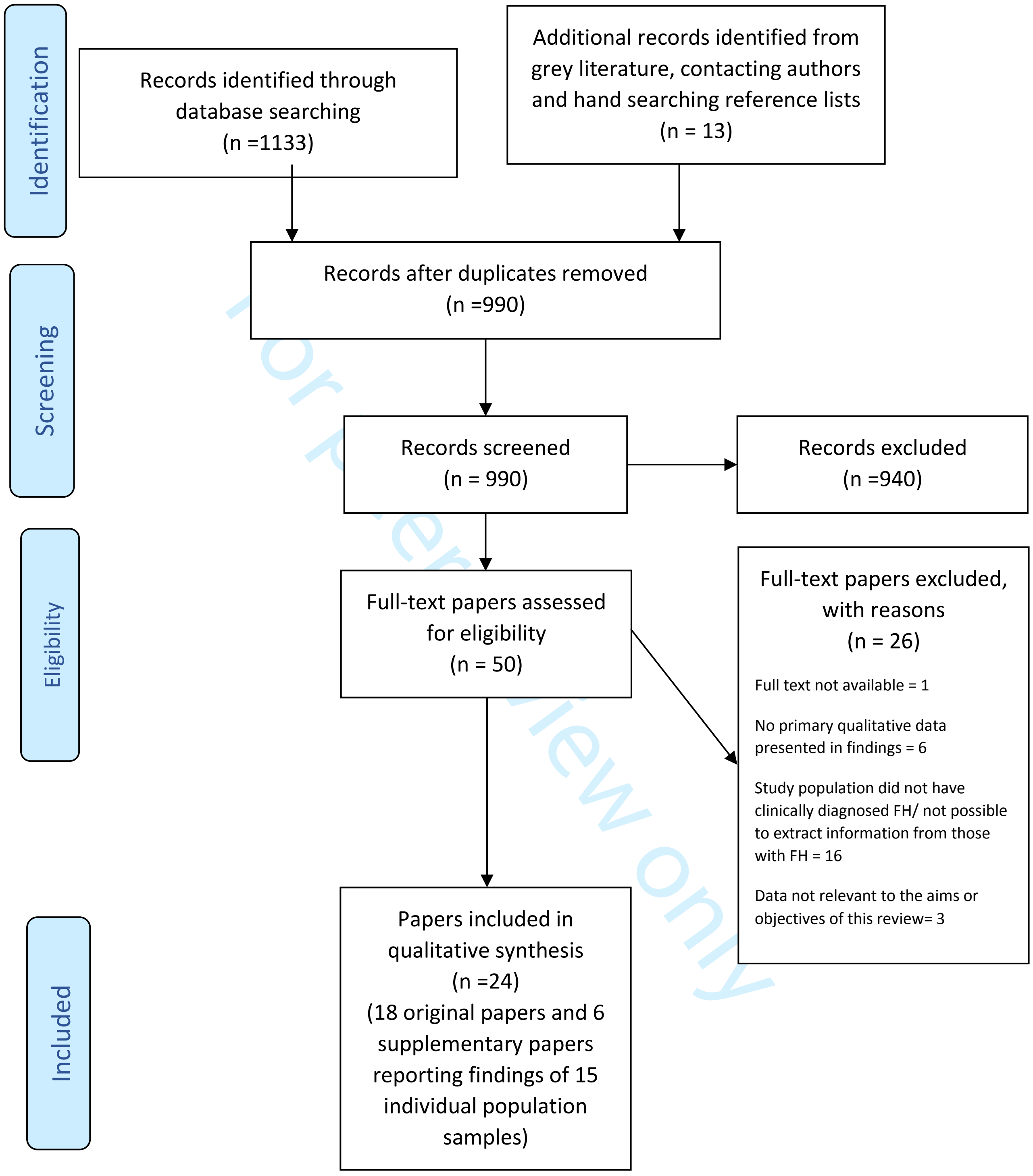

Figure 2: Thematic schema illustrating influence of analytical themes upon treatment adherence

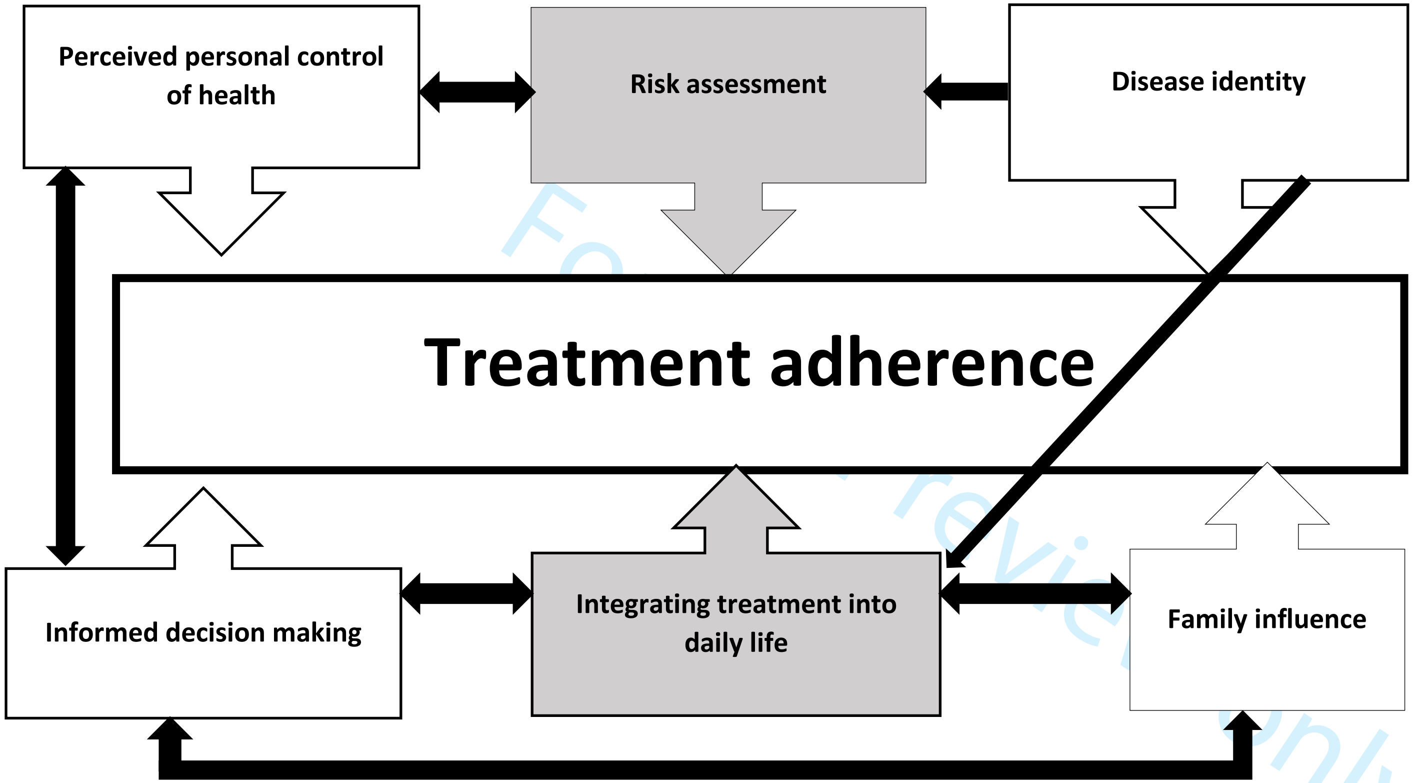

For peer review only

Supplementary File 1: Deviations from protocol

Stated in protocol	What we did	Rationale for deviation
'Only studies in which the full text is available in English will be eligible for inclusion'	We did not place any limits upon language of included papers	We aimed to overcome the recognised restrictions of individual qualitative study findings, by gathering and examining a wide range of patient perceptions and experiences. After an initial scope of the available evidence base in this population group, it was apparent that the number of potential papers to be retrieved would be manageable by the research team. Therefore, the decision was made to remove this exclusion criteria, in order to identify all relevant evidence in line with the comprehensive searching approach to be taken in this review. This is in line with available guidance which advises that language filter decisions should be made in reference to the aims of the review.[28]
'The participants include individuals aged ≥ 10 years'	We did not place any limits upon age of included participants.	The database searching retrieved a paper reporting findings from a sample which included children aged 8 years. As it was not possible to extract the data from only participants aged 10 years and older, using the original inclusion criteria the paper would have to be excluded from the synthesis. This paper was one of only 3 papers retrieved that reported findings from samples including children, therefore the findings were perceived to be very valuable to the synthesis. Children are often diagnosed with FH before the age of 10, and U.K. and international guidance advise treatment with lifestyle advice, with lipid lowering therapy to be implemented when they reach a suitable age.[11] Furthermore, it is stated in the NICE guidelines that lipid lowering drug treatment should be commenced by the age of 10 and statin therapy can be considered at 8-10 years of age.
'Both stages of data extraction will be carried out independently by two reviewers (AS, FJK)…'	First stage of data extraction (study details) was carried out by two reviewers (JC, FJK) and second stage (study findings) by two reviewers (AS, FJK).	This was to split work between review members.
'The two reviewers (FJK, AS) will then work in collaboration to develop initial descriptive themes and categories based upon the raw data…'	Three reviewers (FJK, AS, EW) worked in collaboration for the second two stages of thematic synthesis.	EW joined review team after publication of protocol. We felt having a further expert opinion from a health psychologist would improve the synthesis output.
'The findings are intended to be used in the development of future intervention or guidelines….'	The findings are presented with a focus on informing clinical practice	The findings were interpreted to be of particular importance to clinical practice. While the findings are still useful to intervention and guideline development, this paper will focus upon their application in a clinical setting,

For peer review only - http://bmjopen.bmj.com/site/about/guidelines.xhtml

Supplementary File 2- ENTREQ reporting guidelines checklist

ENTREQ: Enhancing transparency in reporting the synthesis of qualitative research [38]

No	Item	Guide and description	Reported on page #
**1**	Aim	State the research question the synthesis addresses.	Page 4 & 5
**2**	Synthesis methodology	Identify the synthesis methodology or theoretical framework which underpins the synthesis, and describe the rationale for choice of methodology *(e.g. meta-ethnography, thematic synthesis, critical interpretive synthesis, grounded theory synthesis, realist synthesis, meta-aggregation, meta-study, framework synthesis).*	Protocol and page 7
**3**	Approach to searching	Indicate whether the search was pre-planned (*comprehensive search strategies to seek all available studies)* or iterative (*to seek all available concepts until they theoretical saturation is achieved).*	Protocol, page 6 and supplementary file 3
**4**	Inclusion criteria	Specify the inclusion/exclusion criteria *(e.g. in terms of population, language, year limits, type of publication, study type).*	Protocol and page 6
**5**	Data sources	Describe the information sources used (e.g. *electronic databases (MEDLINE, EMBASE, CINAHL, psycINFO, Econlit), grey literature databases (digital thesis, policy reports), relevant organisational websites, experts, information specialists, generic web searches (Google Scholar) hand searching, reference lists)* and when the searches conducted; provide the rationale for using the data sources.	Protocol, page 6 and supplementary file 3
**6**	Electronic Search strategy	Describe the literature search *(e.g. provide electronic search strategies with population terms, clinical or health topic terms, experiential or social phenomena related terms, filters for qualitative research, and search limits).*	Protocol, page 6 and supplementary file 3
**7**	Study screening methods	Describe the process of study screening and sifting *(e.g. title, abstract and full text review,*	Protocol and page 7

For peer review only - http://bmjopen.bmj.com/site/about/guidelines.xhtml

No	Item	Guide and description	Reported on page #
		number of independent reviewers who screened studies).	
**8**	Study characteristics	Present the characteristics of the included studies *(e.g. year of publication, country, population, number of participants, data collection, methodology, analysis, research questions).*	Page 8, table 1 and supplementary file 4
**9**	Study selection results	Identify the number of studies screened and provide reasons for study exclusion *(e,g, for comprehensive searching, provide numbers of studies screened and reasons for exclusion indicated in a figure/flowchart; for iterative searching describe reasons for study exclusion and inclusion based on modifications t the research question and/or contribution to theory development).*	Figure 1 and page 8
**10**	Rationale for appraisal	Describe the rationale and approach used to appraise the included studies or selected findings *(e.g. assessment of conduct (validity and robustness), assessment of reporting (transparency), assessment of content and utility of the findings).*	Protocol, pages 6-7 and supplementary file 4
**11**	Appraisal items	State the tools, frameworks and criteria used to appraise the studies or selected findings *(e.g. Existing tools: CASP, QARI, COREQ, Mays and Pope*[25]*; reviewer developed tools; describe the domains assessed: research team, study design, data analysis and interpretations, reporting).*	Protocol, pages 6 and supplementary file 4
**12**	Appraisal process	Indicate whether the appraisal was conducted independently by more than one reviewer and if consensus was required.	Protocol, pages 6-7, supplementary file 4
**13**	Appraisal results	Present results of the quality assessment and indicate which articles, if any, were weighted/excluded based on the assessment and give the rationale.	Page 12, Table 1 & supplementary file 4

For peer review only - http://bmjopen.bmj.com/site/about/guidelines.xhtml

No	Item	Guide and description	Reported on page #
**14**	Data extraction	Indicate which sections of the primary studies were analysed and how were the data extracted from the primary studies? *(e.g. all text under the headings "results /conclusions" were extracted electronically and entered into a computer software).*	Protocol and page 7
**15**	Software	State the computer software used, if any.	Protocol and page 7
**16**	Number of reviewers	Identify who was involved in coding and analysis.	Page 7 and supplementary file 5
**17**	Coding	Describe the process for coding of data *(e.g. line by line coding to search for concepts).*	Page 7 and supplementary file 5
**18**	Study comparison	Describe how were comparisons made within and across studies *(e.g. subsequent studies were coded into pre-existing concepts, and new concepts were created when deemed necessary).*	Supplementary file 5
**19**	Derivation of themes	Explain whether the process of deriving the themes or constructs was inductive or deductive.	Supplementary file 5
**20**	Quotations	Provide quotations from the primary studies to illustrate themes/constructs, and identify whether the quotations were participant quotations of the author's interpretation.	Table 2 and pages 13-16
**21**	Synthesis output	Present rich, compelling and useful results that go beyond a summary of the primary studies (e.g. *new interpretation, models of evidence, conceptual models, analytical framework, development of a new theory or construct).*	Table 4 and pages 22-25

3

Supplementary File 3- Full details of search strategy

Full details are available in the published protocol[37] but are detailed briefly below.

Search Strategy

MEDLINE, Embase, PsycINFO (via OVID), Cochrane library and CINAHL databases were searched from inception to 05/09/2018. We used a validated qualitative search filter[(121)] and population specific search terms. The search strategy that was used in MEDLINE is displayed in Appendix 1. The OpenGrey database and specialist websites (HEART UK, British Heart Foundation, The FH Foundation and The Simon Broome Register) were also searched up until 05/09/2018. The reference lists of the 50 papers taken to the full text screening stage were also hand searched. When only an abstract was available, the lead author was conducted in attempt to retrieve the full text. When contacting the lead authors of the included papers as part of the quality appraisal stage, enquiries were also made about any unpublished work.

Appendix 1: Search Strategy used in MEDLINE 05/09/2018

1. (familial adj1 hypercholesterolemia).ti,ab, kf.

2. (familial adj1 hypercholesterolaemia).ti,ab, kf.

3. (inherit* adj1 high adj1 cholesterol).ti,ab, kf.

4. *Hypercholesterolemia/ge [Genetics]

5. 1 or 2 or 3 or 4

6. interview*.ti,ab.

7. exp. Interviews/

8. experience*.tw.

9. qualitative.ti,ab.

10. 6 or 7 or 8 or 9

11. 5 and 10

For peer review only - http://bmjopen.bmj.com/site/about/guidelines.xhtml

<u>Supplementary File 4- Quality appraisal methodology and results</u>

Methodology

The CASP tool, endorsed by the Cochrane Collaboration,[41] asks 10 questions relating to the rigour of the methodology used, quality of reporting and relevance of findings. To ensure comprehensive evaluation of methodological quality, these questions were answered with further consideration of 12 criteria produced by an expert panel.[112] As the purpose of the quality appraisal was to determine the methodological strengths and limitations of studies included in the synthesis, the lead authors of each paper were contacted to obtain further information in an attempt to overcome the recognised issued of poor reporting in qualitative research. Information from multiple papers involving the same sample was pooled when appropriate. Each author was given 1 month to respond. Two reviewers (AS, FK) independently appraised each study, assigning a rating of 0, 1 or 2 for each question which reflected the extent to which the obtained information from paper and author answered the criteria (0=not addressed, 1=partially addressed, 2=fully addressed). The reviewers then met to come to a consensus of individual and total scores, resolving differences through discussion. The reviewers then decided upon threshold for low, medium and high rated quality that they felt adequately captured the quality of the included papers.

Summary of results

Table 1 displays the CASP score breakdowns for each paper. Table 2 displays further details of the methodological limitations and transferability considerations of each included paper.

For peer review only - http://bmjopen.bmj.com/site/about/guidelines.xhtml

Table 1: CASP appraisal scores of included studies

Sample number	Reference	Was there a clear statement of research aims?	Is qualitative methodology appropriate?	Was the research design appropriate to address the aims of the research?	Was the recruitment strategy appropriate to the aims of the research?	Were the data collected in a way that addressed the research issue?	Has the relationship between researcher and participants been adequately considered?	Have ethical issues been taken into consideration?	Was the data analysis sufficiently rigorous?	Is there a clear statement of findings?	Is the research valuable?	Overall score (out of 20)	Did author provide further information?
**1**	Agard et al, 2005[49]	2	2	2	2	2	0	1	0	1	1	**13**	**NO**
**2**	DeAngelis et al, 2017[50]	2	2	2	1	1	1	0	1	1	2	**13**	**NO**
**3**	Frich, 2007[46]	2	2	2	2	2	2	2	2	2	2	**20**	**YES**
	Frich et al, 2006[62]	2	2	2	2	2	2	2	2	1	2	**19**	**YES**
	Frich et al, 2007[63]	2	2	2	1	2	2	2	2	2	1	**18**	**YES**
	Frich et al, 2007[24]	2	2	2	2	2	2	2	2	2	2	**20**	**YES**
**4**	Hallowell et al, 2017[51]	2	2	2	2	2	1	2	1	1	2	**17**	**YES**
	Jenkins et al, 2013[53]	2	2	2	1	2	0	2	1	1	2	**15**	**YES**
	Jenkins et al, 2013[52]	2	2	2	1	2	0	2	1	0	1	**13**	**YES**
**5**	Hardcastle et al, 2015[23]	2	2	2	2	2	1	2	1	2	2	**18**	**YES**
**6**	Hollands et al, 2012[54]	2	2	2	2	0	0	2	1	1	1	**13**	**NO**
**7**	Hollman et al, 2004[55]	2	2	2	2	2	1	2	2	2	2	**19**	**YES**
**8**	Keenan et al, 2018[56]	2	2	2	2	2	0	2	1	1	2	**16**	**YES**
**9**	Kirkegaard et al, 2014[57]	2	2	2	2	2	1	2	2	1	1	**17**	**YES**

For peer review only - http://bmjopen.bmj.com/site/about/guidelines.xhtml

**10**	Mackie et al, 2015[58]	2	2	2	2	2	2	1	2	2	2	**19**	**NO**
	Sliwinski et al, 2017[25]	2	2	2	2	2	2	1	2	2	2	**19**	**NO**
**11**	Meulenkamp et al, 2008[59]	2	2	2	2	2	1	2	2	2	2	**19**	**YES**
**12**	Mortensen et al, 2008[60]	2	2	2	1	1	0	0	1	1	1	**11**	**NO**
**13**	Urke, 2016[47]	2	2	2	2	2	2	2	2	2	2	**20**	**NO**
**14**	Weiner, 2006[48]	2	2	2	1	2	1	2	1	2	2	**18**	**YES**
	Weiner and Durrington, 2008[26]	2	2	2	1	2	1	2	1	2	2	**17**	**YES**
	Weiner, 2009[64]	2	2	2	1	2	1	2	1	1	1	**16**	**YES**
	Weiner, 2011[65]	2	2	2	1	2	1	2	1	1	2	**16**	**YES**
**15**	Senior et al, 2002[61]	2	2	2	2	1	0	0	1	1	1	**12**	**NO**

Scoring system: 0=No criteria fulfilled or can't tell; 1= some criteria fulfilled; 2= All criteria fulfilled. In reference to the criteria suggested for each question by CASP tool(53) and further criteria as described by Santiago-Delefosse et al.(122)

For peer review only - http://bmjopen.bmj.com/site/about/guidelines.xhtml

Table 2: Summary of methodological limitations and transferability considerations of the included papers

Sample number	Reference	CASP quality SCORE & rating	Methodological and reporting limitations	Transferability considerations of sample
**1**	Agard et al, 2005[49]	13 Low	Lack of details provided about the rigour of the analysis process.<br>Authors self-selected the data from interviews to transcribe.<br>Data saturation not discussed.<br>Relationship between researcher and participants was not adequately considered.<br>Credibility of findings and the limitations of study design not addressed when reporting the findings.<br>No details of informed consent or if participants were told about data confidentiality or their right to withdraw.	No sampling strategy used but sample comprised of a good range of ages, genders, history of CVD events and age of diagnosis.<br>All recruited from one clinic.<br>All from Sweden.
**2**	DeAngelis et al, 2017[50]	13 Low	Ethical issues not addressed.<br>Group meetings may have resulted in lack of representative findings as certain individuals may have dominated the conversations or individuals may have felt unable to voice their own opinions.<br>Lack of disconfirming cases presented<br>No details of informed consent or if participants were told about data confidentiality or their right to withdraw.	All very motivated and engaged individuals to volunteer for this group.<br>Many receiving apheresis treatment.
**3**	Frich, 2007[46]	20 high	Study limitations not addressed when reporting the findings.	All motivated to seek treatment as active attendees of lipid clinic.<br>Majority young (70% 10-39 years) and asymptomatic.<br>Large (40) sample size.<br>All from Norway.<br>All recruited from one lipid clinic.
	Frich et al, 2006[62]	19 High	Lack of disconfirming cases presented and discussion against the findings.	
	Frich et al, 2007[63]	18 High	Lack of disconfirming cases presented and discussion against the findings.	
	Frich et al, 2007[24]	20 High	Study limitations not addressed when reporting the findings.	
**4**	Hallowell et al, 2017[51]	17 Medium	Lack of details provided about the rigour of the analysis process<br>Relationship between researcher and participants was not adequately considered	All participants regularly attend lipid clinics and opted in for DNA testing.<br>Relatively well education (42% university education).<br>All participants from Scotland.<br>No sampling strategy used so likely not representative.<br>Half of patients from professional/skilled non-manual background.<br>Ethnicity not provided but authors state majority white British.<br>Recruited from two lipid clinics.
	Jenkins et al, 2013[53]	15 Medium	Credibility of findings and the limitations of study design not addressed when reporting the findings<br>Relationship between researcher and participants was not adequately considered.	
	Jenkins et al, 2013[52]	13 Low	Lack of details provided about the rigour of the analysis process.<br>Credibility of findings and the limitations of study design not addressed when reporting the findings.<br>Relationship between researcher and participants was not adequately considered.	
**5**	Hardcastle et al, 2015[23]	18 High	Analysis carried out by one individual only with no independent verification of themes conducted.<br>Relationship between researcher and participants was not adequately considered.	Sample not randomly selected.<br>Recruited from one clinic.<br>All live in metropolitan Perth, Australia.
**6**	Hollands et al, 2012[54]	13 Low	Lack of disconfirming cases presented and arguments against findings.<br>Data saturation not discussed.<br>Relationship between researcher and participants was not adequately considered.<br>Credibility of findings and the limitations of study design not addressed when reporting the findings.	Recruited across 11 lipid clinics.<br>All from the U.K.<br>All recently identified as being at risk of FH and during study received either clinical or DNA test results.

For peer review only - http://bmjopen.bmj.com/site/about/guidelines.xhtml

				Sample included in analysis includes participates with DNA positive and Non-DNA positive diagnosis.<br>Majority (14/19) white British.
**7**	Hollman et al, 2004[55]	19 High	Relationship between researcher and participants was not adequately considered.	All Swedish.<br>All recruited from one lipid clinic.
**8**	Keenan et al, 2018[56]	16 Medium	Credibility of findings and the limitations of study design not addressed when reporting the findings.<br>Relationship between researcher and participants was not adequately considered.<br>Analysis carried out by one individual.	All had consented to genetic testing.<br>All from Scotland.<br>All participants white, and majority highly educated.<br>Majority of participants asymptomatic.<br>Patients were self-selected from HCP who excluded participants if they felt they were too vulnerable, which included if had experienced a recent bereavement.<br>13 of parents had FH, 4 were spouses of those with FH
**9**	Kirkegaard et al, 2014[57]	17 Medium	Lack of results to support conclusions drawn.<br>Credibility of findings and the limitations of study design not addressed when reporting the findings.	All asymptomatic.<br>Only 2 FH patients and 1 relative of FH patient.
**10**	Mackie et al, 2015[58]	19 High	No details of informed consent or if participants were told about data confidentiality or their right to withdraw.	Most participants had private medical insurance, were white and all actively engaged with the healthcare system.<br>All recruited from same healthcare system.<br>All patients from Massachusetts, U.S.A.
	Sliwinski et al, 2017[25]	19 High	No details of informed consent or if participants were told about data confidentiality or their right to withdraw.	
**11**	Meulenkamp et al, 2008[59]	19 High	Relationship between researcher and participants was not adequately considered-three interviewers carried out the interviews and the potential bias this may incur was not addressed	All recruited from one health intuition.<br>All engaged with healthcare system and willing to talk about their condition.<br>11/16 were females.
**12**	Mortensen et al, 2008[60]	11 Low	Lack of details provided about the study methodology or rigour of analysis process.<br>Data saturation not discussed.<br>Relationship between researcher and participants was not adequately considered.<br>Credibility of findings and the limitations of study design not addressed when reporting the findings.<br>Ethical issues not addressed.	Half participants were reaching treatment goals, half were not.<br>All recruited from one genetic centre.<br>All Danish.<br>Only 1 female in the group of patients reaching treatment targets
**13**	Urke, 2016[47]	20 High	Coding and analysis of data was primarily independent, with the student's supervisors only overseeing it.	Sample comprised of non-attenders at clinic-not been seen for at least 2 years<br>Wide geographical spread, but all participants from Norway<br>Participants recruited from one clinic
**14**	Weiner, 2006[48]	18 High	Analysis by single researcher and potential bias not addressed<br>Data saturation not discussed.<br>Relationship between researcher and participants was not adequately considered.	Quota sampling used but all were white and majority (28/31) white British, 65% were ≥46 years old and 50% from professional occupations.<br>Participants recruited from one clinic.<br>Half self-reported experiencing some form of CHD.<br>All from North England, U.K.<br>All attended lipid clinic for at least 1 year, most for substantially longer.
	Weiner and Durrington, 2008[26]	17 Medium	Analysis by single researcher and potential bias not addressed.<br>Data saturation not discussed.<br>Relationship between researcher and participants was not adequately considered.<br>Credibility of findings not addressed when reporting the findings.	
	Weiner, 2009[64]	16 Medium	Analysis by single researcher and potential bias not addressed.<br>Data saturation not discussed.<br>Relationship between researcher and participants was not adequately considered.<br>Credibility of findings not addressed when reporting the findings.	
	Weiner, 2011[65]	16 Medium	Analysis by single researcher and potential bias not addressed.<br>Data saturation not discussed.<br>Relationship between researcher and participants was not adequately considered.<br>Credibility of findings not addressed when reporting the findings.	

For peer review only - http://bmjopen.bmj.com/site/about/guidelines.xhtml

**15**	Senior et al, 2002[61]	12 Low	Lack of details provided about the study methodology or rigour of analysis process.<br>Ethical issues not addressed.<br>Data saturation not discussed.<br>Credibility of findings or limitations of study methodology not addressed when reporting findings.<br>Relationship between researcher and participants was not adequately considered.	All motivated to participate in research as recruited from ongoing trial.<br>All lived in central London.<br>All clinical diagnosis, but 5 had DNA diagnosis confirmed and 2 had negative DNA test.

Additional references (To those listed in main manuscript)

112. Santiago-Delefosse M, Gavin A, Bruchez C, Roux P, Stephen SL. Quality of qualitative research in the health sciences: Analysis of the common criteria present in 58 assessment guidelines by expert users. Social Science & Medicine. 2016;148:142-5

For peer review only - http://bmjopen.bmj.com/site/about/guidelines.xhtml

Supplementary File 5: Full details of thematic synthesis methodology

Stage 1: Line by line coding

In our protocol we originally planned to analyse the extracted data according to our review questions regarding factors influencing adherence to treatment. However, few studies directly addressed this question, therefore the authors put these review questions to one side for the data extraction process and revisited them for the coding stage.

Two reviewers (AS, FJK) had previously read all papers independently for the critical appraisal stage. They were therefore familiar with the papers and had discussed them. At this stage they independently reread and coded, on paper, the extracted data from seven papers. The process involved line by line coding of the extracted data in which each line of text was assigned a free code according to its meaning and content. The codes were inductively created in response to the findings uncovered. The two reviewers then met to discuss and compare their findings before then deciding upon a preliminary coding frame which they then used when coding the extracted data from three further papers independently. In addition, new codes were created when necessary and the reviewers met again to discuss the findings, making revisions to the coding frame. One reviewer (FJK) then independently coded the extracted data from each paper using NVivo software. The coding frame was modified and added to throughout this process, with any changes made discussed with a second reviewer (AS). By this stage, no new codes were being identified, but some codes were consolidated into one code and others given more clarification about their meaning. A copy of the finalised coding frame is available to view (Appendix 1). A second reviewer (AS) performed secondary coding on 10% of the papers (three papers) before meeting with FJK to compare findings and ensure consistency of interpretation.

This line by line coding facilitated the translation of concepts from one study to another- a key component of qualitative synthesis. Most sentences were categorised using more than one code as a result of having content which had more than one possible meaning e.g. ‘perceived risk’ and ‘relative risk’ or ‘perceived seriousness’ and ‘emotional impact’.

Stage 2: Development of descriptive themes

This stage involves the development of initial descriptive themes based upon the raw data that closely reflect the aggregative findings of the included studies.[40] The two reviewers who had carried out the coding (AS, FJK) met with a third reviewer (EW) to discuss the findings

For peer review only - http://bmjopen.bmj.com/site/about/guidelines.xhtml

of the coding process. One reviewer (FJK) produced summary reports of each of the 19 identified free codes which provided an overview of the findings across the papers including illustrative quotes and disconfirming cases. The summaries were descriptive in nature and avoided any interpretation. These summaries formed the basis of discussion between the three reviewers. At this second stage, the discussion was carried out in the context of the first research question- what are the experiences and beliefs of individuals' in relation to their condition, its associated morbidity and mortality risk and treatment?'. The discussion was exploratory in nature and no *priori* framework was imposed upon the findings at this stage. The aggregative findings of the studies, as consolidated in the code summaries, were deliberated, with examination of any similarities, differences and relationships between codes explored. From this discussion, 20 descriptive themes were identified. These descriptive themes were reflective of prevalent and persistent findings across the studies. Some of these themes were reflective of original codes used in the coding process, others were new themes created to capture more specific and detailed aspects of the original findings of coding process. For example, the findings captured using the code 'family influence' were further categorised into the descriptive themes 'parental influence upon treatment related behaviours' and 'FH and its treatment becomes normalised within families'.

One reviewer (FJK) then produced a draft summary of these descriptive themes which was reviewed and discussed with AS and EW before a final version was agreed upon.

Stage 3: Development of analytical themes

The generated descriptive themes captured and aggregated the beliefs and experiences of individuals with FH in relation to their condition and its treatment. The third stage of thematic synthesis aims to go beyond the primary content of the original papers to generate additional concepts or understandings.[40] This is considered an essential component of any qualitative synthesis methodological approach.[42] In this review, this meant using the descriptive themes to answer our research questions regarding how these beliefs and experiences may influence an individuals' adherence to treatment and to identify any enablers and/or barriers to this.

This was achieved by first examining each descriptive theme individually in the context of treatment adherence through consideration of the relationship between the content captured in each descriptive theme and individuals ability and/or inclination to adhere to treatment. Secondly, any relationships between the descriptive themes were explored to identify

For peer review only - http://bmjopen.bmj.com/site/about/guidelines.xhtml

common factors. Each reviewer (FJK, AS, EW) carried this out independently before meeting as a group to discuss further. From these discussions, over-arching analytical themes were identified. These analytical themes were then deliberated in the context of identifying enablers and barriers to treatment adherence which could be used to inform clinical practice, policy development and research intervention design. The reviewers met on three occasions to discuss their findings collaboratively. It was an iterative process in which the analytical themes were modified until the reviewers felt they adequately explained all the initial descriptive themes and identified enablers and barriers to treatment.

For example, three of the descriptive themes related to the involvement of other family members in an individuals' experiences of having FH and its treatment (FH and its treatment become normalised within families, parental influence upon treatment related behaviours and desire to protect children). From these descriptive themes, the reviewers identified the importance of the behaviours and beliefs of other family members upon an individuals ability and receptivity to adhering to treatment. This finding was captured in the analytical theme entitled 'family influence'. From this analytical theme, the reviewers identified two enablers to treatment adherence. These enablers were the delivery of care and treatment advice through family-based clinics and the commencement of treatment from a young age.

DRAFT
For peer review only

For peer review only - http://bmjopen.bmj.com/site/about/guidelines.xhtml

Appendix 1: Finalised coding frame

Code	Brief Description
Understanding/biological knowledge of FH	Account/description of what FH is, their understanding of its aetiology, its genetic transmission, its effect upon their body, any symptoms and any associated short and long term health implications
Perceived risk	The perceived risk of FH as a condition. Their thoughts/beliefs of short and long term health consequences of FH. Both genetic and behavioural associated risk.
Perceived seriousness	How serious/important FH and/or it's associated health consequences are believed to be
Family history	Account/description of family history of FH diagnosis, treatment and/or adverse outcomes such as death/serious illness
Life events	Significant milestones/occasions in life i.e. becoming parent, leaving school, getting married, ageing
Co-morbidities	Other illnesses/conditions that are not FH
Relative risk	Participant compares own risk to that of another person (family member, peer, abstract person) or to risk associated with another condition/illness
Management of condition	Account/description of the use/role of medicine or lifestyle in the treatment of FH.
Perceived efficacy of treatment	Perceptions/beliefs of the effectiveness of treating FH (medication, lifestyle and other)
Self-efficacy	The perception of an participant upon their own ability to follow treatment recommendations
Enablers and barriers for treatment	Any factors that help, enable, motivate OR Any factors that demotivate, stop or hinder a participant to seek and/or follow treatment advice
Ownership/personal responsibility	How a participant reflects/describes their perceived ownership of their condition and it's treatment. How much they perceive the condition to be their responsibility to manage/treat.
Emotional impact	Any emotion that FH diagnosis, management and/or associated health outcomes evokes in participants. Includes perceived stigma.
Impact on life	Any change participant has made to their life (everyday or longer term) as a result of their diagnosis of FH or its treatment
Professional support	Account/description of any involvement of healthcare professionals and/or medical procedures
Social support	Account/description of the role of family and/or friends in a patients' experience of their condition and it's management. practical or emotional support that individuals receive with regard to managing FH - i.e, treatment adherence.
Family influence	The influence of participants family upon their decision and ability to seek/adhere to treatment. Individuals' awareness of how others in their family network have dealt with screening and treatment and making decisions based on what other family members have done.
Information/help seeking	Accounts/descriptions of information or resources that participants would find useful
Parental views	Accounts/descriptions/thoughts/beliefs of parents in relation to their children.

For peer review only - http://bmjopen.bmj.com/site/about/guidelines.xhtml
